# Improving Illumina assemblies with Hi‐C and long reads: An example with the North African dromedary

**DOI:** 10.1111/1755-0998.13020

**Published:** 2019-05-17

**Authors:** Jean P. Elbers, Mark F. Rogers, Polina L. Perelman, Anastasia A. Proskuryakova, Natalia A. Serdyukova, Warren E. Johnson, Petr Horin, Jukka Corander, David Murphy, Pamela A. Burger

**Affiliations:** ^1^ Department of Integrative Biology and Evolution Research Institute of Wildlife Ecology, Vetmeduni Vienna Vienna Austria; ^2^ Intelligent Systems Laboratory University of Bristol Bristol UK; ^3^ Institute of Molecular and Cellular Biology SB RAS and Novosibirsk State University Novosibirsk Russia; ^4^ The Walter Reed Biosystematics Unit, Smithsonian Institution Museum Support Center MRC‐534 Suitland Maryland; ^5^ Department of Animal Genetics, Faculty of Veterinary Medicine, Ceitec VFU, RG Animal Immunogenomics University of Veterinary and Pharmaceutical Sciences Brno Czech Republic; ^6^ Department of Biostatistics University of Oslo Oslo Norway; ^7^ Department of Mathematics and Statistics University of Helsinki Helsinki Finland; ^8^ Bristol Medical School: Translational Health Sciences, Molecular Neuroendocrinology Research Group University of Bristol Bristol UK

**Keywords:** chromosome conformation capture, chromosome mapping, dromedary, genome annotation, genome assembly, scaffolding

## Abstract

Researchers have assembled thousands of eukaryotic genomes using Illumina reads, but traditional mate‐pair libraries cannot span all repetitive elements, resulting in highly fragmented assemblies. However, both chromosome conformation capture techniques, such as Hi‐C and Dovetail Genomics Chicago libraries and long‐read sequencing, such as Pacific Biosciences and Oxford Nanopore, help span and resolve repetitive regions and therefore improve genome assemblies. One important livestock species of arid regions that does not have a high‐quality contiguous reference genome is the dromedary (*Camelus dromedarius*). Draft genomes exist but are highly fragmented, and a high‐quality reference genome is needed to understand adaptation to desert environments and artificial selection during domestication. Dromedaries are among the last livestock species to have been domesticated, and together with wild and domestic Bactrian camels, they are the only representatives of the Camelini tribe, which highlights their evolutionary significance. Here we describe our efforts to improve the North African dromedary genome. We used Chicago and Hi‐C sequencing libraries from Dovetail Genomics to resolve the order of previously assembled contigs, producing almost chromosome‐level scaffolds. Remaining gaps were filled with Pacific Biosciences long reads, and then scaffolds were comparatively mapped to chromosomes. Long reads added 99.32 Mbp to the total length of the new assembly. Dovetail Chicago and Hi‐C libraries increased the longest scaffold over 12‐fold, from 9.71 Mbp to 124.99 Mbp and the scaffold N50 over 50‐fold, from 1.48 Mbp to 75.02 Mbp. We demonstrate that Illumina de novo assemblies can be substantially upgraded by combining chromosome conformation capture and long‐read sequencing.

## 1 INTRODUCTION

Technlogical advances in sequencing have enabled researchers to assemble thousands of eukaryotic genomes. More than 82% of the ~4,300 eukaryotic genomes in the National Center for Biotechnology and Information (NCBI) Assembly database with assembly reports have been assembled using short‐ and/or long‐insert (mate‐pair) libraries sequenced with Solexa/Illumina's “Sequencing‐By‐Synthesis” technology (Bonetta, 2006; Kitts et al., 2016; Table S1). These genomes are typically in a draft form, consisting of tens of thousands of scaffolds that comprise the majority of the assembly. Long‐insert libraries greater than 8 Kbp are needed to span long interspersed nuclear elements (LINEs), some of the most common repetitive elements in eukaryotic genomes (Sotero‐Caio, Platt, Suh, & Ray, 2017; Treangen & Salzberg, 2011; van Heesch et al., 2013). However, even 20–25 Kbp long‐insert libraries cannot span and thus resolve repetitive elements such as long segmental duplications in eukaryotic genomes (Bailey, 2004; Feng et al., 2017; Treangen & Salzberg, 2011).

Newer high‐throughput laboratory methods are beginning to overcome the limitations of traditional long‐insert libraries, and these new libraries can extend across repetitive regions enabling the scaffolding and ordering of previously unscaffolded contigs. One method, Hi‐C, is a type of chromosome conformation capture or proximity ligation method. The method involves DNA regions that are in close proximity three‐dimensionally and cross‐linked in vivo, digested with restriction enzymes, repaired with biotinylated residues, and ligated together resulting in DNA regions that have various chromatin interactions but are located close together on the same synthetic molecule. These synthetic molecules can then be sheared, enriched for interacting regions using streptavidin beads, and ultimately sequenced using Illumina short‐insert libraries in a higher‐throughput fashion compared to laborious bacterial artificial chromosome (BAC) and fosmid end sequencing (Lieberman‐Aiden et al., 2009). Resulting Hi‐C paired‐end reads can be mapped to de novo genome assemblies and used to scaffold and order contigs, creating super scaffolds in the size range of chromosomes (Bickhart et al., 2017; Dudchenko et al., 2017; Kaplan & Dekker, 2013; Korbel & Lee, 2013). Another proximity ligation method, Chicago libraries, has much in common with Hi‐C except Chicago libraries are constructed in vitro (Putnam et al., 2016) and are available as a commercial service from Dovetail Genomics (Santa Cruz, California, USA). Both Hi‐C and Dovetail Chicago libraries have been successfully used in creating super scaffolds to improve the continuity of numerous eukaryotic genomes (Kaplan & Dekker, 2013; Korbel & Lee, 2013; Moll et al., 2017).

Eukaryotic genome assemblies have been further enhanced by long‐read sequencing technologies from Pacific Biosciences (PacBio, Menlo Park, CA, USA; Eid et al., 2009) and Oxford Nanopore Technologies (Oxford Nanopore, Oxford, UK; Venkatesan & Bashir, 2011). These technologies generate much longer sequences, but raw reads have higher error rates and are more prone to insertions/deletions (indels) than Illumina reads (Jain, Olsen, Paten, & Akeson, 2016; Salmela & Rivals, 2014). Both Oxford Nanopore and PacBio overcome the problems of error‐prone raw reads by generating a consensus sequence either on the level of the instrument whereby DNA molecules in PacBio sequencers are read multiple times (i.e., circular consensus sequences) or after the sequences have been generated by PacBio or Oxford Nanopore sequencers. Ultimately, these long‐read technologies generate longer sequences that can span repetitive regions, enabling the assembly of longer contigs that can be later error corrected and/or scaffolded into high‐quality eukaryotic genome assemblies using traditional long‐insert, Hi‐C, or Dovetail Chicago libraries (Jiao et al., 2017; Miller et al., 2017; Passera et al., 2018).

High‐quality genomes resulting from long reads and/or Hi‐C libraries have improved gene sequence completeness for evolutionary studies and can be used to understand what genetic variation influences phenotypic traits to benefit evolutionary ecology and selective breeding. For example, the latest assembled goat genome has taken advantage of long‐read sequences and Hi‐C libraries in its assembly (Bickhart et al., 2017). Additionally, long‐read sequence assembly of great ape genomes facilitated high‐resolution comparative analyses between humans and great apes (Kronenberg et al., 2018). Genetic variation that influences phenotypic traits can be identified with genome‐wide association studies (GWAS) that benefit from more contiguous assemblies. Contiguous assemblies have more variants per scaffold, which can improve genotype imputation (i.e., filling in missing genotype data) in GWAS (Davies, Flint, Myers, & Mott, 2016), and contiguous assemblies also permit searching genomes for genes located nearby variants that are significantly associated with phenotypes. For example, GWAS with contiguous genome assemblies identified candidate loci associated with tuberculosis susceptibility and recombination hot spots in wild boar and Soay sheep, respectively (Johnston, Bérénos, Slate, & Pemberton, 2016; Queirós, Alves, Vicente, Gortázar, & de la Fuente, 2018). GWAS and genomic selection are now routine approaches to improve selective breeding in agriculture and horticulture, for example to investigate beef and milk production traits in cattle (Sorbolini et al., 2017; Yue et al., 2017) and growth and fatness in pigs (Guo et al., 2017).

A contiguous genome assembly is not available for the dromedary (*Camelus dromedarius*), an important livestock species especially for dry and marginal ecoagricultural parts of the world. Dromedaries are among the last livestock species to have been domesticated, only around 3,000 years ago (Almathen et al., 2016; Uerpmann & Uerpmann, 2012). Traditionally, they have been bred as multi‐purpose animals (Abdussamad, Charruau, Kalla, & Burger, 2015), for milk, meat, hides and wool, and for endurance and transport; only recently stronger selection has begun for fast, narrow‐bellied racing camels (Faye, Abdallah, Almathen, Harzallah, & Al‐Mutairi, 2011). Thus, dromedaries present a very interesting model to study the “initial stages” of domestication, where potential signals of selection for tameness and tolerance of humans are not overlaid by stronger signatures of artificial selection for economic traits, as seen in specific meat and milk breeds from other livestock. In terms of evolutionary significance, dromedaries form together with their sister taxa, the domesticated Bactrian camel (*Camelus bactrianus*) and the highly‐endangered wild two‐humped camels (*Camelus ferus*), the tribe of Camelini (Old World camels). Next to the New World camels (Lamini) they are the only representatives of the suborder Tylopoda. Thus, dromedary breeders and evolutionary biologists would benefit from a high‐quality dromedary reference genome. Although draft genome assemblies from North African and Arabian dromedaries have been established, respectively (Fitak, Mohandesan, Corander, & Burger, 2016; Wu et al., 2014); these genome assemblies are highly fragmented, and scaffolds are not assigned to chromosomes.

Here we describe our efforts to improve the North African dromedary reference genome. We used Chicago and Hi‐C sequencing libraries from Dovetail Genomics to resolve the placement and order of previously de novo assembled contigs from Illumina short‐ and long‐insert libraries (Fitak et al., 2016), producing almost chromosome‐level scaffolds for which we filled in gaps using PacBio long reads, mapped scaffolds to chromosomes, and annotated the resulting assembly.

## 2 MATERIALS AND METHODS

### 2.1 Brief summary of the CamDro2 assembly process

We scaffolded the existing Illumina‐only assembly (Fitak et al., 2016; GenBank accession: GCA_000803125.1) with Dovetail Chicago data, improved the Chicago assembly by scaffolding with Hi‐C data, filled in gaps in the Hi‐C assembly with PacBio reads, then filled in gaps and polished the assembly with Illumina reads used to de novo assemble GCA_000803125.1. An overview of the CamDro2 assembly process is given in Figure S1.

### 2.2 The original North African dromedary genome assembly

The original North African dromedary genome assembly was created from a female dromedary “Waris” (Fitak et al., 2016; GenBank accession: GCA_000803125.1) owned by the First Austrian Camel Riding School, stemming from the Canary Islands with North African ancestry. Briefly, two types of Illumina libraries were generated and sequenced from DNA extracted from whole blood, which was collected commensally during veterinary diagnostic treatment with the owner's consent: 500 bp (short‐insert, 100 bp paired‐end reads) and 5 Kbp (long‐insert/mate‐pair, 50 bp paired‐end reads) libraries. Short‐ and long‐insert reads were trimmed with popoolation v. 1.2.2 (Kofler et al., 2011), and short‐insert reads were error‐corrected with quake v 0.3.5 (Kelley, Schatz, & Salzberg, 2010). Short‐ and long‐insert reads were simultaneously assembled with abyss v. 1.3.6 (Simpson et al., 2009) with a k‐mer value of 64 resulting in the longest scaffold N50. Only scaffolds greater than 500 bp were retained for the final North African dromedary (Fitak et al., 2016; GenBank accession: GCA_000803125.1) assembly, hereafter CamDro1.

### 2.3 Dovetail Chicago and Hi‐C libraries

Dovetail Genomics created Chicago and Dovetail Hi‐C libraries from a low‐passage cell culture line (Perelman, Pichler, Gaggl, & Larkin, 2018) derived from ear fibroblasts of the same dromedary “Waris” used in CamDro1. The fibroblast cells were retrieved from a diagnostic skin scraping for parasites, and the owner agreed on using the leftover material for research purposes. Dovetail Genomics created three Chicago and three Hi‐C libraries with the *DpnII* restriction enzyme, sequenced these libraries on six lanes of an Illumina HiSeq sequencer, and then scaffolded the CamDro1 assembly using the HiRise pipeline (Putnam et al., 2016). To do so, first, the CamDro1 assembly was scaffolded using Dovetail Chicago data. The Chicago assembly was then improved by scaffolding with Hi‐C data creating a Hi‐C assembly.

### 2.4 PacBio long‐read sequencing

We extracted high molecular weight DNA from the same low‐passage cell line used to create Dovetail Chicago and Hi‐C libraries. Briefly, the high molecular weight DNA was extracted by lysing freshly harvested cells in lysis buffer, followed by phenol chloroform extraction and precipitation. Throughout the whole extraction process, the DNA was manipulated gently to preserve high molecular weight. From this DNA, the Vienna BioCenter Core Facilities NGS Unit (Vienna, Austria, www.vbcf.ac.at) created a PacBio library for the PacBio Sequel sequencer and sequenced this library on five 1M v2 PacBio Sequel SMRT Cells using PacBio Sequel 2.1 sequencing reagents.

### 2.5 Additional assembly steps

We used bamtools v. 2.5.0 (Barnett, Garrison, Quinlan, Strömberg, & Marth, 2011) to extract FASTQ sequences from PacBio Sequel subread BAM (binary alignment map) files. Because quality values for subreads from the PacBio Sequel are given a Phred quality score of 0, we artificially assigned a Phred score of 30 to all bases for input into pbjelly v. 15.8.24 (English et al., 2012) to fill in gaps in the Hi‐C assembly. We polished the PBJelly assembly with pilon v. 1.22 (Walker et al., 2014; see Supplementary Methods for settings) employing the same trimmed and error‐corrected Illumina short‐insert sequences used for the assembly of CamDro1 by Fitak et al. (2016; Sequence Read Archive accession: SRR2002493) to correct SNPs and indels. Next, we filled in gaps present in the Pilon assembly with abyss sealer v. 2.0.2 (Jackman et al., 2017) using the same error‐corrected Illumina reads and default settings except for a bloom filter size of 40 GB and multiple *K* values from 90 to 20 in increments of 10. Finally, we polished the abyss assembly with pilon (see Supplementary Methods for settings) once again with the same error‐corrected Illumina reads, fixing any SNPs and indels that were not accounted for in the first round of polishing but also filling in gaps. We refer to this as the CamDro2 assembly.

### 2.6 K‐mer analysis and dot plot

We compared 27‐mers present in the error‐corrected Illumina short‐insert sequences and the CamDro2 assembly using kat v. 2.3.4 (Mapleson, Garcia Accinelli, Kettleborough, Wright, & Clavijo, 2017; see Supplementary Methods for settings) to evaluate the proportion of the sequencing reads, duplication rates, and heterozygosity present in the CamDro1 and CamDro2 assemblies.

To assess the level of disagreement between CamDro1 and CamDro2, we made a whole‐genome alignment with minimap2 v. 2.15 (Li, 2018) using the “asm5” preset. We then used d‐genies v. 1.2.0.1 (Cabanettes & Klopp, 2018) to generate a dot plot for the alignment by using the contig sorting function and filtering out matches with ≤0.001% dot plot width and identity ≤0.75.

### 2.7 RNA‐Seq mapping

To assess the quality of the new assembly, we aligned 10 sets of paired‐end RNA‐Seq reads (Alim et al., 2019) to the original assembly (CamDro1), to the new assembly (CamDro2), and to several controls: *C. dromedarius* (RefSeq version ‐ GCA_000767585.1), *C. bactrianus* (GCA_000767855.1), *C. ferus* (GCA_000311805.2) and *Bos taurus* (cattle) (GCA_000003055.3). The 10 RNA‐Seq data sets comprise a 2 × 2 factorial experiment: summer versus winter seasons and supraoptic nucleus (SON) versus neurointermediate lobe (NIL) brain tissues, with *n* = 3 replicates in each class. Tissue was homogenized and extracted using in Trizol/chloroform (ThermoFisher), and purified using the RNeasy MiniKit (Qiagen). The library template was prepared using a ribosome depletion protocol (Ribo‐Zero Gold; Illumina) and libraries prepared using TruSeq Stranded protocol (Illumina). Samples were multiplexed into lane pools with an 8 pm concentration and sequenced (100 bp paired‐end reads with an average 134 bp insert size) to a depth of > 35 million reads using an Illumina HiSeq 2500. Two of the 12 replicates were rejected for insufficient quality. We used tophat v. 2.0.9 (Kim et al., 2013) with default settings to align reads to each genome and report overall alignment rate (default output of Tophat) within each class.

### 2.8 Chromosome mapping

We used blastn v. 2.2.31+ (Altschul, 1990) with an E value ≤1e−30 and max hsps 1 to map 4,981 RH (radiation‐hybrid) probe sequences assigned to *Vicuna (Lama) pacos* (alpaca) chromosomes (W. E. Johnson *unpublished data*; Avila et al., 2014) to CamDro2 assembly scaffolds. Briefly, the unpublished RH probe sequences come from a range of sources: *V. pacos* cDNA and microsatellite sequences, custom designed primers, oligos based on bioinformatic screening of 2× *V. pacos* genome, and homologous (*V. pacos*) and heterologous (*B. taurus*) oligos from SNP chips. Camelids (*C. dromedarius*, *C. bactrianus*, *C. ferus, Lama glama*, *L. guanicoe, V. pacos*, and *V. vicuna*) have highly conserved karyotypes and share the same diploid number (2*n* = 74) and syntenic groups (Avila et al., 2014; Balmus et al., 2007). Balmus et al. (2007) used dromedary whole‐chromosome painting probes to look for differences in dromedary, *L. guanicoe*, and *C. bactrianus* chromosome sets. They found each dromedary painting probe painted one single chromosome in *L. guanicoe* and *C. bactrianus* indicating intact syntenic groups with no fusion or fission events. They only found differences in the size and composition of heterochromatin blocks, which are repeats not associated with our alpaca RH probe markers. Avila et al. (2014) mapped alpaca BAC markers onto dromedary chromosomes and also found no fission or fusion events and stated exceptional conservation of syntenies between *V. pacos* and dromedary. The *V. pacos* BAC map of Avila et al. (2014) is integrated with the dromedary painting map of Balmus et al. (2007) meaning *V. pacos* and dromedary maps have the same chromosome (syntenic group) order. Overall chromosome painting and BAC mapping provide sufficient physical mapping evidence to conclude that chromosomes of *V. pacos* and dromedary (as well as of other camelids) have one‐to‐one correspondence and follow the same nomenclature and order. We inferred dromedary chromosome numbers by blasting RH probes for each *V. pacos* chromosome against CamDro2 scaffolds, keeping only the highest E value hit for each RH probe, and assigning chromosome number based on the CamDro2 scaffold with the most blast hits for each *V. pacos* chromosome RH probe set.

To further assess the validity of *V. pacos* chromosome RH probe sets to infer dromedary chromosome numbers, we made a whole‐genome alignment between CamDro2 and a recently made public *V. pacos* Hi‐C assembly (https://www.dnazoo.org/assemblies/Vicugna_pacos; hereafter Alpaca assembly; Dudchenko et al., 2017, 2018) with Minimap2 using the “asm5” preset. We used D‐GENIES to generate a dot plot for the alignment by using the contig sorting function and filtering out matches with ≤0.001% dot plot width and identity ≤0.5. Before alignment, we reverse complemented CamDro2 chromosomes 1, 3, 4, 6, 7–10, 12–14, 26, 35, and X as these chromosomes were in the opposite orientation to matching Alpaca assembly scaffolds.

We repeated blastn mapping of our *V. pacos* RH probe sequences to the Alpaca assembly to assign putative chromosome numbers to this assembly.

### 2.9 Annotation

We annotated CamDro2 scaffolds greater than 10 Kbp with maker v. 2.31.9 (Cantarel et al., 2008; Holt & Yandell, 2011). We performed two MAKER runs iteratively: the gene predictions from MAKER run 1 were used to train augustus v. 3.3 (Stanke et al., 2006) for the MAKER run 2 using Augustus's autoAug.pl script (see Supplementary Methods for settings). For both MAKER runs, we masked repetitive regions with repeatmasker v. open‐4.0.7 (http://www.repeatmasker.org) against the entire Dfam_Consensus release 20170127 database. For each run, we included ab initio gene predictions from genemark‐es v. 4.33 (Lomsadze, 2005), expressed sequence tag (EST) transcripts, and protein sequences.

For ESTs, we assembled transcripts from two dromedary RNA‐Seq experiments (Sequence Read Archive accession: SRP017619; Alim et al., 2019). We performed adapter and quality trimming on raw demultiplexed paired‐end reads using bbduk v. 37.25 (https://sourceforge.net/projects/bbmap/), using the following settings: ktrim = r, k = 23, mink = 11, hdist = 1, tpe, tbo, qtrim = rl, trimq = 15. We then mapped quality and adapter trimmed reads to the CamDro2 assembly using hisat v. 2.1.0 (Kim, Langmead, & Salzberg, 2015) using a maximum intron length of 100,000. Reads were assembled into transcripts using stringtie v. 1.3.3b (Pertea et al., 2015; see Supplementary Methods for settings) and extracted using gffread v. 0.9.9 (https://github.com/gpertea/gffread). For alternative ESTs, we processed transcriptome reads from *C. bactrianus* (Sequence Read Archive accession: SRP014573) with HiSat, StringTie, and Gffread as before but mapped quality controlled reads to the *C. bactrianus* genome (GenBank accession: GCF_000767855.1). For proteins, we combined predicted proteins from *B. taurus*, *C. bactrianus*, and *V. pacos* (GenBank accessions [NCBI annotation release]: GCF_000003055.6 [105], GCF_000311805.1 [100], and GCF_000164845.2 [101], respectively).

We also included MAKER predicted proteins with an annotation edit distance (AED) <0.75 from the CamDro1 assembly (Fitak et al., 2016). For the first MAKER run, we trained Augustus using busco v. 3.0.2 (Simão, Waterhouse, Ioannidis, Kriventseva, & Zdobnov, 2015) searching for Eukaroyota orthodb v. 9.1 genes (Zdobnov et al., 2017) against CamDro2. For both MAKER runs, we used a dromedary specific repeat library created with repeatmodeler v. open‐1.0.10 (http://www.repeatmasker.org) with the CamDro2 assembly as input. We filtered the repeat library from RepeatModeler to remove known UniProt/Swiss‐Prot release 2017_10 (Boutet et al., 2016) proteins using protexcluder v. 1.1 (Campbell et al., 2014).

After the second MAKER run, we only retained genes, transcripts, and proteins with AED ≤ 0.50. Next, we predicted putative gene function with diamond v. 0.9.19 (Buchfink, Xie, & Huson, 2015) searches against the UniProt/TrEMBL release 2018_04 database using an E value cutoff of 1e−6. We also mapped proteins predicted by MAKER against the same UniProt/TrEMBL database using diamond and generated a frequency polygon of the query sequence length (predicted proteins) divided by the subject sequence length (UniProt/TrEMBL proteins) to assess if predicted proteins were truncated (query sequence length divided by the subject sequence length <1.0) due to uncorrected indels introduced by PacBio reads that might interrupt reading frames affecting protein translation (Watson & Warr, 2019). We also generated 75 random sets of 250 transcripts with AED ≤ 0.25 to test the specificity and sensitivity of Augustus ab initio models used during the first and second MAKER runs.

As part of the assessment of CamDro2, we also annotated CamDro1 using the same MAKER settings and input files used for CamDro2’s annotation. We then summarized annotations (i.e., length distributions of genes, mRNAs, exons, introns, and CDS [coding sequences]) with genome annotation generator (Geib et al., 2018).

## 3 RESULTS

### 3.1 Dovetail Chicago and Hi‐C libraries

**Figure 1:**
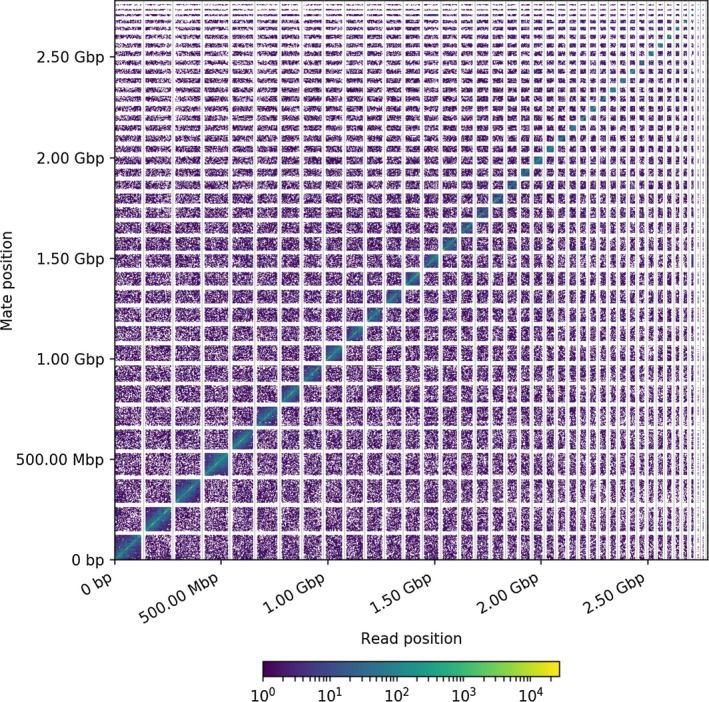
Dovetail Genomics’ Hi‐C linkage density plot for Hi‐C reads mapped to the Hi‐C assembly. X‐ and Y‐axes give the cumulative mapping positions of the first and second read in a read pair respectively, grouped into bins. The colour of each square gives the number of reads pairs within that bin. Grey vertical and white horizontal lines separate borders between scaffolds. Only scaffolds >1 Mbp are shown [Colour figure can be viewed at wileyonlinelibrary.com]

There were 384 million read pairs (2 × 151 bp reads) from Chicago libraries with 56× physical coverage to CamDro1. Likewise, there were 413 million read pairs (2 × 151 bp reads) from Hi‐C libraries with 60× physical coverage to the Chicago assembly (i.e., the CamDro1 assembly scaffolded by Chicago data). The CamDro1 assembly had 35,752 scaffolds containing 133,158 contigs, and scaffold N50 and N90 were 1.482 Mbp and 0.260 Mbp, respectively (Table 1). After applying Dovetail Chicago and then Dovetail Hi‐C data, the number of total scaffolds decreased from 35,752 to 24,424 (Table S2). The new scaffold N50 and N90 were 73.028 Mbp and 24.048 Mbp, respectively in the Hi‐C assembly (Chicago assembly scaffolded by Hi‐C data, Table S2). Dovetail Genomics’ HiRise pipeline generated a Hi‐C linkage density plot between the Hi‐C assembly and the Hi‐C read pairs (Figure 1). Considering super scaffolds >1 Mbp are allocated in separate shading blocks, this suggests the Hi‐C assembly is well assembled (Figure 1).

**Table 1:** Assembly statistics for the original North African dromedary assembly (CamDro1) (Fitak et al., 2016; GenBank accession: GCA_000803125.1); the North African dromedary assembly after improvement (CamDro2) by Chicago and Dovetail Hi‐C sequencing libraries, followed by filling in gaps with 11x coverage PacBio Sequel reads using pbjelly (English et al., 2012), next polishing with Illumina short‐insert libraries using pilon (Walker et al., 2014), and then filling in gaps with Illumina short‐insert libraries using abyss sealer (Jackman et al., 2017), and polishing again but also filling in gaps with Pilon; and for comparison the Arabian dromedary assembly (Wu et al., 2014; GCA_000767585.1)

	Assembly		
	Original North African Dromedary (CamDro1)	Improved North African Dromedary (CamDro2)	Arabian Dromedary
Total size	2,055,063,633	2,154,386,959	2,004,047,047
Gap length	53,035,436	20,341,506	22,407,814
Scaffolds			
Number	35,752	23,439	32,572
Longest	9,719,801	124,992,380	23,736,781
N90^a^	260,185	24,922,612	689,795
L90^b^	1,592	31	594
N50^a^	1,482,444	75,021,453	4,188,677
L50^b^	393	11	132
Contigs^c^			
Number	133,158	45,969	93,701
Longest	413,938	9,491,684	896,174
N90	11,508	177,667	17,513
L90	42,697	1,944	25,175
N50	50,278	1,333,231	88,36
L50	11,378	423	6,074
Single‐copy BUSCOs^d^	3,820	3,851	3,811
Duplicated BUSCOs	22	24	19
Fragmented BUSCOs	164	133	178
Missing BUSCOs	98	96	96
Proportion of complete BUSCOs	0.936	0.944	0.933

^a^ N90/N50 are the scaffold or contig lengths such that the sum of the lengths of all scaffolds or contigs of this size or larger is equal to 90/50% of the total assembly length.

^b^ L90/L50 are the smallest number of scaffolds or contigs that make up at least 90/50% of the total assembly length.

^c^ Using minimum gap length of 25 bp.

^d^ BUSCOs: Benchmarking Universal Single‐Copy Orthologs (Simão et al., 2015) are mammalian BUSCOs from orthodb v. 9.1 genes (Zdobnov et al., 2017).

### 3.2 Pacbio long‐read sequencing

From the five PacBio Sequel SMRT Cells, we generated 1,773,715 subreads totalling 24,832,304,602 bases or approximately 10.94x physical coverage for the k‐mer based dromedary genome size estimate of 2.27 Gbp (Table S3, Fitak et al., 2016).

### 3.3 Additional assembly steps

In the PBJelly assembly (i.e., Hi‐C assembly plus PacBio reads), there were 34,504 gaps (74,277 fewer than the Hi‐C assembly) accounting for only 22,348,368 bases. The PBJelly assembly increased in size by 94,136,380 bases, and 985 scaffolds were merged (Table S2). PBJelly increased the genome size by so much because Dovetail Genomics’ HiRise scaffolding pipeline assigns gaps of 1,000 bases when contigs are joined during scaffolding and many of the gaps were actually over‐filled (i.e., a gap of 1,000 bases from the Hi‐C assembly was filled in with more than 1,000 bases of sequence in the PBJelly assembly). The contig N50 and N90 increased from 50.229 Kbp and 11.505 Kbp to 1.043 Mbp and 0.127 Mbp, respectively. In the first round of Pilon error correction, Pilon corrected 359,441 SNPs and 564,275 short indels representing 757,963 bases. ABySS Sealer filled in 10,043 gaps totalling 1,448,040 bases (Table S2). In the second round of Pilon error correction, Pilon corrected 125,448 SNPs and 101,228 short indels representing 146,165 bases and filled in 1,931 gaps totalling 558,822 bases (Table S2).

**Figure 2:**
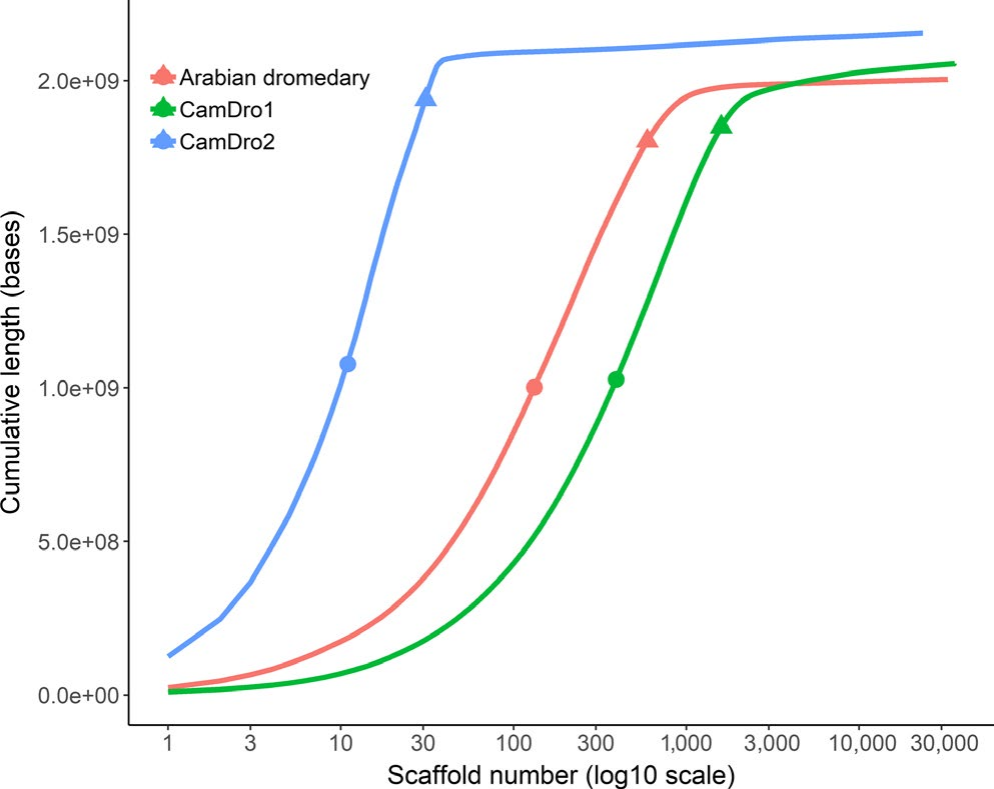
Cumulative assembly length for scaffolds of the original North African dromedary assembly (CamDro1; Fitak et al., 2016; GenBank accession: GCA_000803125.1); the North African dromedary assembly after improvement (CamDro2); and for the Arabian dromedary assembly (Wu et al., 2014; GCA_000767585.1). Circles and triangles indicate L50 and L90 values, respectively. L50/L90 are the smallest number of scaffolds that make up at least 50/90% of the total assembly length [Colour figure can be viewed at wileyonlinelibrary.com]

The longest scaffold in CamDro1 increased by 12‐fold in CamDro2, from 9.71 Mbp to 124.99 Mbp, and the scaffold N50 increased over 50‐fold, from 1.48 Mbp to 75.02 Mbp (Table 1). Likewise the longest scaffold and N50 of CamDro2 are more than 5‐ and 17‐fold greater than in the Arabian dromedary genome assembly (Table 1). The improved contiguity of CamDro2 is evident when comparing the N50 and N90 values and cumulative length distributions of scaffolds from CamDro1, CamDro2, and the Arabian dromedary genome assemblies (Figure 2).

### 3.4 K‐MER analysis and dot plot

KAT k‐mer analysis indicated a low proportion of sequencing data missing (i.e., black histogram bars) from both the CamDro1 (Figure S2a) and CamDro2 (Figure S2b) assemblies, suggesting that most of the sequencing reads were accounted for in both assemblies. Both assemblies were also mostly haploid (i.e., red bars) with low heterozygosity (Figures S2a and S2b; peak at k‐mer multiplicity of 15 for black bars). The CamDro2 assembly had a lower proportion of missing sequencing data than the CamDro1 assembly indicated by the lower amount of black shading between k‐mer multiplicity values 5 and 10, which is replaced by increased red shading at k‐mer multiplicity values near 1 (see panels below Figure S2a and b for magnified views).

**Figure 3:**
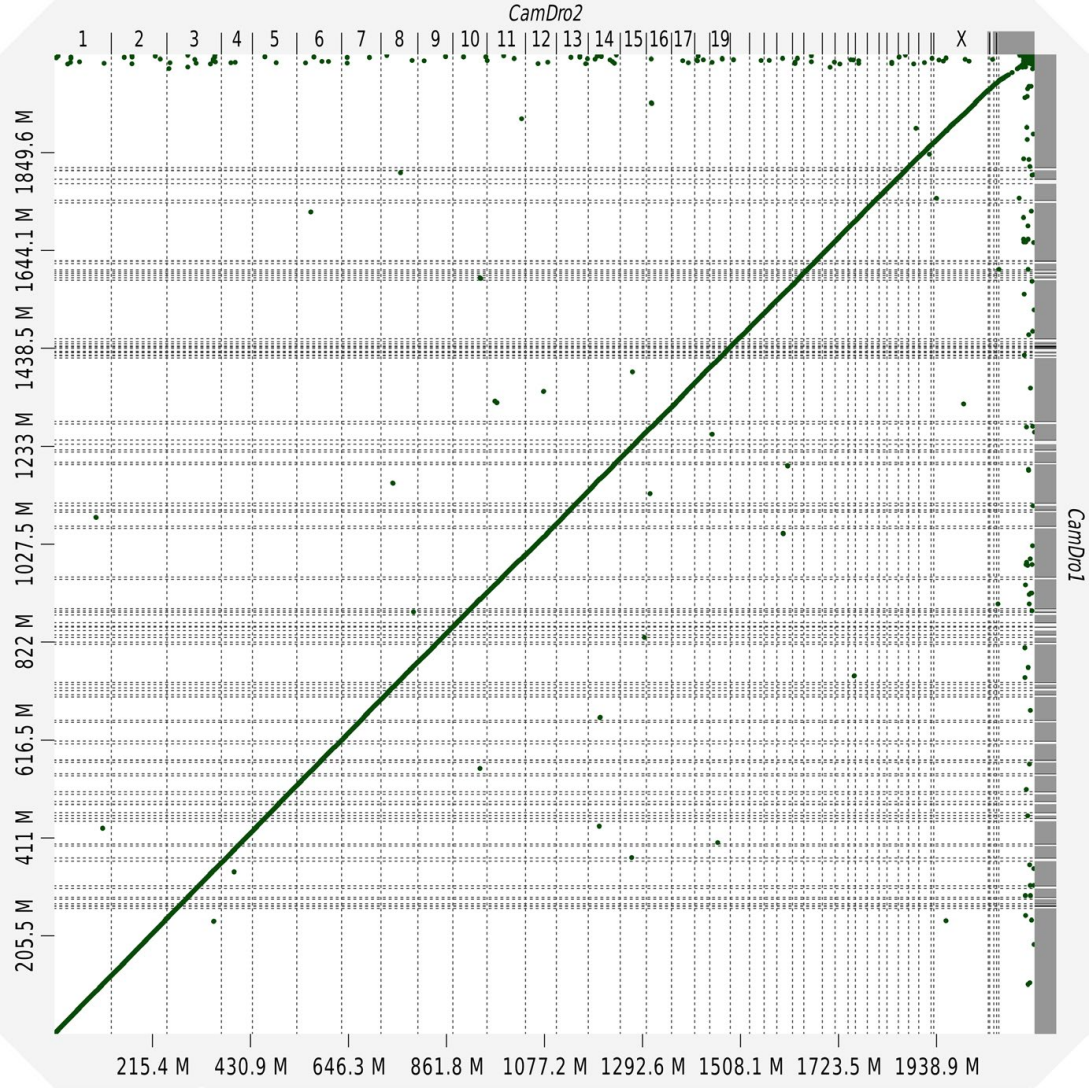
D‐GENIES (Cabanettes & Klopp, 2018) dot plot made with Minimap2 (Li, 2018) whole‐genome alignment between CamDro1 and CamDro2 assemblies. Contigs are sorted and matches are filtered out by size using ≤0.001% dot plot width and identity ≤0.75 [Colour figure can be viewed at wileyonlinelibrary.com]

The dot plot for the whole‐genome alignment between CamDro1 and CamDro2 shows very good correspondence and agreement between the two assemblies with little to no structural variations (Figure 3). We scoured the dot plot for signs of insertions, deletions, inversions, and repeats but could find very little evidence of structural variation even upon zooming into the plot. One example of structural variation between CamDro1 and CamDro2 is the 875 Kbp CamDro1 scaffold JWIN01032405.1, which was split and inverted relative to CamDro2 chromosome X (Figure S3). JWIN01032405.1 was split by Dovetail Genomics’ HiRise pipeline during scaffolding with Dovetail Chicago reads. We are not aware of other major structural variation, suggesting that synteny is likely conserved between CamDro1 and CamDro2.

### 3.5 RNA‐SEQ mapping

CamDro2 yielded 68.3% overall alignment rate, while the original *C. dromedarius* (RefSeq) assembly yielded 56.9% overall alignment rate, and CamDro1 yielded just 54.1%, comparable to *C. ferus*, also at 54.1% (Figure S4). As expected, *C. bactrianus* and *B. taurus* fared worse, at 51.5% and 11.6%, respectively.

### 3.6 Chromosome mapping

Of 4,891 *V. pacos* RH probes, 3,005 had hits with E values ≤ 1e−30 to CamDro2 scaffolds. For each chromosome set of *V. pacos* RH probes, nearly all of the probes (96 ± 7.7%; mean ± *SD*; Table S4) had best hits to a single CamDro2 scaffold, thus we were able to assign at least one super scaffold to each of the 37 chromosomes except the Y chromosome as the dromedary used in CamDro1 and CamDro2 was female. Chromosomes are denoted by numbers 1–36 and X in the CamDro2 assembly. There were 101,628,251 bases in scaffolds not assigned to chromosomes accounting for 4.72% of the assembly.

We found strong correspondence between CamDro2 and Alpaca scaffolds through a dot plot of the whole‐genome alignment (Figure S5). There are inversions in chromosomes 9, 16, 30, and 35 between the two assemblies (Figures S6–S9). These findings suggest there is strong conservation of chromosomal arrangement among CamDro2 and Alpaca assemblies. In summary, we were able to assign chromosomes 1–36 and X to the Alpaca assembly (Table S5). We could not assign the Y chromosome to the Alpaca assembly as we do not have an alpaca RH probe chromosome set for the Y chromosome.

### 3.7 Annotation

**Figure 4:**
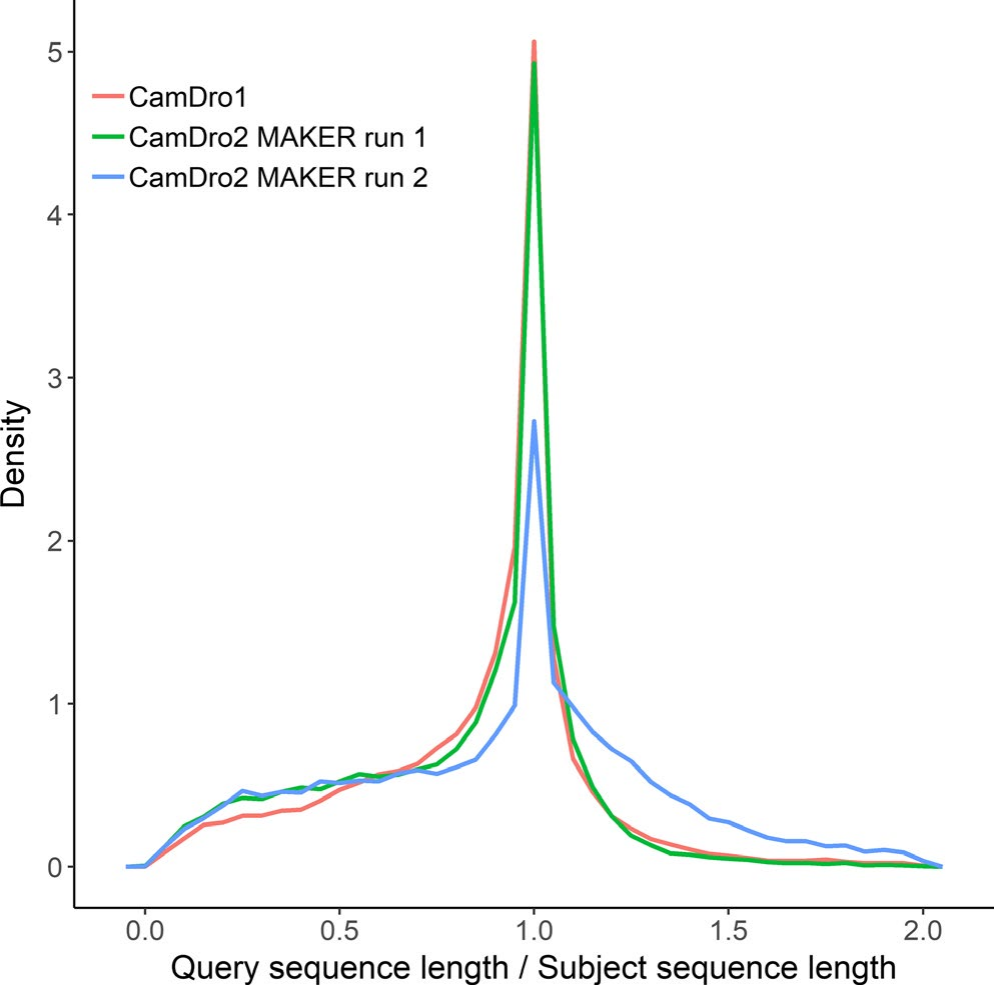
Frequency polygons of query sequence length (predicted proteins) divided by subject (UniProt/TrEMBL) sequence length for diamond (Buchfink et al., 2015) mapped maker (Holt & Yandell, 2011) predicted proteins against UniProt/TrEMBL release 2018_04 database for: (red line) the original North African dromedary genome (CamDro1; Fitak et al., 2016 predicted protein sequences; GenBank accession: GCA_000803125.1); (green line) the North African dromedary genome after adding ~11× PacBio sequencing reads (CamDro2) for MAKER run 1; and (blue line) MAKER run 2. Values near 1.0 are desired, indicating untruncated proteins due to lack of indels from PacBio reads [Colour figure can be viewed at wileyonlinelibrary.com]

**Figure 5:**
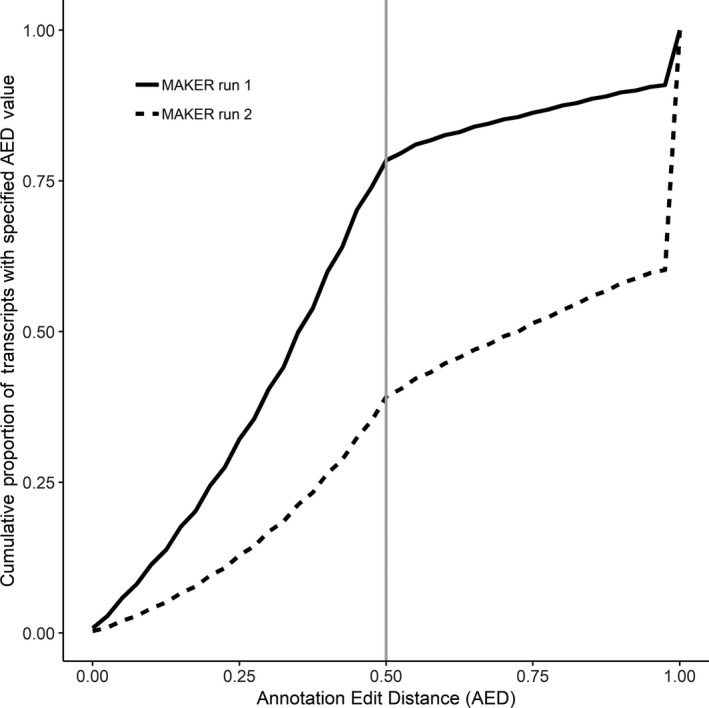
Cumulative proportion of transcripts with specific or lower annotation edit distance (AED) for each MAKER run. MAKER run 1 (solid line) had AED ≤0.50 for 78.4% transcripts, whilst MAKER run 2 (dashed line) had 39.2% transcripts with AED ≤0.50. Grey vertical line indicates AED = 0.50. Note that having a larger proportion of lower AED values indicates a genome annotation that is more congruent with the evidence used during the annotation process

We predicted 22,534 genes that produced 34,024 proteins for the first MAKER run on the CamDro2 assembly, and there were 26,237 genes that produced 38,070 proteins for the second MAKER run on the CamDro2 assembly. There were 7.7% (1,730) of genes without an assigned annotation in the first MAKER run, whilst 21.5% (5,639) were unannotated in the second MAKER run. The Arabian dromedary assembly (NCBI Annotation Release 100) predicted 24,457 genes that produced 26,716 proteins. We assessed if predicted proteins were truncated due to uncorrected indels introduced by PacBio reads by comparing the predicted protein length hit distribution of the CamDro1 assembly (Fitak et al., 2016 predicted protein sequences, Illumina‐only data, red line Figure 4), which should lack introduced indels, to that of the CamDro2 assembly after the first (green line Figure 4) and second MAKER runs (blue line Figure 4). Values near 1.0 are desired and indicate predicted proteins (query) have nearly the same length as proteins in the database (subject). First, predicted proteins from the CamDro1 assembly had 21,259 protein hits against the UniProt/TrEMBL database, and 11,631 (54%) hits were between 0.85 and 1.15 (query sequence length/ subject sequence length; Figure 4). Second, predicted proteins from the CamDro2 assembly for MAKER run 1 had 32,296 protein hits, and 17,267 (53%) were between 0.85 and 1.15 (Figure 4). Third, predicted proteins for MAKER run 2 had 32,415 protein hits, and 11,478 (35%) were between 0.85 and 1.15 (Figure 4). AEDs were overall much higher in the second versus the first MAKER run (Figure 5). For example, MAKER run 1 had AED values ≤ 0.5 for 78.4% transcripts versus 39.2% transcripts for MAKER run 2. Lower AED values indicate a better fit to the provided evidence when annotating a genome (Yandell & Ence, 2012). Average sensitivity and specificity at the nucleotide‐, exon‐, and gene‐levels for the Augustus ab initio model used during the first MAKER run were higher than that for the ab initio model used during the second MAKER run (Table S6). Considering the higher proportion of genes with unknown function, higher proportion of truncated proteins, and higher AEDs, we did not choose the predicted genes, proteins, and transcripts from the second MAKER run and instead chose the annotations from the first MAKER run as the final annotation.

After annotating CamDro1 with the same MAKER inputs and settings used for CamDro2 annotation, CamDro2 had 820; 2,722; 35,052; 32,330; and 2,722 more genes, mRNAs, exons, introns, and CDS than CamDro1, respectively. There were similar mean and shortest gene, mRNA, exon, intron, and CDS lengths between CamDro1 and CamDro2 (Table S7); however, longest lengths (except longest intron length) were longer for CamDro2 than CamDro1. The mean mRNAs per gene (2 vs. 1), mean exons per mRNA (11 vs. 10), and mean introns per mRNA (10 vs. 9) were greater in CamDro2 versus CamDro1, respectively.

## 4 DISCUSSION

### 4.1 Genome assembly

We were able to greatly improve the North African dromedary genome assembly by using a combination of chromosome conformation capture sequencing libraries for scaffolding, long reads to fill in gaps, and comparative chromosome mapping to assign super scaffolds to chromosomes. We demonstrate that data from existing Illumina de novo assemblies can be combined with the before‐mentioned techniques to produce high‐quality reference genomes.

Other genome assembly projects that began with Illumina short‐ and long‐insert libraries have also taken advantage of chromosome conformation capture and/ or long‐read technologies to improve assemblies. For example, the AllMis1 assembly (American alligator, *Alligator mississippiensis*) was assembled with Illumina short‐insert libraries and scaffolded with mate‐pair and BAC libraries (Green et al., 2014) and then subsequently improved with Dovetail Chicago libraries resulting in the AllMis2 assembly (Rice et al., 2017). Further, the sooty manageby (*Cercocebus atys*) genome assembly was de novo assembled with Illumina short‐insert and mate‐pair libraries, and gaps were filled in with ~12X coverage of PacBio RS I and II reads (Palesch et al., 2018).

Our assembly strategy to improve CamDro1 differed from that followed by other groups. Conventionally, researchers generate sufficient PacBio sequencing coverage to improve the scaffold N50 length, polish the assembly with Illumina short reads, and further use data from Dovetail Genomics Chicago and Hi‐C libraries to extend scaffolds to the chromosomal level. In contrast, this study scaffolded the CamDro1 Illumina‐only assembly using Dovetail Chicago data, improved the Chicago assembly with Hi‐C data, filled in gaps in the Hi‐C assembly with PacBio reads, and filled in gaps and polished the assembly with Illumina data. The advantage of our assembly strategy is cost, as PacBio long reads are expensive. For example, 50× PacBio Sequel coverage for a de novo PacBio dromedary assembly would cost approximately 35,000 euros at the time of writing. This is approximately five times more than the ~ 11x coverage that we could afford. For this reason, we did not follow the conventional assembly improvement method because our method was much cheaper to use PacBio Sequel reads to fill in gaps. Alternatively, we could have used a hybrid assembler such as masurca (Zimin et al., 2017) to de novo assemble the PacBio and Illumina reads simultaneously, but scaffolding CamDro1 was already completed before we performed PacBio sequencing. We note that, despite the cost constraints, our strategy has significantly improved upon the previous genome assembly.

We found strong conservation of chromosomal arrangement between the CamDro2 and Alpaca assemblies. Future comparisons with additional assemblies, can determine if the inversions and repeats identified in these genomes should be corrected or if they represent unique structural variation of the sequenced individuals. Further improvements (i.e., assembly iterations) to CamDro2 should focus on possible inversions in chromosome 9, 16, 30 and 35.

### 4.2 Genome annotation

There were more and longer annotation features and also more mRNAs per gene and exons and introns per mRNA in CamDro2 versus CamDro1 suggesting that CamDro2 has both improved assembly and annotation statistics relative to CamDro1. Details regarding our Augustus model choice are discussed in the Supplements (Appendix S1: Discussion).

## 5 CONCLUSION

The CamDro2 reference should be of great value to evolutionary biologists and the camelid genetics community, especially researchers interested mammalian comparative genomics and in designing RNA‐Seq and GWAS experiments. In particular, the large scaffolds identified in CamDro2 will be useful in SNP imputation if hundreds of dromedaries are sequenced at low coverage using programs such as STITCH (Davies et al., 2016).

## AUTHOR CONTRIBUTION

J.P.E. performed bioinformatic analyses and wrote the manuscript. M.F.R. provided dromedary RNA‐Seq reads and revised the manuscript. P.L.P., A.P.A, N.A.S, and W.E.J extracted DNA, provided sequences for comparative chromosome mapping, and revised the manuscript. P.H. and D.M. revised the manuscript. J.C. provided computational resources and revised the manuscript. P.A.B. conceived and managed the project and revised the manuscript.

## DATA ACCESSIBILITY

This Whole Genome Shotgun project has been deposited at DDBJ/ENA/GenBank under the accession JWIN00000000. The version described in this paper is version JWIN02000000. Raw PacBio Sequel subread sequences are available from NCBI Sequence Read Archive (accession SRP050586). Read alignments (Dovetail Chicago and Dovetail Hi‐C reads mapped to original North African dromedary genome assembly, CamDro1) are available upon request. Dromedary RNA‐Seq sequences are from Alim et al., 2019; NCBI BioProject PRJNA543338. CamDro2 gene annotations, predicted mRNA and proteins, assembly for gene annotations, CamDro1 assembly improved by Dovetail Genomics Chicago and Hi‐C libraries, and RH *V. pacos* probe sequences are available from Dryad (https://doi.org/10.5061/dryad.6rp36b6). Example scripts and code for analyses are available from Online Appendix S1: Methods S1 and also the Dryad repository. Note that annotations hosted by NCBI will differ from annotations on Dryad.
