# Supplementary material for: Improving Illumina assemblies with Hi‐C and long reads: An example with the North African dromedary
Source: Mol Ecol Resour. 2019 May 17;19(4):1015–26. doi: 10.1111/1755-0998.13020 (PMC6618069; doi:10.1111/1755-0998.13020)
Supplement: Supplementary file 1 [file MEN-19-1015-s001.docx]

**Supplemental Information for:**

**Improving Illumina assemblies with Hi-C and long reads: an example with the North African dromedary**

Jean P. Elbers, Mark F. Rogers, Polina L. Perelman, Anastasia A. Proskuryakova, Natalia A. Serdyukova, Warren E. Johnson, Petr Horin, Jukka Corander, David Murphy, Pamela A. Burger

**Table of Contents:**

| **Supplemental Tables** | Page 2 |
| --- | --- |
| **Supplemental Figures** | Page 12 |
| **Supplemental Methods** | Page 22 |
| **Supplemental Discussion** | Page 83 |
| **References** | Page 84 |

**Supplemental Tables**

**Table S1.** Percentage and number of the 4,326 National Center for Biotechnology Information (NCBI) eukaryotic genome assemblies assembled using a particular sequencing technology according to available assembly reports accessed on 21 February 2018. Many genomes were assembled with more than one sequencing technology (ex: Illumina plus Sanger), so the total for the column “Number” is actually 5,090 and not 4,326.

| Technology | Percentage | Number |
| --- | --- | --- |
| Illumina | 82.27 | 3,559 |
| 454 | 15.07 | 652 |
| PacBio | 9.15 | 396 |
| Sanger | 7.72 | 334 |
| Ion Torrent PGM or Proton | 1.64 | 71 |
| SOLiD | 1.09 | 47 |
| Oxford Nanopore | 0.28 | 12 |
| 10x Genomics | 0.21 | 9 |
| Bionano | 0.12 | 5 |
| Dovetail Chicago | 0.07 | 3 |
| Dovetail Hi-C | 0.02 | 1 |
| BGI | 0.02 | 1 |

**Table S2.** Assembly statistics for different stages of the North African dromedary genome assembly. BUSCOs: Benchmarking Universal Single-Copy Orthologs [(Simão *et al.*](https://paperpile.com/c/Heq3cT/e3FND), [2015)](https://paperpile.com/c/Heq3cT/e3FND) are mammalian BUSCOs from OrthoDB v. 9.1 genes [(Zdobnov *et al.*](https://paperpile.com/c/Heq3cT/cCJ8O), [2017)](https://paperpile.com/c/Heq3cT/cCJ8O).

|  | Assembly Step | | | | | |
| --- | --- | --- | --- | --- | --- | --- |
|  | 1 | 2 | 3 | 4 | 5 | 6 |
|  | Original North African dromedary genome assembly (CamDro1) | Assembly improved by Dovetail Genomics Chicago and Hi-C libraries | PBJelly to fill in gaps with 11x PacBio reads | Pilon error correct assembly with Illumina short-insert reads | ABYSS Sealer to fill in gaps using Illumina short-insert reads | Pilon error correct round two and also fill in gaps (CamDro2) |
| Single-copy BUSCOs | 3820 | 3811 | 3837 | 3846 | 3851 | 3851 |
| Duplicated BUSCOs | 22 | 26 | 25 | 25 | 25 | 24 |
| Fragmented BUSCOs | 164 | 166 | 146 | 136 | 132 | 133 |
| Missing BUSCOs | 98 | 101 | 96 | 97 | 96 | 96 |
| Proportion of complete BUSCOs | 0.936 | 0.935 | 0.941 | 0.943 | 0.944 | 0.944 |
| Number of scaffolds | 35752 | 24424 | 23439 | 23439 | 23439 | 23439 |
| Total size of scaffolds | 2055063633 | 2066439633 | 2160576013 | 2160833958 | 2154641726 | 2154386959 |
| Longest scaffold | 9719801 | 122948714 | 125000324 | 125008579 | 124995640 | 124992380 |
| Shortest scaffold | 500 | 500 | 443 | 443 | 443 | 281 |
| N50 scaffold length | 1482444 | 73028501 | 75024752 | 75031200 | 75020655 | 75021453 |
| L50 scaffold count | 393 | 11 | 11 | 11 | 11 | 11 |
| N90 scaffold length | 260185 | 24048433 | 24836145 | 24839415 | 24927887 | 24922612 |
| L90 scaffold count | 1592 | 31 | 32 | 32 | 31 | 31 |
| Number of contigs^†^ | 133158 | 133206 | 57943 | 57943 | 47900 | 45969 |
| Longest contig | 413938 | 413938 | 8295500 | 8296053 | 8296005 | 9491684 |
| Shortest contig | 64 | 6 | 64 | 64 | 64 | 48 |
| N50 contig length | 50278 | 50229 | 1043546 | 1043585 | 1166585 | 1333231 |
| L50 contig count | 11378 | 11389 | 544 | 544 | 486 | 423 |
| N90 contig length | 11508 | 11505 | 127221 | 127225 | 152341 | 177667 |
| L90 contig count | 42697 | 42725 | 2548 | 2548 | 2237 | 1944 |
| Number of gaps (>=25bp) | 97406 | 108781 | 34504 | 34504 | 24461 | 22530 |
| Length of gaps (>=25bp) | 53035436 | 64411442 | 22348368 | 22348368 | 20900328 | 20341506 |

^†^Using minimum gap length of 25 bp

**Table S3.** Pacific Biosciences subread sequencing statistics from five PacBio Sequel SMRT Cells.

| SMRT Cell | No. Reads | No. Bases | Mean Length | Median Length | Maximum Length^†^ |
| --- | --- | --- | --- | --- | --- |
| 0 | 376,277 | 5,011,977,624 | 13,319 | 9,464 | 78,273 |
| 1 | 367,398 | 5,243,466,716 | 14,271 | 10,225 | 80,274 |
| 2 | 404,782 | 5,638,996,814 | 13,930 | 10,039 | 106,689 |
| 3 | 298,130 | 4,288,235,741 | 14,383 | 10,402 | 99,366 |
| 4 | 327,128 | 4,649,627,707 | 14,213 | 10,150 | 75,656 |
| Mean | 354,743 | 4,966,460,920 | 14,023 | 10,056 | 88,052 |
| Total | 1,773,715 | 24,832,304,602 |  |  |  |
| Estimated Genome Size^‡^ | | 2,270,000,000 |  |  |  |
| Estimated Coverage | | 10.94 |  |  |  |

^†^All five PacBio Sequel SMRT Cells had a minimum subread length of 50 bases.

^‡^Fitak *et al.*, [(2016)](https://paperpile.com/c/Heq3cT/NAIKs/?noauthor=1)

**Table S4.** *Vicuna (Lama) pacos* RH probes (W.E.J. unpublished data; Avila *et al.*, 2014) mapped with blastn+ (Altschul, 1990) using an E value ≤ 1e-30 and max hsps 1 to CamDro2 assembly scaffolds. Only the highest E value was kept for each RH probe.

| CamDro2 Chromosome | RH marker hits (percent) to this CamDro2 Chromosome | RH marker hits to all CamDro2 scaffolds | Total RH markers tested |
| --- | --- | --- | --- |
| 1 | 170 (98.8) | 172 | 270 |
| 2 | 187 (95.9) | 195 | 270 |
| 3 | 180 (97.8) | 184 | 270 |
| 4 | 109 (85.8) | 127 | 192 |
| 5 | 149 (100) | 149 | 224 |
| 6 | 172 (99.4) | 173 | 508 |
| 7 | 130 (99.2) | 131 | 182 |
| 8 | 118 (99.2) | 119 | 161 |
| 9 | 101 (99) | 102 | 175 |
| 10 | 95 (96.9) | 98 | 175 |
| 11 | 112 (98.2) | 114 | 166 |
| 12 | 111 (97.4) | 114 | 155 |
| 13 | 93 (98.9) | 94 | 166 |
| 14 | 83 (100) | 83 | 123 |
| 15 | 70 (100) | 70 | 174 |
| 16 | 79 (87.8) | 90 | 163 |
| 17 | 111 (99.1) | 112 | 232 |
| 18 | 61 (81.3) | 75 | 151 |
| 19 | 50 (96.2) | 52 | 85 |
| 20 | 55 (100) | 55 | 100 |
| 21 | 42 (95.5) | 44 | 62 |
| 22 | 33 (97.1) | 34 | 56 |
| 23 | 48 (85.7) | 56 | 81 |
| 24 | 34 (100) | 34 | 49 |
| 25 | 66 (98.5) | 67 | 91 |
| 26 | 36 (100) | 36 | 55 |
| 27 | 29 (100) | 29 | 44 |
| 28 | 14 (100) | 14 | 23 |
| 29 | 37 (94.9) | 39 | 49 |
| 30 | 34 (100) | 34 | 84 |
| 31 | 23 (100) | 23 | 31 |
| 32 | 38 (100) | 38 | 54 |
| 33 | 30 (100) | 30 | 43 |
| 34 | 34 (100) | 34 | 45 |
| 35 | 30 (100) | 30 | 45 |
| 36 | 3 (60) | 5 | 5 |
| X | 149 (100) | 149 | 222 |

**Table S5.** *Vicuna pacos* Hi-C assembly (<https://www.dnazoo.org/assemblies/Vicugna_pacos>; hereafter Alpaca assembly) scaffolds assigned to chromosomes 1–36 and X with alpaca RH probes sets from chromosomes 1–36 and X (W.E.J. unpublished data; Avila *et al.,* 2014). We inferred chromosome numbers by blastn v. 2.2.31+ [(Altschul 1990)](https://paperpile.com/c/Heq3cT/fjTlp) using an E value ≤ 1e-30 and max hsps 1 of RH probes against Alpaca scaffolds, keeping only the highest E value hit for each RH probe, and assigning chromosome number based on the Alpaca scaffold with the most blast hits for each alpaca chromosome RH probe set. We could not assign the Y chromosome as we do not have an alpaca RH probe chromosome set for the Y chromosome.

| Chromosome | Alapca scaffold |
| --- | --- |
| 1 | HiC_scaffold_26 |
| 2 | HiC_scaffold_27 |
| 3 | HiC_scaffold_28 |
| 4 | HiC_scaffold_19 |
| 5 | HiC_scaffold_31 |
| 6 | HiC_scaffold_35 |
| 7 | HiC_scaffold_18 |
| 8 | HiC_scaffold_12 |
| 9 | HiC_scaffold_8 |
| 10 | HiC_scaffold_14 |
| 11 | HiC_scaffold_21 |
| 12 | HiC_scaffold_37 |
| 13 | HiC_scaffold_36 |
| 14 | HiC_scaffold_10 |
| 15 | HiC_scaffold_7 |
| 16 | HiC_scaffold_20 |
| 17 | HiC_scaffold_34 |
| 18 | HiC_scaffold_3 |
| 19 | HiC_scaffold_22 |
| 20 | HiC_scaffold_13 |
| 21 | HiC_scaffold_15 |
| 22 | HiC_scaffold_6 |
| 23 | HiC_scaffold_32 |
| 24 | HiC_scaffold_33 |
| 25 | HiC_scaffold_5 |
| 26 | HiC_scaffold_2 |
| 27 | HiC_scaffold_23 |
| 28 | HiC_scaffold_29 |
| 29 | HiC_scaffold_9 |
| 30 | HiC_scaffold_24 |
| 31 | HiC_scaffold_16 |
| 32 | HiC_scaffold_25 |
| 33 | HiC_scaffold_30 |
| 34 | HiC_scaffold_4 |
| 35 | HiC_scaffold_11 |
| 36 | HiC_scaffold_17 |
| X | HiC_scaffold_1 |

**Table S6.** Average sensitivity and specificity (in parentheses) for the Augustus *ab initio* models used during the first and second MAKER runs tested against 75 sets of 250 randomly chosen transcripts with annotation edit distance ≤ 0.25. The *ab initio* model used during the first MAKER run was generated with BUSCO [(Simão *et al.*](https://paperpile.com/c/Heq3cT/e3FND), [2015)](https://paperpile.com/c/Heq3cT/e3FND) searching for Eukaroyota OrthoDB v. 9.1 genes [(Zdobnov *et al*](https://paperpile.com/c/Heq3cT/cCJ8O)., [2017)](https://paperpile.com/c/Heq3cT/cCJ8O). The *ab initio* model used during the second MAKER run was generated with the output of MAKER run 1 using Augustus’s [(Stanke *et al.*](https://paperpile.com/c/Heq3cT/IHv53), [2006)](https://paperpile.com/c/Heq3cT/IHv53) autoAug.pl script.

| MAKER run *ab initio* model | Nucleotide-level | Exon-level | Gene-level |
| --- | --- | --- | --- |
| First | 0.864 (0.900) | 0.727 (0.742) | 0.252 (0.236) |
| Second | 0.811 (0.543) | 0.513 (0.389) | 0.082 (0.046) |

**Table S7.** Genome Annotation Generator (Geib *et al.*, 2018) annotation statistics for CamDro2 and CamDro1 using the same MAKER settings and input files.

|  | CamDro2 | CamDro1 |
| --- | --- | --- |
| Total sequence length | 2154386959 | 2055063633 |
| Number of genes | 22534 | 21714 |
| Number of mRNAs | 34024 | 31302 |
| Number of exons | 363303 | 328251 |
| Number of introns | 329279 | 296949 |
| Number of CDS | 34024 | 31302 |
| Total gene length | 355096872 | 334169256 |
| Total mRNA length | 661558273 | 591211363 |
| Total exon length | 131924395 | 113687878 |
| Total intron length | 530292436 | 478117383 |
| Total CDS length | 53360211 | 48616125 |
| Shortest gene | 99 | 93 |
| Shortest mRNA | 99 | 93 |
| Shortest exon | 3 | 3 |
| Shortest intron | 20 | 20 |
| Shortest CDS | 84 | 93 |
| Longest gene | 227941 | 188022 |
| Longest mRNA | 227941 | 188022 |
| Longest exon | 41498 | 30408 |
| Longest intron | 36591 | 40135 |
| Longest CDS | 68487 | 68061 |
| mean gene length | 15758 | 15390 |
| mean mRNA length | 19444 | 18887 |
| mean exon length | 363 | 346 |
| mean intron length | 1610 | 1610 |
| mean CDS length | 1568 | 1553 |
| % of genome covered by genes | 16.5 | 16.3 |
| % of genome covered by CDS | 2.5 | 2.4 |
| mean mRNAs per gene | 2 | 1 |
| mean exons per mRNA | 11 | 10 |
| mean introns per mRNA | 10 | 9 |

**Supplemental Figures**

**Figure S1.** CamDro2 assembly strategy overview

**
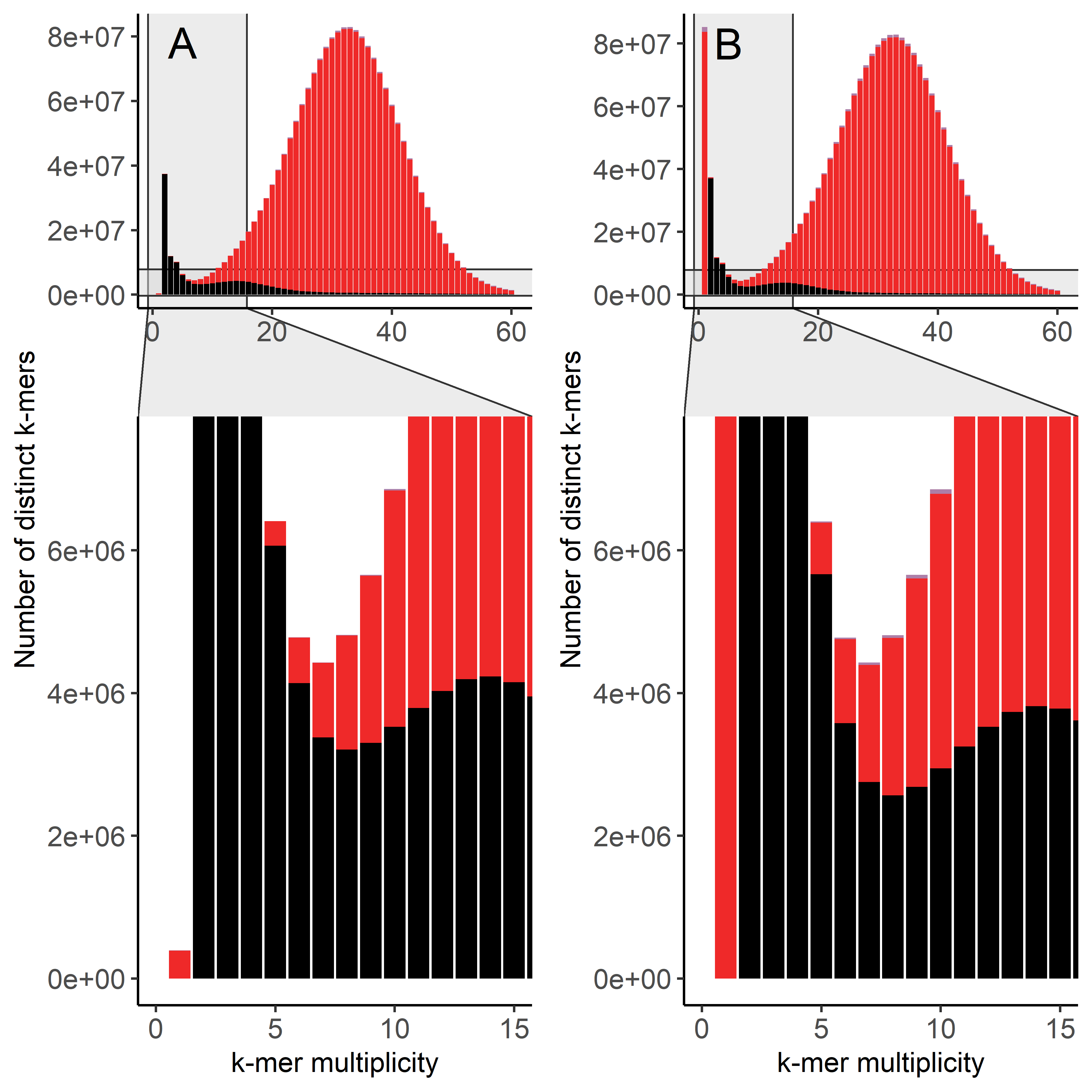
**

**Figure S2.** K-mer analysis toolkit [(KAT; Mapleson *et al.*](https://paperpile.com/c/Heq3cT/oayU/?prefix=KAT%3B%20), [2017)](https://paperpile.com/c/Heq3cT/oayU/?prefix=KAT%3B%20) plots of Illumina short-insert reads (Sequence Read Archive accession: SRR2002493) compared to: (a) the original North African dromedary genome assembly (CamDro1; [Fitak *et al.*](https://paperpile.com/c/Heq3cT/NAIKs/?suffix=%3B%20GenBank%20accession%3A%20GCA_000803125.1), [2016; GenBank accession: GCA_000803125.1)](https://paperpile.com/c/Heq3cT/NAIKs/?suffix=%3B%20GenBank%20accession%3A%20GCA_000803125.1), and (b) the North African dromedary genome assembly after improvement (CamDro2) for 27-mers. Figure key and colors indicate haplotype duplication rates (black for Illumina short-insert sequencing data missing from the assembly, red for 1x haplotype duplication rate, purple for 2x haplotype duplication rate). There was a low proportion of sequence data missing (i.e., black 0x bars) from both CamDro1 and CamDro2 assemblies. Both assemblies were mostly haploid (i.e., red 1x bars) with low heterozygosity (peak at k-mer multiplicity of 15 for black 0x bars). The CamDro2 assembly had a lower proportion of missing sequences than the CamDro1 assembly indicated by less black shading between k-mer multiplicity values 5 and 10 (see panels below (a) and (b) for magnified views), which is replaced by increased red shading at k-mer multiplicity values near 1.

**
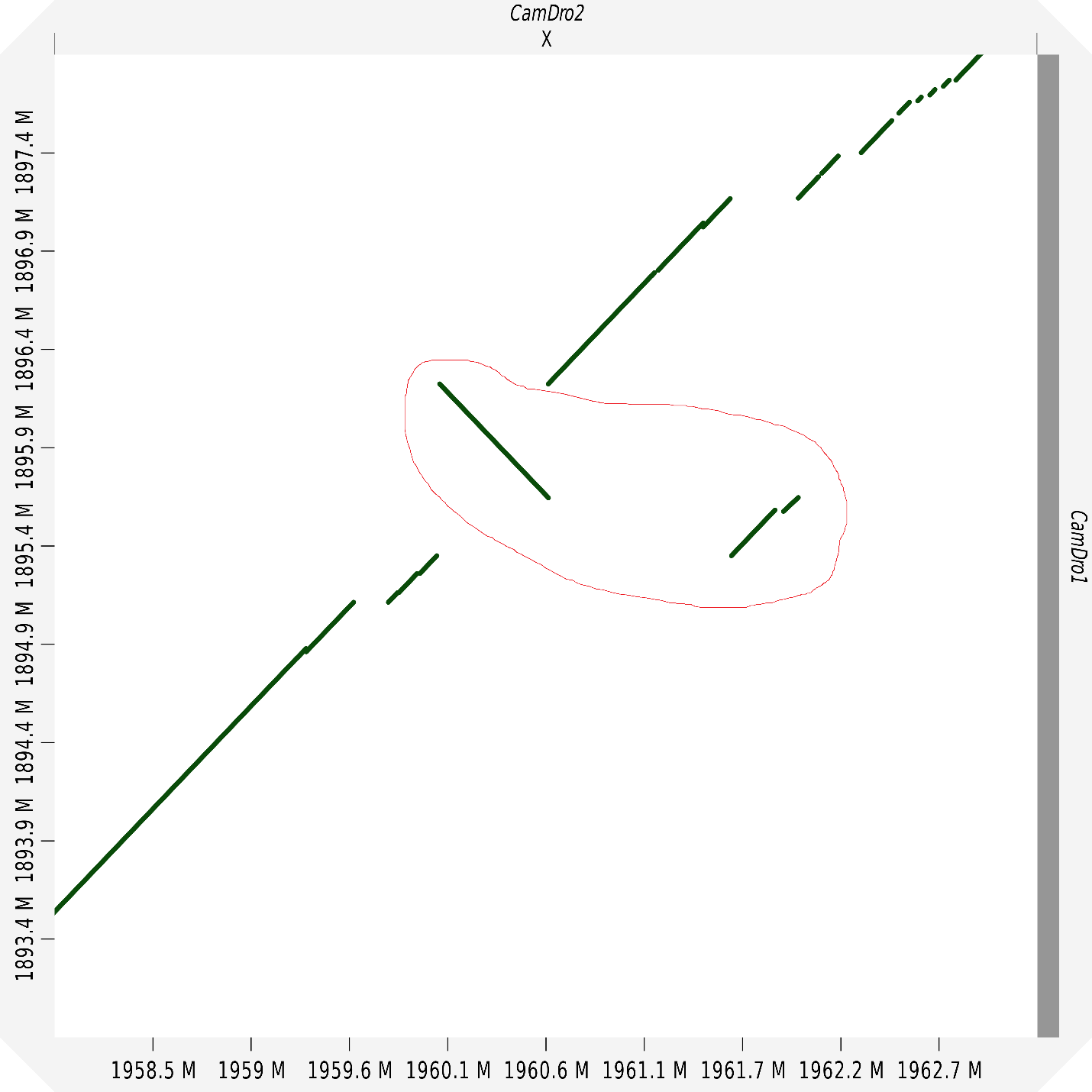
**

**Figure S3.** D-GENIES (Cabanettes & Klopp, 2018) dot plot made with Minimap2 (Li, 2018) whole-genome alignment between CamDro1 and CamDro2 assemblies. Contigs are sorted and matches are filtered out by size using ≤ 0.001 % dot plot width and identity ≤ 0.75. Region shown is a magnified view of CamDro2 chromosome X. Red circled region is CamDro1 scaffold JWIN01032405.1 that is split and is inverted relative to CamDro2 chromosome X.


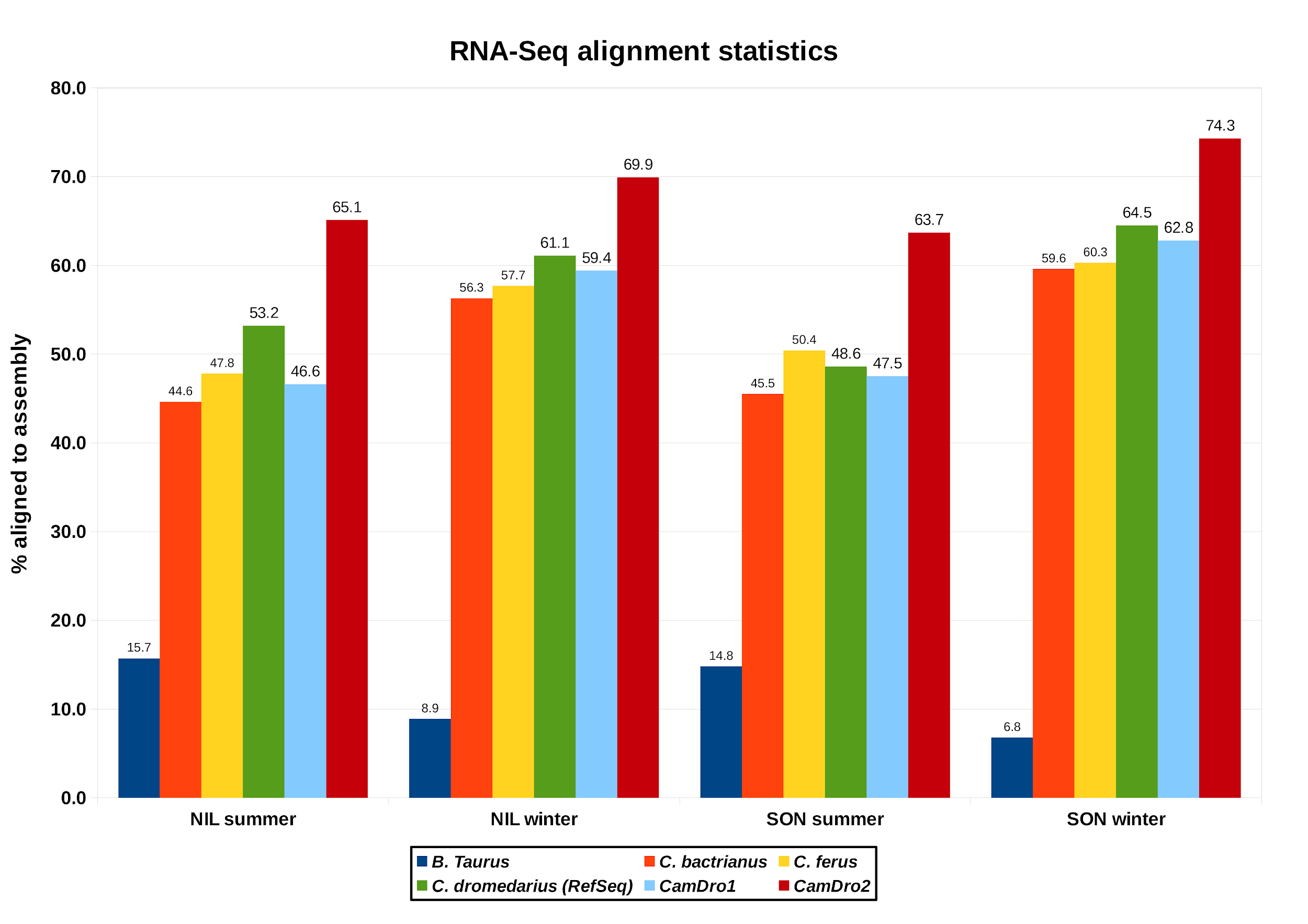
**Figure S4.** Overall alignment rates for RNA-Seq data from (Alim *et al.*, in review). Alignments were made against *Bos taurus* (*B. Taurus -* GCA_000003055.3), *Camelus bactrianus* (*C. bactrianus* - GCA_000767855.1), *Camelus ferus* (*C. ferus* - GCA_000311805.2), *Camelus dromedarius* (*C. dromedarius* (RefSeq) - GCA_000767585.1), *C. dromedarius* (CamDro1 - GCA_000803125.1), and *C. dromedarius* (CamDro2) genome assemblies. The RNA-Seq datasets comprise a 2x2 factorial experiment: summer vs. winter seasons and supraoptic nucleus (SON) vs. neurointermediate lobe (NIL) brain tissues, with n=3 replicates in each class.


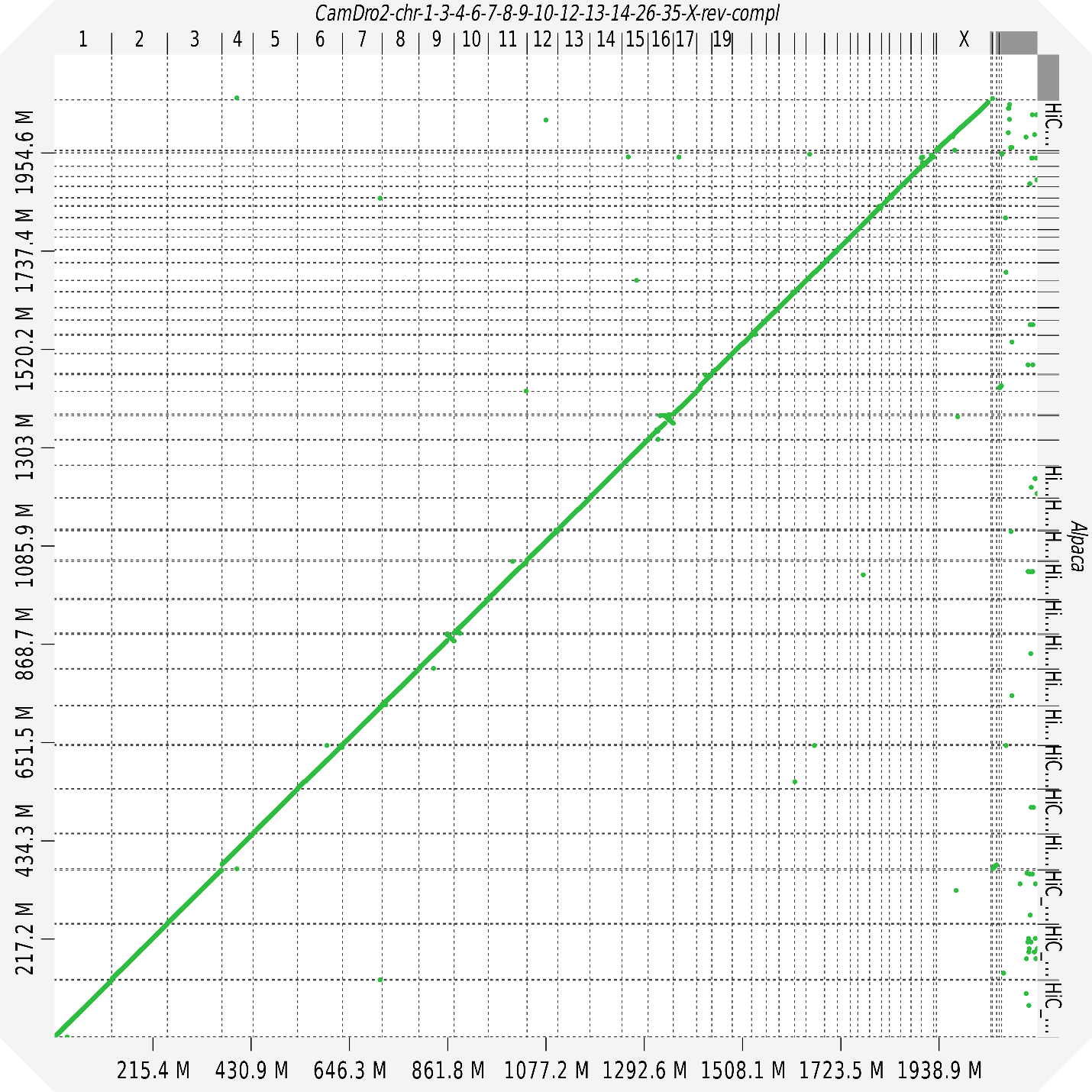


**Figure S5.** D-GENIES (Cabanettes & Klopp, 2018) dot plot made with Minimap2 (Li, 2018) whole-genome alignment between a recently made public *V. pacos* Hi-C assembly (<https://www.dnazoo.org/assemblies/Vicugna_pacos>; hereafter Alpaca assembly) and CamDro2 assemblies. Contigs are sorted and matches are filtered out by size using ≤ 0.001 % dot plot width and identity ≤ 0.5.


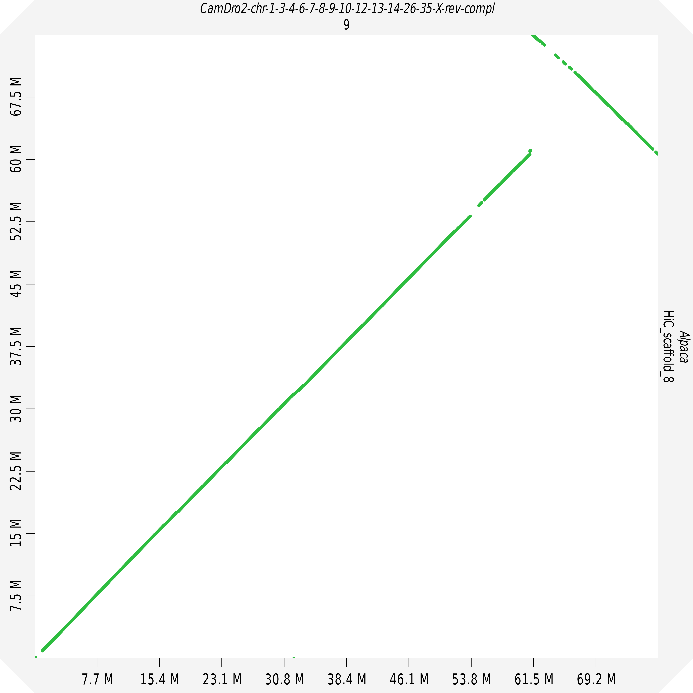


**Figure S6.** Magnified views of chromosome 9 dot plot for whole-genome alignment between CamDro2 and Alpaca. There may be an inversion in one of the assemblies.


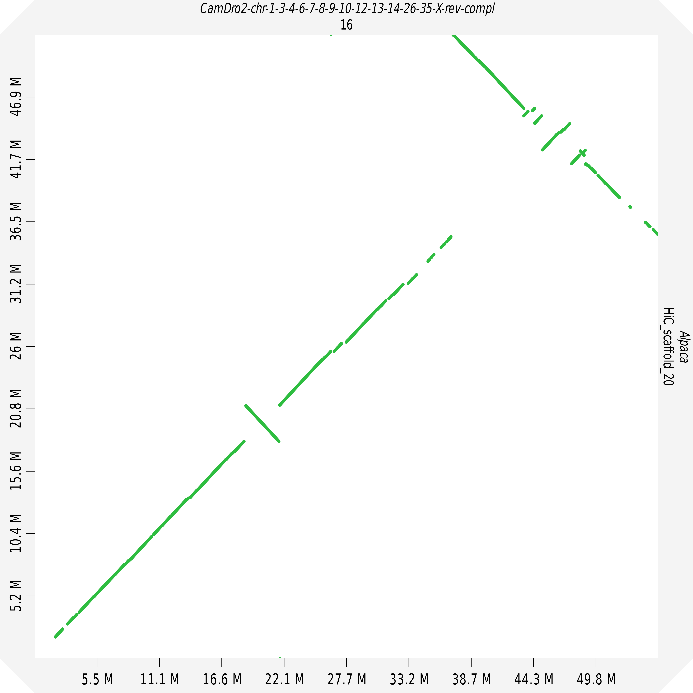


**Figure S7.** Magnified views of chromosome 16 dot plot for whole-genome alignment between CamDro2 and Alpaca. There may be two inversions in one of the assemblies.


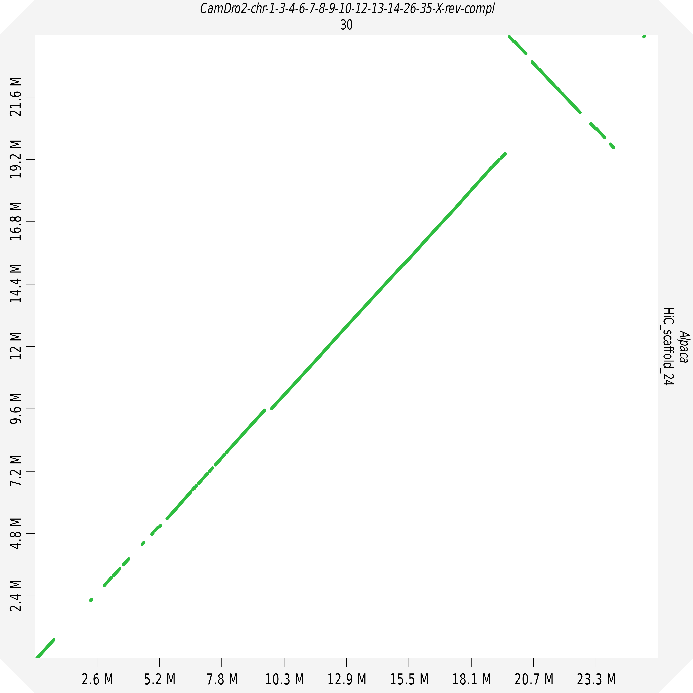


**Figure S8.** Magnified views of chromosome 30 dot plot for whole-genome alignment between CamDro2 and Alpaca. There may be an inversion in one of the assemblies.


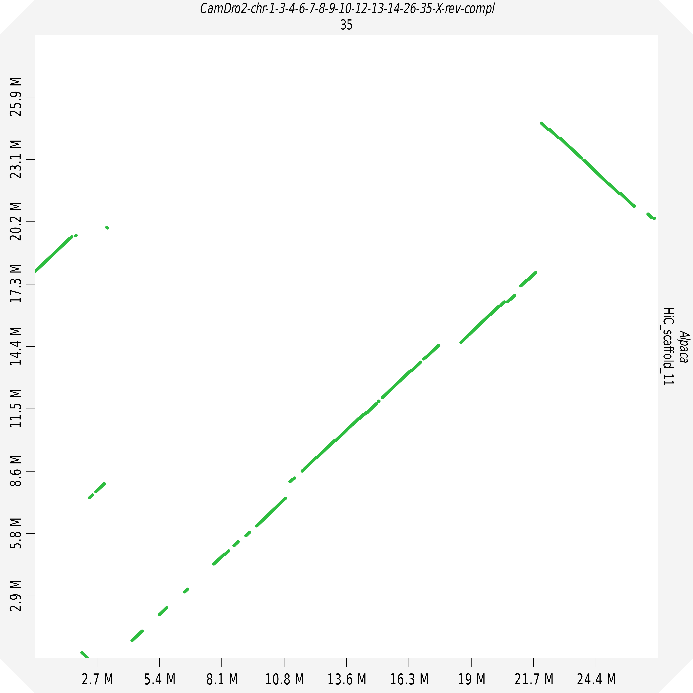


**Figure S9.** Magnified views of chromosome 35 dot plot for whole-genome alignment between CamDro2 and Alpaca. There may be an inversion and some repetive sequences in one of the assemblies.

**Supplemental Methods**

**Methods S1.**

# Analysis steps for Elbers et al. 2019

# Improving Illumina assemblies with Hi-C and long reads: an example with the North African dromedary

#

#########################

# STEP 1 Make directories

#########################

cd /genetics/

mkdir pacbio

chmod a+rwx pacbio

cd pacbio

#########################

# STEP 2 Copy dovetail reference to directory

#########################

wget https://www.dropbox.com/s/w3desejfqk5t7zd/dromedary.fasta.gz?dl=0

unpigz dromedary.fasta.gz

#########################

# STEP 3 Copy PacBio SMRT Cells tar files to directory

#########################

#########################

# STEP 4 Untar the PacBio Results

#########################

tar xzf r54067_20180110_125232_1_A01.tgz

tar xzf r54067_20180112_085457_1_A01.tgz

tar xzf r54067_20180112_085457_2_B01.tgz

tar xzf r54067_20180112_085457_3_C01.tgz

tar xzf r54067_20180112_085457_4_D01.tgz

#########################

# STEP 5 Use bamtools to extract FASTQ from BAM files

#########################

/opt/bamtools/bin/bamtools convert -format fastq -in r54067_20180110_125232/1_A01/*.subreads.bam -out 0.subreads.fastq

/opt/bamtools/bin/bamtools convert -format fastq -in r54067_20180112_085457/1_A01/*.subreads.bam -out 1.subreads.fastq

/opt/bamtools/bin/bamtools convert -format fastq -in r54067_20180112_085457/2_B01/*.subreads.bam -out 2.subreads.fastq

/opt/bamtools/bin/bamtools convert -format fastq -in r54067_20180112_085457/3_C01/*.subreads.bam -out 3.subreads.fastq

/opt/bamtools/bin/bamtools convert -format fastq -in r54067_20180112_085457/4_D01/*.subreads.bam -out 4.subreads.fastq

rm -r r54067_20180110_125232/

rm -r r54067_20180112_085457/

#########################

# STEP 6 Combine the FASTQ files and remove the originals to save space

#########################

## first combine files and delete the originals to save space

cat ?.subreads.fastq > pacbio-dromedary-pbjelly.fastq

rm ?.subreads.fastq

## second add fake quality values (from Q0=! to Q30=> in Sanger encoding) for pbjelly to work

cat pacbio-dromedary-pbjelly.fastq | tr "!" ">" > pacbio-dromedary-pbjelly-fqual.fastq

### third remove fastq file with quality of 0

rm pacbio-dromedary-pbjelly.fastq

#########################

# STEP 7 Create pbjelly-config-assemble.xml

#########################

## use nano to create the file then paste everything between the lines starting with #### and ending with ####

nano pbjelly-config-assemble.xml

####pbjelly-config-assemble.xml####

<jellyProtocol>

<reference>/genetics/pacbio/dromedary.fasta</reference>

<outputDir>/genetics/pacbio/</outputDir>

<cluster>

<command notes="For single node, multi-core machines" >${CMD} ${JOBNAME} 2> ${STDERR} 1> ${STDOUT} &amp;</command>

<nJobs>70</nJobs>

</cluster>

<blasr>-minMatch 8 -minPctIdentity 70 -bestn 1 -nCandidates 10 -maxScore -500 -nproc 1 -noSplitSubreads</blasr>

<input baseDir="/genetics/pacbio/">

<job>pacbio-dromedary-pbjelly-fqual.fastq</job>

</input>

</jellyProtocol>

####pbjelly-config-assemble.xml####

#########################

# STEP 8 Create fake qualities for reference to fill in gaps for

#########################

/opt/PBSuite_15.8.24/bin/fakeQuals.py dromedary.fasta dromedary.qual

#########################

# STEP 9 Summarize the assembly and reads

#########################

## first Summarize the assembly

/opt/PBSuite_15.8.24/bin/summarizeAssembly.py dromedary.fasta > dromedary.fasta.summary

## second Summarize the reads

/opt/PBSuite_15.8.24/bin/readSummary.py pbjelly-config-assemble.xml > pacbio-dromedary-pbjelly-fqual.fastq.summary

#########################

# STEP 10 Setup pbjelly

#########################

## note - this step takes about 1 hour

## note2 - pbjelly steps are submitted in the background, but even though it says step is

## finished, it might not be, you need to see if there are still pbjelly processes running

## for example "ps aux|grep -v "root"|less -S" to see if you still see PBJellySuite or

## blasr or Setup.py or Extraction.py or Support.py or Assemble.py or Out.py

/opt/PBSuite_15.8.24/bin/Jelly.py setup pbjelly-config-assemble.xml

#########################

# STEP 11 Map FASTQ reads to dromedary.fasta reference

#########################

/opt/PBSuite_15.8.24/bin/Jelly.py mapping pbjelly-config-mapping.xml

#########################

# STEP 12 Summarize mapping results

#########################

/opt/PBSuite_15.8.24/bin/Jelly.py support pbjelly-config-assemble.xml -x "--minMapq=50"

#########################

# STEP 13 Extract reads that appear to bridge gaps

#########################

/opt/PBSuite_15.8.24/bin/Jelly.py extraction pbjelly-config-assemble.xml

#########################

# STEP 14 Assemble reads to fill in gaps

#########################

/opt/PBSuite_15.8.24/bin/Jelly.py assembly pbjelly-config-assemble.xml

#########################

# STEP 15 Produce output with gaps filled in

#########################

/opt/PBSuite_15.8.24/bin/Jelly.py output pbjelly-config-assemble.xml

#########################

# STEP 16 Determine the number of gaps closed by PBJelly

#########################

## first determine the number of gaps in the original dovetail dromedary assembly

/opt/PBSuite_15.8.24/bin/summarizeAssembly.py dromedary.fasta > dromedary.fasta.summary

## second determine the number of gaps in the pbjelly assembly where gaps in the dovetail assembly were filled in

/opt/PBSuite_15.8.24/bin/summarizeAssembly.py jelly.out.fasta > jelly.out.fasta.summary

#########################

# STEP 17 Run pilon to correct SNPs,indels, fill in gaps using Illumina data

#########################

## first get rawreads

cd /genetics/pacbio/

wget ftp://ftp-trace.ncbi.nih.gov/sra/sra-instant/reads/ByStudy/sra/SRP/SRP050/SRP050586/SRR2002493/SRR2002493.sra

## second fastqdump

/opt/sratoolkit.2.8.2-1/bin/fastq-dump \

--split-files --gzip SRR2002493.sra

## third unzip illumina reads

unpigz SRR2002493_?.fastq.gz

## fourth interleave the FASTQ files

/opt/bbmap/reformat.sh -Xmx350g threads=75 in1=SRR2002493_1.fastq addslash=t spaceslash=f \

trimreaddescription=t in2=SRR2002493_2.fastq out=SRR2002493_interleaved.fastq 2> interleave-fastq.log

## fifth interleave the FASTQ files

/opt/bbmap/reformat.sh -Xmx350g threads=75 in1=SRR2002493_1.fastq addslash=t spaceslash=f \

trimreaddescription=t in2=SRR2002493_2.fastq out=SRR2002493_interleaved.fastq.gz 2> interleave-fastq.log

## sixth bwa index, bwa mem, sam2bam, samtools sort, samtools index

bwa index -a bwtsw jelly.out.fasta > bwa-index.log 2>&1 &

bwa mem -p -M -t 75 jelly.out.fasta \

SRR2002493_interleaved.fastq.gz 2> bwa.log |\

samtools view -@75 -Sb - |samtools sort -@75 - > waris-ecc-trimmed-reads-mapped-to-jelly.out.fasta.bam &

samtools index waris-ecc-trimmed-reads-mapped-to-jelly.out.fasta.bam

## seventh create target files for pilon

### a get fasta-splitter.pl

cd /genetics/pabio/

wget http://kirill-kryukov.com/study/tools/fasta-splitter/files/fasta-splitter-0.2.6.zip

unzip fasta-splitter-0.2.6.zip

### b get the contig lengths with samtools faidx

samtools faidx jelly.out.fasta

### c split with fasta-splitter.pl

perl fasta-splitter.pl --part-size 100000000 jelly.out.fasta --out-dir jelly-split/

cd jelly-split/

### move Contig19 and Contig518 to different targetlist

/opt/seqtk/seqtk seq -l0 jelly.out.part-19.fasta | paste - - |grep -Pv ">Contig19\t|>Contig518\t" |tr '\t' '\n' |/opt/seqtk/seqtk seq -l60 > tmp1

/opt/seqtk/seqtk seq -l0 jelly.out.part-19.fasta | paste - - |grep -P ">Contig19\t|>Contig518\t" |tr '\t' '\n' |/opt/seqtk/seqtk seq -l60 > tmp2 && mv tmp2 jelly.out.part-26.fasta

mv tmp1 jelly.out.part-19.fasta

### d create a sequence from 01,02,...,25

seq -w 1 26 > samples

### e get the contig names

while read i;do grep ">" jelly.out.part-${i}.fasta|perl -pe "s/>//g" > targetlist${i}; done < samples

### f get the contig lengths

cut -f 1-2 ../jelly.out.fasta.fai > jelly.chromosome.lengths.txt

### h get the names of the contigs that are in split files where there are less than 10 contigs

wc -l targetlist??|perl -pe "s/( )+/\t/g"|\

awk '$1<10'|cut -f 3 |perl -pe "s/targetlist//g" > samples-to-split

### h write script to make target lists for pilon

### essence of script to generate a file like (assuming Contig0 has 29 bases):

### Contig0:1-10

### Contig0:11-20

### Contig0:21-29

###

### The script below breaks up the task into 75 pieces

#####make-target-list.sh#####

#! /bin/bash

while read i

do

lines="$(wc -l targetlist${i}|cut -d " " -f 1)"

while read line;

do

chrlength="$(grep -P "^$line\t" jelly.chromosome.lengths.txt | cut -f 2)"

numberofsplits="$(perl -w -e "use POSIX; print ceil(75/$lines)")"

numberofsplits2=$(($numberofsplits - 1))

splitlength="$(perl -w -e "use POSIX; print ceil($chrlength/$numberofsplits)")"

splits=1

while [[ $splits -lt $numberofsplits ]]

do

if [[ $splits -eq 1 ]]

then

splitlength3="$(($splitlength + 1))"

stopbase=$(($splitlength * ($splits + 1)))

echo "${line}:1-${splitlength}" >> targetlist-${i}

echo "${line}:${splitlength3}-${stopbase}" >> targetlist-${i}

((splits++))

elif [[ $splits -eq $numberofsplits2 ]]

then

startbase=$((($splitlength * ($splits)) + 1))

echo "${line}:${startbase}-${chrlength}" >> targetlist-${i}

((splits++))

else

stopbase=$(($splitlength * ($splits + 1)))

startbase=$((($splitlength * $splits) + 1))

echo "${line}:${startbase}-${stopbase}" >> targetlist-${i}

((splits++))

fi

done

done < targetlist${i}

done < samples-to-split

#####make-target-list.sh#####

### i execute script

bash make-target-list.sh

### j rename targetlist-01 to targetlist01

while read i;do

mv -f targetlist-${i} targetlist${i}

done < samples-to-split

### k make a list of the targetlists

while read i;do

ls targetlist${i} >> targetlists

done < samples

### l run pilon (takes about 1 day)

cd /genetics/pacbio/jelly-split/

while read i;do

time java -Xmx350g -jar /opt/pilon/pilon-1.22.jar \

--genome ../jelly.out.fasta \

--frags ../waris-ecc-trimmed-reads-mapped-to-jelly.out.fasta.bam \

--output ${i}-pilon \

--targets ${i} \

--diploid \

--changes \

--fix bases --threads 75 > ${i}-pilon.log 2>&1

done < targetlists &

### n combine output files

#### i get rid of extra headers in each output file

while read i;do

/opt/seqtk/seqtk seq -l0 targetlist${i}-pilon.fasta | awk '!seen[$1]++'|/opt/seqtk/seqtk seq -l80 > targetlist${i}-pilon.fasta2

done < samples

#### ii combine the output files

cat $(find ./ -name "targetlist*-pilon.fasta2" | sort -V) > pilon.fasta

#### iii double check the output, commands below should give the same number

grep -P "^>" pilon.fasta|wc -l && grep ">" pilon.fasta|wc -l

#### iv change output file name and remove "_pilon" from end of contig/scaffold names

cp pilon.fasta dromedary.pbjelly.pilon.fasta

perl -pi -e "s/_pilon//g" dromedary.pbjelly.pilon.fasta

#### v how many SNPs did Pilon correct?

less *-pilon.log |grep "Corrected"|grep -Po "\d+ snps"|cut -f 1 -d " "|awk '{sum+=$1} END {print sum}'

#359,441

#### vi how many short indels did Pilon correct?

less *-pilon.log |grep "Corrected"| grep -Po "\d+ small"|cut -f 1 -d " " |awk '{sum+=$1} END {print sum}'

#564,275

#### vi how many bases do these short indels account for?

less *-pilon.log |grep "Corrected"| grep -Po "totaling \d+"|cut -f 2 -d " " |awk '{sum+=$1} END {print sum}'

#757,963

#########################

# STEP 18 Run ABYSS Sealer

#########################

## run Abyss sealer 2.0.2 (takes about 2 days)

cd /genetics/pacbio/jelly-split/

/opt/abyss/bin/abyss-sealer -F 1000 -b40g -j 75 -k90 -k80 -k70 -k60 -k50 -k40 \

-k30 -k20 -S dromedary.pbjelly.pilon.fasta -o dromedary.pbjelly.pilon.abyss.sealer1000 -v \

../SRR2002493_interleaved.fastq.gz > dromedary.pbjelly.pilon.abyss.sealer1000.log 2>&1

##

cp dromedary.pbjelly.pilon.abyss.sealer1000_scaffold.fa ../dromedary.pbjelly.pilon.abyss.fasta

#########################

# STEP 19 run pilon again (but also fill in gaps this time)

#########################

### a get the contig lengths with samtools faidx

samtools faidx dromedary.pbjelly.pilon.abyss.fasta

### b split with fasta-splitter.pl

perl fasta-splitter.pl --part-size 40000000 dromedary.pbjelly.pilon.abyss.fasta --out-dir pilon-split/

cd pilon-split/

### c create a sequence from 01,02,...,39

seq -w 1 39 > samples

### d get the contig names

while read i;do grep ">" dromedary.pbjelly.pilon.abyss.part-${i}.fasta|perl -pe "s/>//g" > targetlist${i}; done < samples

### e get the contig lengths

cut -f 1-2 ../dromedary.pbjelly.pilon.abyss.fasta.fai > pilon.chromosome.lengths.txt

### f get the names of the contigs that are in split files where there are less than 10 contigs

wc -l targetlist??|perl -pe "s/( )+/\t/g"|\

awk '$1<10'|cut -f 3 |perl -pe "s/targetlist//g" > samples-to-split

perl -pi -e "s/(Contig\d+)\s+/\1\n/" targetlist??

### g write script to make target lists for pilon

### essence of script to generate a file like (assuming Contig0 has 29 bases):

### Contig0:1-10

### Contig0:11-20

### Contig0:21-29

###

### The script below breaks up the task into 75 pieces

#! /bin/bash

while read i

do

lines="$(wc -l targetlist${i}|cut -d " " -f 1)"

while read line;

do

chrlength="$(grep -P "^$line\t" pilon.chromosome.lengths.txt | cut -f 2)"

numberofsplits="$(perl -w -e "use POSIX; print ceil(75/$lines)")"

numberofsplits2=$(($numberofsplits - 1))

splitlength="$(perl -w -e "use POSIX; print ceil($chrlength/$numberofsplits)")"

splits=1

while [[ $splits -lt $numberofsplits ]]

do

if [[ $splits -eq 1 ]]

then

splitlength3="$(($splitlength + 1))"

stopbase=$(($splitlength * ($splits + 1)))

echo "${line}:1-${splitlength}" >> targetlist-${i}

echo "${line}:${splitlength3}-${stopbase}" >> targetlist-${i}

((splits++))

elif [[ $splits -eq $numberofsplits2 ]]

then

startbase=$((($splitlength * ($splits)) + 1))

echo "${line}:${startbase}-${chrlength}" >> targetlist-${i}

((splits++))

else

stopbase=$(($splitlength * ($splits + 1)))

startbase=$((($splitlength * $splits) + 1))

echo "${line}:${startbase}-${stopbase}" >> targetlist-${i}

((splits++))

fi

done

done < targetlist${i}

done < samples-to-split

### h rename targetlist-01 to targetlist01

while read i;do

mv -f targetlist-${i} targetlist${i}

done < samples-to-split

### i make a list of the targetlists

while read i;do

ls targetlist${i} >> targetlists

done < samples

### j bwa index, bwa mem, sam2bam, samtools sort, samtools index

cd /genetics/pacbio/

bwa index -a bwtsw dromedary.pbjelly.pilon.abyss.fasta > bwa-index.log 2>&1

bwa mem -p -M -t 75 dromedary.pbjelly.pilon.abyss.fasta \

SRR2002493_interleaved.fastq.gz 2> bwa.log |\

samtools view -@75 -Sb - |samtools sort -@75 - > waris-ecc-trimmed-reads-mapped-to-dromedary.pbjelly.pilon.abyss.fasta.bam

samtools index waris-ecc-trimmed-reads-mapped-to-dromedary.pbjelly.pilon.abyss.fasta.bam

### k run pilon (takes about 1-2 days)

cd /genetics/pacbio/pilon-split/

while read i;do

java -Xmx350g -jar /opt/pilon/pilon-1.22.jar \

--genome ../dromedary.pbjelly.pilon.abyss.fasta \

--frags ../waris-ecc-trimmed-reads-mapped-to-dromedary.pbjelly.pilon.abyss.fasta.bam \

--output ${i}-pilon \

--targets ${i} \

--diploid \

--changes \

--fix bases,gaps --threads 75 > ${i}-pilon.log 2>&1

done < targetlists &

#### i how many SNPs did Pilon correct?

less *-pilon.log |grep "Corrected"|grep -Po "\d+ snps"|cut -f 1 -d " "|awk '{sum+=$1} END {print sum}'

#125,448

#### ii how many short indels did Pilon correct?

less *-pilon.log |grep "Corrected"| grep -Po "\d+ small"|cut -f 1 -d " " |awk '{sum+=$1} END {print sum}'

#101,228

#### iii how many bases do these short indels account for?

less *-pilon.log |grep "Corrected"| grep -Po "totaling \d+"|cut -f 2 -d " " |awk '{sum+=$1} END {print sum}'

#146,165

### l combine output files

#### i get rid of extra headers in each output file

while read i;do

/opt/seqtk/seqtk seq -l0 targetlist${i}-pilon.fasta | awk '!seen[$1]++'|/opt/seqtk/seqtk seq -l80 > targetlist${i}-pilon.fasta2

done < samples

#### ii combine the output files

cat $(find ./ -name "targetlist*-pilon.fasta2" | sort -V) > pilon.fasta

#### iii double check the output, commands below should give the same number

grep -P "^>" pilon.fasta|wc -l && grep ">" pilon.fasta|wc -l

#### iv fourth change output file name and remove "_pilon" from end of contig/scaffold names

cp pilon.fasta dromedary.pbjelly.pilon.abyss.pilon.fasta

cp dromedary.pbjelly.pilon.abyss.pilon.fasta ..

perl -pi -e "s/_pilon//g" dromedary.pbjelly.pilon.abyss.pilon.fasta

## twelfth determine the number of gaps in the pilon and abyss assemblies

summarizeAssembly.py dromedary.pbjelly.pilon.fasta > dromedary.pbjelly.pilon.fasta.summary

summarizeAssembly.py ../dromedary.pbjelly.pilon.abyss.fasta > ../dromedary.pbjelly.pilon.abyss.fasta.summary

summarizeAssembly.py dromedary.pbjelly.pilon.abyss.pilon.fasta > dromedary.pbjelly.pilon.abyss.pilon.fasta.summary &

## thirteenth extract chromosome 20 from pilon assembly then abyss assembly

samtools faidx dromedary.pbjelly.pilon.abyss.pilon.fasta Contig17 > dromedary-chr20-pbjelly-pilon-abyss-pilon.fasta

## fourteenth determine position of MHC region (bounded by RXRB and TRIM27)

#####RXRB_and_TRIM27_mRNA.fasta#####

>NM_001083640.1 Bos taurus retinoid X receptor beta (RXRB), mRNA

ATGTCTTGGGCTGCGCGCCCGCCCTTCCTCCCCCAGCGGCATGCCGCAGGGCAGTGTGGGCCGGTGGGGG

TGCGAAAAGAAATGCATTGTGGGGTCGCGTCCCGGTGGCGGCGGCGGCGGCCCTGGCTGGATCCCGCGGC

GGCGGCGGCGGCGGCGGCGGCCGGAGGACAGCAGGCCCCGGAGCCGGAGCCGGGGGAGGCTGGACGGGAC

GGGATGGGCGACAGCGGGCGGGACTCCCGGAGCCCAGACAGTTCCTCCCCAAATCCCCTTCCCCAGGGGG

CCGCTCCCCCTTCTCCTCCAGGACCACCCTTGCCCCCTTCAGCAGCTGCGTCCCTTGGAGGTTCTGGGGC

TCCACCACCACCCTCGATGCCACCCCCACCACTGGGCTCCCCCTTCCCAGTTATCAGCTCTTCCATGGGG

TCCCCCGGCCTGCCCCCTCCAGCTCCCCCAGGATTCTCCGGGCCTGTCAGCAGTCCCCAGATTAACTCAA

CAGTGTCGCTCCCTGGGGGTGGGTCTGGCCCCCCTGAAGATGTGAAGCCACCAGTCTTAGGGGTCCGGGG

CCTGCACTGTCCACCCCCTCCAGGTGGCCCTGGGGCTGGCAAACGGCTATGTGCAATCTGCGGGGACCGA

AGCTCAGGCAAACACTACGGGGTTTACAGCTGCGAGGGCTGCAAAGGCTTCTTCAAGCGCACCATCCGTA

AGGACCTGACCTACTCGTGCCGGGACAACAAGGACTGCACGGTGGACAAGCGCCAGCGGAACCGCTGTCA

GTACTGCCGCTACCAGAAGTGCCTGGCTACTGGCATGAAGAGGGAGGCTGTACAGGAGGAGCGTCACGGT

GGGAAAGACAAAGACGGGGATGGGGAGGGTGCTGGGGGAGCCCCCGAGGAGATGCCTGTGGACAGGATCC

TGGAGGCAGAGCTTGCTGTGGAGCAGAAGAGCGACCAGGGCGTGGAGGGTCCCGGGGGGACCGGGGGCAG

CGGCAGCAGCCCAAATGACCCTGTGACCAACATCTGCCAGGCAGCTGACAAACAGCTCTTCACGCTTGTT

GAATGGGCAAAGAGGATCCCCCACTTTTCCTCCTTGCCTCTGGATGACCAGGTCATATTGCTACGGGCAG

GGTGGAACGAGCTGCTCATCGCCTCCTTCTCTCACCGATCCATCGATGTCCGAGACGGCATCCTCCTCGC

CACAGGTCTCCACGTGCACCGCAACTCAGCCCATTCCGCAGGCGTGGGAGCCATCTTCGATAGGGTGCTG

ACAGAGCTAGTGTCCAAAATGCGGGACATGAGGATGGACAAGACAGAACTTGGCTGCCTGCGGGCAATCA

TTCTGTTCAATCCAGATGCCAAGGGCCTCTCCAACCCCAGCGAGGTTGAGGTCCTGCGAGAGAAAGTATA

TGCATCCCTGGAGACCTACTGCAAACAGAAGTACCCTGAGCAACAGGGCCGGTTTGCCAAGCTGCTGCTG

CGTCTTCCTGCTCTCAGGTCCATAGGCCTTAAGTGTCTAGAGCATCTGTTTTTCTTCAAGCTCATCGGCG

ACACCCCCATCGACACCTTCCTCATGGAGATGCTTGAGGCTCCCCACCAACTGGCCTGA

>NM_001075799.1 Bos taurus tripartite motif containing 27 (TRIM27), mRNA

CAGGAGGGAGCGCAGTCGAGTTGGGCTCCGCGCTGGGCTGGGCACCGGGGCCCATGCCCCTTCGCCCCCG

CGGGCCCGCGCCATGGCCTCCGGGAGCGTGGCCGAGTGCTTACAGCAGGAGACAACTTGCCCCGTGTGCC

TGCAGTACTTTGTGGAGCCCATGATGCTCGACTGCGGCCACAACATCTGTTGCGCCTGCCTCGCCCGCTG

CTGGGGCGCGGCGGAGACCAATGTGTCGTGCCCGCAATGCCGGGAGACCTTCCCGCAGCGGCACATGCGG

CCCAACCGGCACCTGGCCAACGTGACCCAGCTGGTGAAGCAGTTGCGCACCGAGCGGCCGTCGGGGCCCG

GAGGCGAGATGGGCGTGTGCGAGAAACACCGCGAGCCCCTGAAGCTGTACTGCGAGGAGGACCAGATGCC

CATCTGCGTGGTGTGCGACCGCTCCCGCGAGCACCGCGGCCACAGCGTGCTGCCGCTCGAGGAGGCGGTG

GAGGGCTTCAAGGAGCAAATCCAGAACCAGCTGGACCACCTAAAAAGAGTGAAAGACTTAAAGAAGAGGC

GAAGGGCACAGGGAGAGCAGGCGCGAGCTGAACTCTTGAGCCTGACCCAGATGGAGAGGGAGAAGATTGT

TTGGGAGTTTGAGCAACTGTATCACTCCTTGAAGGAGCATGAGTATCGCCTTTTGGCCCGTCTTGAGGAG

CTAGACTTGGCCATCTACAACAGTATCAATGGTGCCATCACTCAGTTCTCTTGCAACATCTCCCACCTCA

GCAACCTGATTGCCCAGCTGGAAGAGAAGCAGCAGCAACCCACCAGGGAGCTCCTGCAGGACATCGGGGA

CACATTGAGCAGGGCTGAAAGAATCAGGATTCCGGAACCCTGGATCACACCTCCAGACCTGCAAGAGAAA

ATCCACATTTTTGCTCAGAAGTGTCTGTTCTTGACTGAGAGTCTGAAGCAGTTCACAGAAAAAATGCAGT

CAGATATGGAGAAAATCCAAGAATTGAGAGAGGCCCAGTTATACTCAGTGGATGTGACTCTGGACCCAGA

CACAGCCTACCCCAGCCTGATCCTCTCTGATAACCTGCGGCAAGTGCGGTACAGTTACCTCCAGCAGGAC

CTGCCTGACAACCCTGAGCGGTTCAATCTGTTTCCCTGTGTCTTGGGCTCTCCATGCTTCATCGCTGGGA

GACACTATTGGGAGGTAGAGGTGGGAGATAAAGCCAAGTGGACCATAGGTGTCTGTGAAGACTCAGTGTG

CAGAAAAGGCGGGGTAACCTCGGCCCCCCAGAATGGATTCTGGGCAGTGTCCTTGTGGTATGGGAAAGAA

TACTGGGCTCTCACCTCCCCAATGACTGCCCTCCCCCTGCGGACCCCTCTCCAACGGGTGGGGATTTTCT

TGGACTATGATGCTGGCGAGGTCTCTTTCTACAACGTGACAGAGAGGTGTCACACCTTTACTTTCTCTCA

TGCTACCTTCTGTGGGCCTGTCCGGCCCTACTTCAGCCTGAGTTACTCTGGAGGGAAGAGCGCAGCTCCT

CTGATCATCTGCCCCATGAGTGGCATCGATGGGTTTTCTGGCCATGTTGGGAATCATGGTCATTCCATGG

AGACCTCCCCTTGAGGAGGTGAACTCAGGCCAGAAGGGCTGCTGGCCGTACTCCCACCCCAGGCATGAGG

CATCTTGTTGCCTTGCCACTTCCTGCCCATCACAGCTGGATGTTCTTACTACTTTCCATGCCCTGCAGTG

CAAGACAGGATGTCTGTGTTCTCTGCCATCCCCTCCCTTCCCACGAAAATTGTGAGATGTAATAAGTTAT

TGAGATTGCCCAGAAATAAAAACCAGATGTCCAAAAAAAAAAAAAAAAAAAAAAAAAA

#####RXRB_and_TRIM27_mRNA.fasta#####

### a BLAST

/usr/bin/makeblastdb -dbtype nucl -in dromedary-chr20-pbjelly-pilon-abyss-pilon.fasta

/usr/bin/blastn -db dromedary-chr20-pbjelly-pilon-abyss-pilon.fasta -query RXRB_and_TRIM27_mRNA.fasta -outfmt 6 > RXRB_and_TRIM27_mRNA.fasta.blast

### b locate gaps in chromomsome 20 (assumes mhc region is between bases 18,700,000 to 22,700,000

### based on BLAST result location of RXRB and TRIM27 mRNA

seqkit locate --ignore-case --only-positive-strand --pattern "NNNNNNNNNNNNNNNNNNNNNNNNN+" \

dromedary-chr20-pbjelly-pilon-abyss-pilon.fasta | cut -f 1,5,6 > dromedary-chr20-abyss-location-of-gaps.bed

### c convert coordinates of gaps output into bed file with extra fourth column as length of gaps

awk '{b=$2-1;a=$3-b;print $1,b,$3,a;}' dromedary-chr20-abyss-location-of-gaps.bed |awk '$2>18890000'| \

awk -v OFS="\t" '$2<22440000' > dromedary-abyss-mhc-location-of-gaps.bed

#########################

# STEP 20 Assign Contigs to Chromosomes

#########################

## first make a blastdb

cd /genetics/pacbio/

makeblastdb -dbtype nucl -in dromedary.pbjelly.pilon.abyss.pilon.fasta

## second perform blast, keeping only the first best hit with evalue of at least 1e-30

seq -w 1 35 > rhmarkers/samples

echo "X" >> rhmarkers/samples

while read i;do

## third blast the markers for each chromosome

blastn -num_threads 75 -db dromedary.pbjelly.pilon.abyss.pilon.fasta -query rhmarkers/chr${i}rhmarkers.txt -outfmt 6 \

-max_hsps 1 -evalue 1e-30 > rhmarkers/chr${i}rhmarkers.txt.blast

## fourth count the BLAST hits for contigs/scaffolds

echo ${i} >> contigs-to-chromosomes.txt

cut -f 2 rhmarkers/chr${i}rhmarkers.txt.blast |sort |uniq -c|sort -n |tail -n 1|perl -pe "s/( )+/\t/g" |perl -pe "s/^\t//g" |cut -f 2 >> contigs-to-chromosomes.txt

done < rhmarkers/samples

## fifth modify numbers less than 9

perl -pi -e "s/^0//g" contigs-to-chromosomes.txt

## sixth make a copy of pilon assembly and rename it

/opt/seqtk/seqtk seq -l80 dromedary.pbjelly.pilon.abyss.pilon.fasta > dromedary.pbjelly.pilon.abyss.pilon.chromosomes.fasta

## seventh rename the contigs to chromosome names (takes about 1 hour)

cat contigs-to-chromosomes.txt | while read -r ONE;do

read -r TWO

perl -pi -e "s/>${TWO}\n/>${ONE}\n/" dromedary.pbjelly.pilon.abyss.pilon.chromosomes.fasta

done

## eigth sort the chromosomes and contigs by number (ex: 1,2,3,4,X,Contig200,Contig201),

## then output 60 bases per line, then make all bases uppercase (no soft-masking)

cat dromedary.pbjelly.pilon.abyss.pilon.chromosomes.fasta | /opt/seqtk/seqtk seq -l0 | \

paste - - |grep "Contig" > contigs

cat dromedary.pbjelly.pilon.abyss.pilon.chromosomes.fasta | /opt/seqtk/seqtk seq -l0 | \

paste - - |grep -v "Contig" |grep -v "X" > chromosomes

cat dromedary.pbjelly.pilon.abyss.pilon.chromosomes.fasta | /opt/seqtk/seqtk seq -l0 | \

paste - - |grep "X" > Xchromosome

cat chromosomes |sort -k 1.2 -n > tmp2 && mv tmp2 chromosomes

cat contigs |sort -k 1.8 -n > tmp2 && mv tmp2 contigs

cat chromosomes Xchromosome contigs | tr "\t" "\n" |/opt/seqtk/seqtk seq -l60 -U > tmp2

mv tmp2 dromedary.pbjelly.pilon.abyss.pilon.chromosomes.fasta

cat chromosomes Xchromosome | tr "\t" "\n" |/opt/seqtk/seqtk seq -l60 -U > dromedary.pbjelly.pilon.abyss.pilon.chromosomes.chromosomes.only.fasta

cat contigs | tr "\t" "\n" |/opt/seqtk/seqtk seq -l60 -U > dromedary.pbjelly.pilon.abyss.pilon.chromosomes.unscaffolded.contigs.only.fasta

### how many bases are in chromosomes

samtools faidx dromedary.pbjelly.pilon.abyss.pilon.chromosomes.chromosomes.only.fasta

awk '{sum+=$2} END {print sum}' dromedary.pbjelly.pilon.abyss.pilon.chromosomes.chromosomes.only.fasta.fai

#2046839614

### how many bases are in scaffolds not assigned to chromosomes

samtools faidx dromedary.pbjelly.pilon.abyss.pilon.chromosomes.unscaffolded.contigs.only.fasta

awk '{sum+=$2} END {print sum}' dromedary.pbjelly.pilon.abyss.pilon.chromosomes.unscaffolded.contigs.only.fasta.fai

# 107547345

### what proportion of bases are not assigned to chromosomes

# 107547345/(107547345+2046839614) = 0.04992

## ninth, create k-mer histogram with KAT

cd /genetics/pacbio

/opt/bbmap/reformat.sh -threads=75 in=SRR2002493_interleaved.fastq.gz out=SRR2002493_1.fastq.gz out2=SRR2002493_2.fastq.gz

# for dromedary.pbjelly.pilon.abyss.pilon.chromosomes.fasta

mkdir kat

cd /genetis/pacbio/kat

/opt/KAT/bin/kat comp -h -v -t 75 -H 1000000000 -I 1000000000 -o pe_vs_assembly_dromedary.pbjelly.pilon.abyss.pilon.chromosomes <(pigz -cd ../SRR2002493_?.fastq.gz) \

../dromedary.pbjelly.pilon.abyss.pilon.chromosomes.fasta > pe_vs_assembly_dromedary.pbjelly.pilon.abyss.pilon.chromosomes.log 2>&1

python /opt/KAT/bin/kat_plot_spectra-cn.py --dpi 700 -t "b" -o Fig2b-new-dromedary.pbjelly.pilon.abyss.pilon.chromosomes.fasta.png -m 2 -x 60 -y 91406386 -v pe_vs_assembly_dromedary.pbjelly.pilon.abyss.pilon.chromosomes-main.mx

# for fitak genome assembly

#####Note#####This is CamDro1

cd /genetics/pacbio/

wget ftp://ftp.ncbi.nlm.nih.gov/genomes/all/GCA/000/803/125/GCA_000803125.1_Cdrom64K/GCA_000803125.1_Cdrom64K_genomic.fna.gz

pigz -kd GCA_000803125.1_Cdrom64K_genomic.fna.gz

#####Note#####

cd /genetics/pacbio/kat

/opt/KAT/bin/kat comp -h -v -t 75 -H 1000000000 -I 1000000000 -o pe_vs_assembly_GCA_000803125.1_Cdrom64K_genomic <(pigz -cd ../SRR2002493_?.fastq.gz) \

<(pigz -cd ../GCA_000803125.1_Cdrom64K_genomic.fna.gz) > pe_vs_assembly_GCA_000803125.1_Cdrom64K_genomic.log 2>&1

python /opt/KAT/bin/kat_plot_spectra-cn.py --dpi 700 -t "a" -o Fig2a-new-GCA_000803125.1_Cdrom64K_genomic.fna.png -m 2 -x 60 -y 91406386 -v pe_vs_assembly_GCA_000803125.1_Cdrom64K_genomic-main.mx

convert Fig2b-new-dromedary.pbjelly.pilon.abyss.pilon.chromosomes.fasta.png -crop 1177x657+469+3249 +repage Fig2b-new-dromedary.pbjelly.pilon.abyss.pilon.chromosomes.fasta.zoom.png

convert Fig2a-new-GCA_000803125.1_Cdrom64K_genomic.fna.png -crop 1177x657+469+3249 +repage Fig2a-new-GCA_000803125.1_Cdrom64K_genomic.fna.zoom.png

#########################

# STEP 21

#########################

## calculate assembly staticstics

####################################################assemblathon_stats_no_per_base_percent.pl####################################################

#!/usr/bin/perl

#

# assemblathon_stats.pl

#

# A script to calculate a basic set of metrics from a genome assembly

#

# Author: Keith Bradnam, Genome Center, UC Davis

# This work is licensed under a Creative Commons Attribution-NonCommercial-ShareAlike 3.0 Unported License.

#

# Last updated by: $Author: keith $

# Last updated on: $Date: 2011/10/13 00:07:00 $

# Note: modified by Jean P. Elbers (jean.elbers@gmail.com), so that calculate N90/L90 as well as no per base percentage rates

# tested on Perl version 5.10.1

use strict;

use warnings;

use FAlite;

use Getopt::Long;

use List::Util qw(sum max min);

###############################################

#

# C o m m a n d l i n e o p t i o n s

#

###############################################

my $limit; # limit processing of data to first $limit sequences (for quick testing)

my $graph; # produce some output ready for Excel or R

my $csv; # produce CSV output file of results

my $n_limit; # how many N characters should be used to split scaffolds into contigs

my $genome_size; # estimated or known genome size (will be used for some stats)

GetOptions ("limit=i" => \$limit,

"csv" => \$csv,

"graph" => \$graph,

"n=i" => \$n_limit,

"genome_size=i" => \$genome_size);

# set defaults

$limit = 1000000000 if (!$limit);

$n_limit = 25 if (!$n_limit);

# check we have a suitable input file

my $usage = "Usage: assemblathon_stats.pl <assembly_scaffolds_file>

options:

-limit <int> limit analysis to first <int> sequences (useful for testing)

-csv produce a CSV output file of all results

-graph produce a CSV output file of NG(X) values (NG1 through to NG99), suitable for graphing

-n <int> specify how many consecutive N characters should be used to split scaffolds into contigs

-genome_size <int> estimated or known genome size

";

die "$usage" unless (@ARGV == 1);

my ($file) = @ARGV;

###############################################

#

# S o m e G l o b a l v a r i a b l e s

#

###############################################

my $scaffolded_contigs = 0; # how many contigs that are part of scaffolds (sequences must have $n_limit consecutive Ns)

my $scaffolded_contig_length = 0; # total length of all scaffolded contigs

my $unscaffolded_contigs = 0; # how many 'orphan' contigs, not part of a scaffold

my $unscaffolded_contig_length = 0; # total length of all contigs not part of scaffold

my $w = 60; # formatting width for output

my %data; # data structure to hold all sequence info key is either 'scaffold', 'contig' or intermediate', values are seqs & length arrays

my (@results, @headers); # arrays to store results (for use with -csv option)

# make first loop through file, capture some basic info and add sequences to arrays

process_FASTA($file);

print "\n---------------- Information for assembly \'$file\' ----------------\n\n";

if(defined($genome_size)){

my $mbp_size = sprintf("%.2f", $genome_size / 1000000);

printf "%${w}s %10s\n", "Assumed genome size (Mbp)", $mbp_size;

}

# produce scaffold statistics

sequence_statistics('scaffold');

# produce a couple of intermediate statistics based on scaffolded contigs vs unscaffolded contigs

sequence_statistics('intermediate');

# finish with contig stats

sequence_statistics('contig');

# produce CSV output if required

write_csv($file) if ($csv);

exit(0);

##########################################

#

#

# S U B R O U T I N E S

#

#

##########################################

##########################################

# M A I N loop through FASTA file

##########################################

sub process_FASTA{

my ($seqs) = @_;

my $input;

# if dealing with gzip file, treat differently

if($seqs =~ m/\.gz$/){

open($input, "gunzip -c $seqs |") or die "Can't open a pipe to $seqs\n";

} else{

open($input, "<", "$seqs") or die "Can't open $seqs\n";

}

my $fasta = new FAlite(\*$input);

# want to keep track of various contig + scaffold counts

my $seq_count = 0;

while(my $entry = $fasta->nextEntry){

my $seq = uc($entry->seq);

my $length = length($seq);

$seq_count++;

# everything gets pushed to scaffolds array

push(@{$data{scaffold}{seqs}},$seq);

push(@{$data{scaffold}{lengths}},$length);

# if there are not at least 25 consecutive Ns in the sequence we need to split it into contigs

# otherwise the sequence must be a contig itself and it still needs to be put in @contigs array

if ($seq =~ m/N{$n_limit}/){

# add length to $scaffolded_contig_length

$scaffolded_contig_length += $length;

# loop through all contigs that comprise the scaffold

foreach my $contig (split(/N{25,}/, $seq)){

$scaffolded_contigs++;

my $length = length($contig);

push(@{$data{contig}{seqs}},$contig);

push(@{$data{contig}{lengths}},$length);

}

} else {

# must be here if the scaffold is actually just a contig (or is a scaffold with < 25 Ns)

$unscaffolded_contigs++;

$unscaffolded_contig_length += $length;

push(@{$data{contig}{seqs}},$seq);

push(@{$data{contig}{lengths}},$length);

}

# for testing, just use a few sequences

last if ($seq_count >= $limit);

}

close($input);

}

##########################################

# Calculate basic assembly metrics

##########################################

sub sequence_statistics{

my ($type) = @_;

print "\n";

# need descriptions of each result

my $desc;

# there are just a couple of intermediate level statistics to print

if($type eq 'intermediate'){

my $total_size = sum(@{$data{scaffold}{lengths}});

# now calculate percentage of assembly that is accounted for by scaffolded contigs

my $percent = sprintf("%.1f",($scaffolded_contig_length / $total_size) * 100);

$desc = "Percentage of assembly in scaffolded contigs";

printf "%${w}s %10s\n", $desc, "$percent%";

store_results($desc, $percent) if ($csv);

# now calculate percentage of assembly that is accounted for by unscaffolded contigs

$percent = sprintf("%.1f",($unscaffolded_contig_length / $total_size) * 100);

$desc = "Percentage of assembly in unscaffolded contigs";

printf "%${w}s %10s\n", $desc, "$percent%";

store_results($desc, $percent) if ($csv);

# statistics that describe N regions that join contigs in scaffolds

# get number of breaks

my $contig_count = scalar(@{$data{contig}{lengths}});

my $scaffold_count = scalar(@{$data{scaffold}{lengths}});

my $average_contigs_per_scaffold = sprintf("%.1f",$contig_count / $scaffold_count);

$desc = "Average number of contigs per scaffold";

printf "%${w}s %10s\n", $desc, $average_contigs_per_scaffold;

store_results($desc, $average_contigs_per_scaffold) if ($csv);

# now calculate average length of break between contigs

# just find all runs of Ns in scaffolds (>=25) and calculate average length

my @contig_breaks;

foreach my $scaffold (@{$data{scaffold}{seqs}}){

while($scaffold =~ m/(N{25,})/g){

push(@contig_breaks, length($1));

}

}

# set break size to zero if there are no Ns in scaffolds

my $average_break_length;

if(@contig_breaks == 0){

$average_break_length = 0;

} else{

$average_break_length = sum(@contig_breaks) / @contig_breaks;

}

$desc = "Average length of break (>25 Ns) between contigs in scaffold";

printf "%${w}s %10d\n", $desc, $average_break_length;

store_results($desc, $average_break_length) if ($csv);

return();

}

# n

my $count = scalar(@{$data{$type}{lengths}});

$desc = "Number of ${type}s";

printf "%${w}s %10d\n", $desc, $count;

store_results($desc, $count) if ($csv);

# more contig details (only for contigs)

if ($type eq 'contig'){

$desc = "Number of contigs in scaffolds";

printf "%${w}s %10d\n",$desc, $scaffolded_contigs;

store_results($desc, $scaffolded_contigs) if ($csv);

$desc = "Number of contigs not in scaffolds";

printf "%${w}s %10d\n", $desc,$unscaffolded_contigs;

store_results($desc, $unscaffolded_contigs) if ($csv);

}

# total size of sequences

my $total_size = sum(@{$data{$type}{lengths}});

$desc = "Total size of ${type}s";

printf "%${w}s %10d\n", $desc, $total_size;

store_results($desc, $total_size) if ($csv);

# For scaffold data only, can caluclate the percentage of known genome size

if ($type eq 'scaffold' && defined($genome_size)){

my $percent = sprintf("%.1f",($total_size / $genome_size) * 100);

$desc = "Total scaffold length as percentage of assumed genome size";

printf "%${w}s %10s\n", $desc, "$percent%";

store_results($desc, $percent) if ($csv);

}

# longest and shortest sequences

my $max = max(@{$data{$type}{lengths}});

$desc = "Longest $type";

printf "%${w}s %10d\n", $desc, $max;

store_results($desc, $max) if ($csv);

my $min = min(@{$data{$type}{lengths}});

$desc = "Shortest $type";

printf "%${w}s %10d\n", $desc, $min;

store_results($desc, $min) if ($csv);

# find number of sequences above certain sizes

my %sizes_to_shorthand = (1000 => '1K',

10000 => '10K',

100000 => '100K',

1000000 => '1M',

10000000 => '10M');

foreach my $size qw(1000 10000 100000 1000000 10000000){

my $matches = grep { $_ > $size } @{$data{$type}{lengths}};

my $percent = sprintf("%.1f", ($matches / $count) * 100);

$desc = "Number of ${type}s > $sizes_to_shorthand{$size} nt";

printf "%${w}s %10d %5s%%\n", $desc, $matches, $percent;

store_results($desc, $matches) if ($csv);

$desc = "Percentage of ${type}s > $sizes_to_shorthand{$size} nt";

store_results($desc, $percent) if ($csv); }

# mean sequence size

my $mean = sprintf("%.0f",$total_size / $count);

$desc = "Mean $type size";

printf "%${w}s %10d\n", $desc, $mean;

store_results($desc, $mean) if ($csv);

# median sequence size

my $median = (sort{$a <=> $b} @{$data{$type}{lengths}})[$count/2];

$desc = "Median $type size";

printf "%${w}s %10d\n", $desc, $median;

store_results($desc, $median) if ($csv);

##################################################################################

#

# N50 N90 values

#

# Includes N(x) values, NG(x) (using assumed genome size)

# and L(x) values (number of sequences larger than or equal to N50 or N90 sequence size)

##################################################################################

# keep track of cumulative assembly size (starting from smallest seq)

my $running_total = 0;

# want to store all N50-style values from N1..N100. First target size to pass is N1

my $n_index = 1;

my @n_values;

my $n50_length = 0;

my $n90_length = 0;

my $i = 0;

my $x = $total_size * 0.5;

my $y = $total_size * 0.9;

# start with longest lengths scaffold/contig

foreach my $length (reverse sort{$a <=> $b} @{$data{$type}{lengths}}){

$i++;

$running_total += $length;

# check the current sequence and all sequences shorter than current one

# to see if they exceed the current NX value

while($running_total > int (($n_index / 100) * $total_size)){

if ($n_index == 50){

$n50_length = $length;

$desc = "N50 $type length";

printf "%${w}s %10d\n", $desc, $length;

store_results($desc, $length) if ($csv);

# L50 = number of scaffolds/contigs that are longer than or equal to the N50 size

$desc = "L50 $type count";

printf "%${w}s %10d\n","L50 $type count", $i;

store_results($desc, $i) if ($csv);

}

if ($n_index == 90){

$n90_length = $length;

$desc = "N90 $type length";

printf "%${w}s %10d\n", $desc, $length;

store_results($desc, $length) if ($csv);

# L90 = number of scaffolds/contigs that are longer than or equal to the N90 s$

$desc = "L90 $type count";

printf "%${w}s %10d\n","L90 $type count", $i;

store_results($desc, $i) if ($csv);

}

$n_values[$n_index] = $length;

$n_index++;

}

}

my @ng_values;

# do we have an estimated/known genome size to work with?

if(defined($genome_size)){

my $ng_index = 1;

my $ng50_length = 0;

$running_total = 0;

$i = 0;

foreach my $length (reverse sort{$a <=> $b} @{$data{$type}{lengths}}){

$i++;

$running_total += $length;

# now do the same for NG values, using assumed genome size

while($running_total > int (($ng_index / 100) * $genome_size)){

if ($ng_index == 50){

$ng50_length = $length;

$desc = "NG50 $type length";

printf "%${w}s %10d\n", $desc, $length;

store_results($desc, $length) if ($csv);

$desc = "LG50 $type count";

printf "%${w}s %10d\n", $desc, $i;

store_results($desc, $i) if ($csv);

}

$ng_values[$ng_index] = $length;

$ng_index++;

}

}

my $n50_diff = abs($ng50_length - $n50_length);

$desc = "N50 $type - NG50 $type length difference";

printf "%${w}s %10d\n", $desc, $n50_diff;

store_results($desc, $n50_diff) if ($csv);

}

# add final value to @n_values and @ng_values which will just be the shortest sequence

# $n_values[100] = $min;

# $ng_values[100] = $min;

# anything to dump for graphing?

if($graph){

# create new output file name

my $file_name = $file;

$file_name =~ s/\.gz$//;

$file_name =~ s/\.(fa|fasta)$//;

$file_name .= ".${type}.NG50.csv";

open(my $out, ">", "$file_name") or die "Can't create $file_name\n";

print $out join (',',"Assembly",1..99), "\n";

# make some guesses of what might constitute the unique assembly ID

my $assembly_ID = $file;

($assembly_ID) = $file =~ m/^([A-Z]\d{1,2})_/ if ($file =~ m/^[A-Z]\d{1,2}_/);

($assembly_ID) = $file =~ m/^((bird|snake|fish)_\d+(C|E))_/ if ($file =~ m/^(bird|snake|fish)_\d+C|E_/);

# CSV file, with filename in first column

print $out "$assembly_ID";

for (my $i = 1; $i < 100; $i++){

# higher NG values might not be present if assembly is poor

if (defined $ng_values[$i]){

print $out ",$ng_values[$i]";

} else{

print $out ",0";

}

}

print $out "\n";

close($out);

}

}

# simple routine to add results to a pair of arrays that will be used for printing results later on

# if -csv option is used

sub store_results{

my ($desc, $result) = @_;

push(@headers,$desc);

push(@results,$result);

}

sub write_csv{

my ($file) = @_;

# create new output file name

my $output = $file;

$output =~ s/\.gz$//;

$output =~ s/\.(fa|fasta)$//;

$output .= ".csv";

# make some guesses of what might constitute the unique assembly ID

my $assembly_ID = $file;

($assembly_ID) = $file =~ m/^([A-Z]\d{1,2})_/ if ($file =~ m/^[A-Z]\d{1,2}_/);

($assembly_ID) = $file =~ m/^((bird|snake|fish)_\d+(C|E))_/ if ($file =~ m/^(bird|snake|fish)_\d+C|E_/);

open(my $out, ">", $output) or die "Can't create $output\n";

print $out "Assembly,";

foreach my $header (@headers){

print $out "$header,";

}

print $out "\n";

print $out "$assembly_ID,";

foreach my $result (@results){

print $out "$result,";

}

print $out "\n";

close($out);

}

####################################################assemblathon_stats_no_per_base_percent.pl####################################################

######FAlite.pm is needed for assemblathon_stats_no_per_base_percent.pl#####

######FAlite.pm#####

package FAlite;

use strict;

sub new {

my ($class, $fh) = @_;

if (ref $fh !~ /GLOB/)

{die ref $fh, "\n", "FAlite ERROR: expect a GLOB reference\n"}

my $this = bless {};

$this->{FH} = $fh;

while(<$fh>) {last if $_ =~ /\S/} # not supposed to have blanks, but...

my $firstline = $_;

if (not defined $firstline) {warn "FAlite: Empty\n"; return $this}

if ($firstline !~ /^>/) {warn "FAlite: Not FASTA formatted\n"; return $this}

$this->{LASTLINE} = $firstline;

chomp $this->{LASTLINE};

return $this;

}

sub nextEntry {

my ($this) = @_;

return 0 if not defined $this->{LASTLINE};

my $fh = $this->{FH};

my $def = $this->{LASTLINE};

my @seq;

my $lines_read = 0;

while(<$fh>) {

$lines_read++;

if ($_ =~ /^>/) {

$this->{LASTLINE} = $_;

chomp $this->{LASTLINE};

last;

}

push @seq, $_;

}

return 0 if $lines_read == 0;

chomp @seq;

my $entry = FAlite::Entry::new($def, \@seq);

return $entry;

}

package FAlite::Entry;

use overload '""' => 'all';

sub new {

my ($def, $seqarry) = @_;

my $this = bless {};

$this->{DEF} = $def;

$this->{SEQ} = join("", @$seqarry);

$this->{SEQ} =~ s/\s//g; # just in case more spaces

return $this;

}

sub def {shift->{DEF}}

sub seq {shift->{SEQ}}

sub all {my $e = shift; return $e->{DEF}."\n".$e->{SEQ}."\n"}

1;

__END__

=head1 NAME

FAlite;

=head1 SYNOPSIS

use FAlite;

my $fasta = new FAlite(\*STDIN);

while(my $entry = $fasta->nextEntry) {

$entry->def;

$entry->seq;

}

=head1 DESCRIPTION

FAlite is a package for parsing FASTA files and databases. The FASTA format is

widely used in bioinformatics. It consists of a definition line followed by

sequence with an arbitrary number of lines and line lengths.

A FASTA file looks like this:

>identifier descriptive text

GAATTC

A FASTA database looks like this:

>identifier1 some text describing this entry

GAATTC

ACTAGT

>identifier2 some text describing this entry

AAACCT

GCTAAT

=head2 Object

FAlite has two kinds of objects, the file and the entry.

my $fasta_file = new FAlite(\*STDIN); # or any other filehandle

$entry = $fasta_file->nextEntry; # single fasta fle

while(my $entry = $fasta_file->nextEntry) {

# canonical form of use for fasta database

}

The entry has two attributes (def and seq).

$entry->def; # access the def line

$entry->seq; # access the sequence

"$entry"; # overload to fasta file ($entry->def . "\n" . $entry->seq)

=head1 AUTHOR

Ian Korf (ikorf@sapiens.wustl.edu, http://sapiens.wustl.edu/~ikorf)

=head1 ACKNOWLEDGEMENTS

This software was developed at the Genome Sequencing Center at Washington

Univeristy, St. Louis, MO.

=head1 COPYRIGHT

Copyright (C) 1999 Ian Korf. All Rights Reserved.

=head1 DISCLAIMER

This software is provided "as is" without warranty of any kind.

=cut

######FAlite.pm#####

####Data for Table 1 and Table S2#####

### first Dovetail assembly

cd /genetics/elbers/

/opt/perl-5.10.1/bin/perl assemblathon_stats_no_per_base_percent.pl \

/genetics/pacbio/dromedary.fasta.original --csv

## summarizeAssembly.py is part of PBJelly scripts /opt/PBSuite_15.8.24/bin/summarizeAssembly.py

summarizeAssembly.py /genetics/pacbio/dromedary.fasta.original > /genetics/pacbio/dromedary.fasta.original.summary

python3 /opt/busco/scripts/run_BUSCO.py -c 70 -i/genetics/pacbio/dromedary.fasta.original \

-o dromedary -l /genetics/elbers/maker/mammalia_odb9/ --species human --mode genome > dromedary-busco.log 2>&1

### second PBJelly assembly

/opt/perl-5.10.1/bin/perl assemblathon_stats_no_per_base_percent.pl \

/genetics/pacbio/jelly.out.fasta --csv

summarizeAssembly.py /genetics/pacbio/jelly.out.fasta > /genetics/pacbio/jelly.out.fasta.summary

python3 /opt/busco/scripts/run_BUSCO.py -c 70 -i/genetics/pacbio/jelly.out.fasta \

-o dromedary-pbjelly -l /genetics/elbers/maker/mammalia_odb9/ --species human --mode genome > dromedary-pbjelly-busco.log 2>&1

### third Pilon assembly

/opt/perl-5.10.1/bin/perl assemblathon_stats_no_per_base_percent.pl \

/genetics/pacbio/jelly-split/dromedary.pbjelly.pilon.fasta --csv

summarizeAssembly.py /genetics/pacbio/jelly-split/dromedary.pbjelly.pilon.fasta > /genetics/pacbio/jelly-split/dromedary.pbjelly.pilon.fasta.summary

python3 /opt/busco/scripts/run_BUSCO.py -c 70 -i/genetics/pacbio/jelly.out.fasta \

-o dromedary-pbjelly-pilon -l /genetics/elbers/maker/mammalia_odb9/ --species human --mode genome > dromedary-pbjelly-pilon-busco.log 2>&1

### fourth Abyss assembly

/opt/perl-5.10.1/bin/perl assemblathon_stats_no_per_base_percent.pl \

/genetics/pacbio/dromedary.pbjelly.pilon.abyss.fasta --csv

summarizeAssembly.py /genetics/pacbio/dromedary.pbjelly.pilon.abyss.fasta > /genetics/pacbio/dromedary.pbjelly.pilon.abyss.fasta.summary

python3 /opt/busco/scripts/run_BUSCO.py -c 70 -i /genetics/pacbio/dromedary.pbjelly.pilon.abyss.fasta \

-o dromedary-pbjelly-pilon-abyss -l /genetics/elbers/maker/mammalia_odb9/ --species human --mode genome > dromedary-pbjelly-pilon-abyss-busco.log 2>&1

### fifth Pilon2 assembly (CamDro2)

/opt/perl-5.10.1/bin/perl assemblathon_stats_no_per_base_percent.pl \

/genetics/pacbio/dromedary.pbjelly.pilon.abyss.pilon.chromosomes.fasta --csv

summarizeAssembly.py /genetics/pacbio/dromedary.pbjelly.pilon.abyss.pilon.chromosomes.fasta > /genetics/pacbio/dromedary.pbjelly.pilon.abyss.pilon.chromosomes.fasta.summary

python3 /opt/busco/scripts/run_BUSCO.py -c 70 -i /genetics/pacbio/dromedary.pbjelly.pilon.abyss.pilon.chromosomes.fasta \

-o dromedary-pbjelly-pilon-abyss-pilon -l /genetics/elbers/maker/mammalia_odb9/ --species human --mode genome > dromedary-pbjelly-pilon-abyss-pilon-bases-busco.log 2>&1

### sixth CamDro1

/opt/perl-5.10.1/bin/perl assemblathon_stats_no_per_base_percent.pl \

/genetics/pacbio/GCA_000803125.1_Cdrom64K_genomic.fna --csv

summarizeAssembly.py /genetics/pacbio/GCA_000803125.1_Cdrom64K_genomic.fna > /genetics/pacbio/GCA_000803125.1_Cdrom64K_genomic.fna.summary

python3 /opt/busco/scripts/run_BUSCO.py -c 70 -i /genetics/pacbio/GCA_000803125.1_Cdrom64K_genomic.fna \

-o fitak-dromedary -l /genetics/elbers/maker/mammalia_odb9/ --species human --mode genome > fitak-dromedary-busco.log 2>&1

### seventh Asian dromedary

cd /genetics/pacbio

wget ftp://ftp.ncbi.nlm.nih.gov/genomes/all/GCA/000/767/585/GCA_000767585.1_PRJNA234474_Ca_dromedarius_V1.0/GCA_000767585.1_PRJNA234474_Ca_dromedarius_V1.0_genomic.fna.gz

gunzip GCA_000767585.1_PRJNA234474_Ca_dromedarius_V1.0_genomic.fna.gz

cd /genetics/elbers

/opt/perl-5.10.1/bin/perl assemblathon_stats_no_per_base_percent.pl \

/genetics/pacbio/GCA_000767585.1_PRJNA234474_Ca_dromedarius_V1.0_genomic.fna --csv

summarizeAssembly.py /genetics/pacbio/GCA_000767585.1_PRJNA234474_Ca_dromedarius_V1.0_genomic.fna > /genetics/pacbio/GCA_000767585.1_PRJNA234474_Ca_dromedarius_V1.0_genomic.fna.summary

python3 /opt/busco/scripts/run_BUSCO.py -c 70 -i /genetics/pacbio/GCA_000767585.1_PRJNA234474_Ca_dromedarius_V1.0_genomic.fna \

-o asian-dromedary -l /genetics/elbers/maker/mammalia_odb9/ --species human --mode genome > 01asian-dromedary-busco.log 2>&1

#########################

# STEP 22 Prepare input files for MAKER annotation

#########################

##### for dromedary.pbjelly.pilon.abyss.pilon.chromosomes.fasta assembly #####

cd /genetics/pacbio/

mkdir maker-run1

mkdir maker-run2

cd /genetics/pacbio/maker-run1/

## first run BUSCO to train Augustus

python3 /opt/busco/scripts/run_BUSCO.py -i /genetics/pacbio/dromedary.pbjelly.pilon.abyss.pilon.chromosomes.fasta -c 60 \

-o dromedary-pbjelly-pilon-abyss-pilon -l /genetics/elbers/eukaryota_odb9/ --species human --mode genome --long > dromedary-pbjelly-pilon-abyss-pilon-busco.log 2>&1 &

# copy augustus training to augustus species folder

cd /genetics/pacbio/maker-run1/run_dromedary-pbjelly-pilon-abyss-pilon/augustus_output/retraining_parameters

mkdir /genetics/elbers/augustus/config/species/BUSCO_dromedary-pbjelly-pilon-abyss-pilon

mv BUSCO_dromedary-pbjelly-pilon-abyss-pilon_*_parameters.cfg BUSCO_dromedary-pbjelly-pilon-abyss-pilon_parameters.cfg

cp * /genetics/elbers/augustus/config/species/BUSCO_dromedary-pbjelly-pilon-abyss-pilon/.

cd /genetics/elbers/augustus/config/species/BUSCO_dromedary-pbjelly-pilon-abyss-pilon/

## second run repeat modeler to create custom repeat library

cd /genetics/pacbio/maker-run1/

/opt/RepeatModeler-open-1.0.10/BuildDatabase -name dromedary-pbjelly-pilon-abyss-pilon -engine ncbi /genetics/pacbio/dromedary.pbjelly.pilon.abyss.pilon.chromosomes.fasta

/opt/RepeatModeler-open-1.0.10/RepeatModeler -pa 60 -engine ncbi -database dromedary-pbjelly-pilon-abyss-pilon > dromedary-pbjelly-pilon-abyss-pilon-repeat.log 2>&1 &

# dromedary-pbjelly-pilon-abyss-pilon-families.fa is custom repeatlib

# Run blastx then ProtExcluder to excluce known protein sequences from RepeatModeler library

/usr/bin/blastx -num_threads 70 -db /genetics/elbers/maker/uniprot_sprot.fasta -evalue 1e-6 \

-query dromedary-pbjelly-pilon-abyss-pilon-families.fa -out dromedary-pbjelly-pilon-abyss-pilon-families.fa.blast &

/opt/ProtExcluder1.1/ProtExcluder.pl -f 50 dromedary-pbjelly-pilon-abyss-pilon-families.fa.blast dromedary-pbjelly-pilon-abyss-pilon-families.fa

mv temp dromedary-pbjelly-pilon-abyss-pilon-families.fa2

## fourth create genemark gene predictions

cd /genetics/pacbio/maker-run1/

mkdir genemark

cd /genetics/pacbio/maker-run1/genemark/

/opt/gm_et_linux_64/gmes_petap/gmes_petap.pl --ES --cores 60 -v \

--sequence /genetics/pacbio/dromedary.pbjelly.pilon.abyss.pilon.chromosomes.fasta \

> dromedary-pbjelly-pilon-abyss-pilon-genemark.log 2>&1 &

cp /genetics/pacbio/maker-run1/genemark/output/gmhmm.mod /genetics/pacbio/maker-run1/dromedary-pbjelly-pilon-abyss-pilon-gmhmm.mod

## fifth need to map bactrian rnaseq to bactrian assembly to get altests

####bactrian-camel-rnaseq-accessions.txt

SRR527309

SRR527310

SRR527311

SRR527312

SRR527313

SRR527314

SRR527315

SRR527316

SRR527317

####bactrian-camel-rnaseq-accessions.txt

### 1 get raw reads in SRA format

cd /genetics/elbers/ragout/

while read i;do

/opt/sratoolkit.2.8.2-1/bin/prefetch $i

done < bactrian-camel-rnaseq-accessions.txt &

mv /home/elbersj/ncbi/public/sra/* /genetics/elbers/ragout/.

### 2 get bactrian camel genome

wget ftp://ftp.ncbi.nlm.nih.gov/genomes/all/GCF/000/767/855/GCF_000767855.1_Ca_bactrianus_MBC_1.0/GCF_000767855.1_Ca_bactrianus_MBC_1.0_genomic.fna.gz

### 3 rename it to be compatible with ragout (not for HISAT2 mapping)

mv GCF_000767855.1_Ca_bactrianus_MBC_1.0_genomic.fna GCF_0007678551_Ca_bactrianus_MBC_10_genomic.fna

### 4 convert SRA files into GZIPPED FASTQ files

parallel '/opt/sratoolkit.2.8.2-1/bin/fastq-dump --gzip --defline-seq '@$sn[_$rn]/$ri' --split-files {}.sra' < bactrian-camel-rnaseq-accessions.txt &

### 5 perform quality and adpater filtering

while read i;do

/opt/bbmap/bbduk.sh threads=70 in1=${i}_1.fastq.gz in2=${i}_2.fastq.gz out1=${i}-trim_1.fq.gz \

out2=${i}-trim_2.fq.gz \

ref=/opt/bbmap/resources/adapters.fa ktrim=r k=23 mink=11 hdist=1 tpe tbo qtrim=rl trimq=15

done < bactrian-camel-rnaseq-accessions.txt

### 6 combine the reads

cat *-trim_1.fq.gz > bactrian-camel-rnaseq-reads-trim_1.fq.gz

cat *-trim_2.fq.gz > bactrian-camel-rnaseq-reads-trim_2.fq.gz

### 7 build an hisat2 index

/opt/hisat2-2.1.0/hisat2-build GCF_0007678551_Ca_bactrianus_MBC_10_genomic.fna GCF_0007678551_Ca_bactrianus_MBC_10_genomic &

### 8 map reads to reference genome

/opt/hisat2-2.1.0/hisat2 --max-intronlen 100000 -p 70 -x GCF_0007678551_Ca_bactrianus_MBC_10_genomic -1 bactrian-camel-rnaseq-reads-trim_1.fq.gz \

-2 bactrian-camel-rnaseq-reads-trim_2.fq.gz | samtools view -bS - | samtools sort -o bactrian-rnaseq-reads-GCF_0007678551_Ca_bactrianus_MBC_10_genomic.fna.bam - &

### 9 make gtf file with stringtie

/opt/stringtie-1.3.3b/stringtie bactrian-rnaseq-reads-GCF_0007678551_Ca_bactrianus_MBC_10_genomic.fna.bam -v -p 70 -o bactrian-rnaseq-reads-GCF_0007678551_Ca_bactrianus_MBC_10_genomic.fna.gtf > stringtie.log 2>&1 &

### 10 create FASTA sequence of cDNA sequences

/opt/gffread-0.9.9/gffread -w bactrian-rnaseq-reads-GCF_0007678551_Ca_bactrianus_MBC_10_genomic.fna.fa -g GCF_0007678551_Ca_bactrianus_MBC_10_genomic.fna \

bactrian-rnaseq-reads-GCF_0007678551_Ca_bactrianus_MBC_10_genomic.fna.gtf

### 11 get rid of spaces " " and equal signs "=", converting them into underscores "_"

perl -pi -e "s/ |=/_/g" bactrian-rnaseq-reads-GCF_0007678551_Ca_bactrianus_MBC_10_genomic.fna.fa

## sixth need to map dromedary rnaseq reads from SRA and Mark Rogers to dromedary-pbjelly-racon-pilon assembly to get ests

####accession-list-camel-rnaseq.txt####

SRR527275

SRR527276

SRR527277

SRR527278

SRR527279

SRR527280

SRR527281

SRR527285

SRR527288

SRR527292

####accession-list-camel-rnaseq.txt####

#####THESE STEPS WERE DONE ON SUPERMIKE-II#####

### 1 get SRA reads from accession-list-camel-rnaseq.txt

while read i;do

~/bin/sratoolkit.2.8.1-2-centos_linux64/bin/prefetch $i

mv /work/jelber2/ncbi/sra/$i.sra $WORK_DIR

done < /work/jelber2/dovetail/accession-list-camel-rnaseq.txt

### 2 convert .sra files into fastq files

~/bin/sratoolkit.2.8.1-2-centos_linux64/bin/fastq-dump --gzip --defline-seq '@$sn[_$rn]/$ri' --split-files file.sra

### 3 put all R1 reads into file

cat *_1.fastq.gz > camel-rnaseq-read1.fq.gz

### 4 put all R2 reads into file

cat *_2.fastq.gz > camel-rnaseq-read2.fq.gz

### 5 Quality and adapter trim FASTQ reads

~/bin/bbmap-37.25/bbduk.sh in1=camel-rnaseq-read1.fq.gz in2=camel-rnaseq-read2.fq.gz out1=camel-rnaseq-read1-trim.fq.gz \

out2=camel-rnaseq-read2-trim.fq.gz \

ref=~/bin/bbmap-37.25/resources/adapters.fa ktrim=r k=23 mink=11 hdist=1 tpe tbo qtrim=rl trimq=15

### 6 Analyze Rogers camel RNAseq data

#### 6a Combine read 1 for FASTQ reads

cat 10_CAGATC_L002_R1_001.fastq.gz \

11_ATGTCA_L002_R1_001.fastq.gz \

13_CCGTCC_L002_R1_001.fastq.gz \

14_GTCCGC_L002_R1_001.fastq.gz \

15_GTGAAA_L002_R1_001.fastq.gz \

1_CGATGT_L001_R1_001.fastq.gz \

2_TGACCA_L001_R1_001.fastq.gz \

3_GCCAAT_L001_R1_001.fastq.gz \

5_CTTGTA_L001_R1_001.fastq.gz \

6_AGTCAA_L001_R1_001.fastq.gz \

7_AGTTCC_L001_R1_001.fastq.gz \

9_ACAGTG_L002_R1_001.fastq.gz > rogers-rnaseq-read1.fq.gz

#### 6b Combine read 2 for FASTQ reads

cat 10_CAGATC_L002_R2_001.fastq.gz \

11_ATGTCA_L002_R2_001.fastq.gz \

13_CCGTCC_L002_R2_001.fastq.gz \

14_GTCCGC_L002_R2_001.fastq.gz \

15_GTGAAA_L002_R2_001.fastq.gz \

1_CGATGT_L001_R2_001.fastq.gz \

2_TGACCA_L001_R2_001.fastq.gz \

3_GCCAAT_L001_R2_001.fastq.gz \

5_CTTGTA_L001_R2_001.fastq.gz \

6_AGTCAA_L001_R2_001.fastq.gz \

7_AGTTCC_L001_R2_001.fastq.gz \

9_ACAGTG_L002_R2_001.fastq.gz > rogers-rnaseq-read2.fq.gz

#### 6c Quality and adapter trimming

~/bin/bbmap-37.25/bbduk.sh in1=rogers-rnaseq-read1.fq.gz in2=rogers-rnaseq-read2.fq.gz out1=rogers-rnaseq-read1-trim.fq.gz \

out2=rogers-rnaseq-read2-trim.fq.gz \

ref=~/bin/bbmap-37.25/resources/adapters.fa ktrim=r k=23 mink=11 hdist=1 tpe tbo qtrim=rl trimq=15

#####THESE STEPS DONE ON SUPERMIKE-II#####

#### 7 these steps done on Genetics Server

##### copy rogers-rnaseq-read1-trim.fq.gz rogers-rnaseq-read2-trim.fq.gz camel-rnaseq-read1-trim.fq camel-rnaseq-read2-trim.fq to Genetics Server

##### concatenate read1 and read2

cat rogers-rnaseq-read1-trim.fq.gz camel-rnaseq-read1-trim.fq.gz > dromedary-rnaseq-read1-trim.fq.gz

cat rogers-rnaseq-read2-trim.fq.gz camel-rnaseq-read2-trim.fq.gz > dromedary-rnaseq-read2-trim.fq.gz

rm rogers-rnaseq-read1-trim.fq.gz camel-rnaseq-read1-trim.fq.gz rogers-rnaseq-read2-trim.fq.gz camel-rnaseq-read2-trim.fq.gz

#### 8 build hisat2 index using the abyss assembly

/opt/hisat2-2.1.0/hisat2-build dromedary.pbjelly.pilon.abyss.pilon.chromosomes.fasta dromedary.pbjelly.pilon.abyss.pilon.chromosomes

#### 9 map transcriptome reads to the abyss assembly

/opt/hisat2-2.1.0/hisat2 --max-intronlen 100000 -p 35 -x dromedary.pbjelly.pilon.abyss.pilon.chromosomes. -1 dromedary-rnaseq-read1-trim.fq.gz \

-2 dromedary-rnaseq-read2-trim.fq.gz 2> dromedary.rnaseq.mapped.to.dromedary.pbjelly.pilon.abyss.pilon.chromosomes.log | samtools view -@35 -bS - | samtools sort -@35 -o dromedary.rnaseq.mapped.to.dromedary.pbjelly.pilon.abyss.pilon.chromosomes.bam - &

#### 10 create gtf file

/opt/stringtie-1.3.3b/stringtie dromedary.rnaseq.mapped.to.dromedary.pbjelly.pilon.abyss.pilon.chromosomes.bam -v -p 35 -o dromedary.pbjelly.pilon.abyss.pilon.chromosomes.gtf > stringtie.log 2>&1 &

#### 11 create FASTA file to get cDNA sequences

/opt/gffread-0.9.9/gffread -w dromedary.rnaseq.mapped.to.dromedary.pbjelly.pilon.abyss.pilon.chromosomes.fasta -g dromedary.pbjelly.pilon.abyss.pilon.chromosomes.fasta \

dromedary.pbjelly.pilon.abyss.pilon.chromosomes.gtf

#### 12 change " " and "=" to "_" to make MAKER happy

perl -pi -e "s/ |=/_/g" dromedary.rnaseq.mapped.to.dromedary.pbjelly.pilon.abyss.pilon.chromosomes.fasta

## seventh get proteins

### Get previously predicted proteins from dromedary with AED <0.75

cd /genetics/elbers/maker/

wget http://datadryad.org/bitstream/handle/10255/dryad.89613/Drom800.tar.gz?sequence=2

mv Drom800.tar.gz?sequence=2 Drom800.tar.gz

tar xzf Drom800.tar.gz

cd Drom800_data

#### get proteins with AED <0.75

/opt/seqtk/seqtk seq -l0 Drom800.all-proteins.fasta | pcregrep -M "AED:0.[0-6][0-9].+\n\w+|AED:0.[7][0-4].+\n\w+" > ../Drom800.proteins_0-0.74_AED.fasta

### combine with proteins from other organisms

wget ftp://ftp.ncbi.nlm.nih.gov/genomes/all/GCF/000/003/055/GCF_000003055.6_Bos_taurus_UMD_3.1.1/GCF_000003055.6_Bos_taurus_UMD_3.1.1_protein.faa.gz

gunzip GCF_000003055.6_Bos_taurus_UMD_3.1.1_protein.faa.gz

wget ftp://ftp.ncbi.nlm.nih.gov/genomes/all/GCF/000/311/805/GCF_000311805.1_CB1/GCF_000311805.1_CB1_protein.faa.gz

gunzip GCF_000311805.1_CB1_protein.faa.gz

wget ftp://ftp.ncbi.nlm.nih.gov/genomes/all/GCF/000/164/845/GCF_000164845.2_Vicugna_pacos-2.0.2/GCF_000164845.2_Vicugna_pacos-2.0.2_protein.faa.gz

gunzip GCF_000164845.2_Vicugna_pacos-2.0.2_protein.faa.gz

cat GCF_000003055.6_Bos_taurus_UMD_3.1.1_protein.faa GCF_000311805.1_CB1_protein.faa GCF_000164845.2_Vicugna_pacos-2.0.2_protein.faa Drom800.proteins_0-0.74_AED.fasta > ncbi-camelid-cow-old-maker-proteins.fa

#########################

# Step 23 annotate dromedary-pbjelly-pilon-abyss assembly using first run of MAKER

#########################

# predict genes on contigs greater than 10,000 bases and longer!

## first run MAKER

cd /genetics/pacbio/maker-run1/

#####maker_bopts.ctl#####

#-----BLAST and Exonerate Statistics Thresholds

blast_type=ncbi+ #set to 'ncbi+', 'ncbi' or 'wublast'

pcov_blastn=0.8 #Blastn Percent Coverage Threhold EST-Genome Alignments

pid_blastn=0.85 #Blastn Percent Identity Threshold EST-Genome Aligments

eval_blastn=1e-10 #Blastn eval cutoff

bit_blastn=40 #Blastn bit cutoff

depth_blastn=0 #Blastn depth cutoff (0 to disable cutoff)

pcov_blastx=0.5 #Blastx Percent Coverage Threhold Protein-Genome Alignments

pid_blastx=0.4 #Blastx Percent Identity Threshold Protein-Genome Aligments

eval_blastx=1e-06 #Blastx eval cutoff

bit_blastx=30 #Blastx bit cutoff

depth_blastx=0 #Blastx depth cutoff (0 to disable cutoff)

pcov_tblastx=0.8 #tBlastx Percent Coverage Threhold alt-EST-Genome Alignments

pid_tblastx=0.85 #tBlastx Percent Identity Threshold alt-EST-Genome Aligments

eval_tblastx=1e-10 #tBlastx eval cutoff

bit_tblastx=40 #tBlastx bit cutoff

depth_tblastx=0 #tBlastx depth cutoff (0 to disable cutoff)

pcov_rm_blastx=0.5 #Blastx Percent Coverage Threhold For Transposable Element Masking

pid_rm_blastx=0.4 #Blastx Percent Identity Threshold For Transposbale Element Masking

eval_rm_blastx=1e-06 #Blastx eval cutoff for transposable element masking

bit_rm_blastx=30 #Blastx bit cutoff for transposable element masking

ep_score_limit=20 #Exonerate protein percent of maximal score threshold

en_score_limit=20 #Exonerate nucleotide percent of maximal score threshold

#####maker_bopts.ctl#####

#####maker_exe.ctl#####

#-----Location of Executables Used by MAKER/EVALUATOR

makeblastdb=/usr/bin/makeblastdb #location of NCBI+ makeblastdb executable

blastn=/usr/bin/blastn #location of NCBI+ blastn executable

blastx=/usr/bin/blastx #location of NCBI+ blastx executable

tblastx=/usr/bin/tblastx #location of NCBI+ tblastx executable

formatdb= #location of NCBI formatdb executable

blastall= #location of NCBI blastall executable

xdformat= #location of WUBLAST xdformat executable

blasta= #location of WUBLAST blasta executable

RepeatMasker=/opt/RepeatMasker/RepeatMasker #location of RepeatMasker executable

exonerate=/usr/bin/exonerate #location of exonerate executable

#-----Ab-initio Gene Prediction Algorithms

snap= #location of snap executable

gmhmme3=/opt/gm_et_linux_64/gmes_petap/gmhmme3 #location of eukaryotic genemark executable

gmhmmp= #location of prokaryotic genemark executable

augustus=/opt/maker/exe/augustus/bin/augustus #location of augustus executable

fgenesh= #location of fgenesh executable

tRNAscan-SE= #location of trnascan executable

snoscan= #location of snoscan executable

#-----Other Algorithms

probuild=/opt/gm_et_linux_64/gmes_petap/probuild

#####maker_exe.ctl#####

#####maker_opts.ctl#####

#-----Genome (these are always required)

genome=/genetics/pacbio/dromedary.pbjelly.pilon.abyss.pilon.chromosomes.fasta #genome sequence (fasta file or fasta embeded in GFF3 file)

organism_type=eukaryotic #eukaryotic or prokaryotic. Default is eukaryotic

#-----Re-annotation Using MAKER Derived GFF3

maker_gff= #MAKER derived GFF3 file

est_pass=0 #use ESTs in maker_gff: 1 = yes, 0 = no

altest_pass=0 #use alternate organism ESTs in maker_gff: 1 = yes, 0 = no

protein_pass=0 #use protein alignments in maker_gff: 1 = yes, 0 = no

rm_pass=0 #use repeats in maker_gff: 1 = yes, 0 = no

model_pass=0 #use gene models in maker_gff: 1 = yes, 0 = no

pred_pass=0 #use ab-initio predictions in maker_gff: 1 = yes, 0 = no

other_pass=0 #passthrough anyything else in maker_gff: 1 = yes, 0 = no

#-----EST Evidence (for best results provide a file for at least one)

est=/genetics/pacbio/dromedary.rnaseq.mapped.to.dromedary.pbjelly.pilon.abyss.pilon.chromosomes.fasta #set of ESTs or assembled mRNA-seq in fasta format

altest=/genetics/elbers/ragout/bactrian-rnaseq-reads-GCF_0007678551_Ca_bactrianus_MBC_10_genomic.fna.fa #EST/cDNA sequence file in fasta format from an alternate organism

est_gff= #aligned ESTs or mRNA-seq from an external GFF3 file

altest_gff= #aligned ESTs from a closly relate species in GFF3 format

#-----Protein Homology Evidence (for best results provide a file for at least one)

protein=/genetics/elbers/maker/ncbi-camelid-cow-old-maker-proteins.fa #protein sequence file in fasta format (i.e. from mutiple oransisms)

protein_gff= #aligned protein homology evidence from an external GFF3 file

#-----Repeat Masking (leave values blank to skip repeat masking)

model_org=all #select a model organism for RepBase masking in RepeatMasker

rmlib=dromedary-pbjelly-pilon-abyss-pilon-families.fa2 #provide an organism specific repeat library in fasta format for RepeatMasker

repeat_protein=/opt/maker/data/te_proteins.fasta #provide a fasta file of transposable element proteins for RepeatRunner

rm_gff= #pre-identified repeat elements from an external GFF3 file

prok_rm=0 #forces MAKER to repeatmask prokaryotes (no reason to change this), 1 = yes, 0 = no

softmask=1 #use soft-masking rather than hard-masking in BLAST (i.e. seg and dust filtering)

#-----Gene Prediction

snaphmm= #SNAP HMM file

gmhmm=dromedary-pbjelly-pilon-abyss-pilon-gmhmm.mod #GeneMark HMM file

augustus_species=BUSCO_dromedary-pbjelly-pilon-abyss-pilon #Augustus gene prediction species model

fgenesh_par_file= #FGENESH parameter file

pred_gff= #ab-initio predictions from an external GFF3 file

model_gff= #annotated gene models from an external GFF3 file (annotation pass-through)

est2genome=0 #infer gene predictions directly from ESTs, 1 = yes, 0 = no

protein2genome=0 #infer predictions from protein homology, 1 = yes, 0 = no

trna=0 #find tRNAs with tRNAscan, 1 = yes, 0 = no

snoscan_rrna= #rRNA file to have Snoscan find snoRNAs

unmask=0 #also run ab-initio prediction programs on unmasked sequence, 1 = yes, 0 = no

#-----Other Annotation Feature Types (features MAKER doesn't recognize)

other_gff= #extra features to pass-through to final MAKER generated GFF3 file

#-----External Application Behavior Options

alt_peptide=C #amino acid used to replace non-standard amino acids in BLAST databases

cpus=1 #max number of cpus to use in BLAST and RepeatMasker (not for MPI, leave 1 when using MPI)

#-----MAKER Behavior Options

max_dna_len=100000 #length for dividing up contigs into chunks (increases/decreases memory usage)

min_contig=10000 #skip genome contigs below this length (under 10kb are often useless)

pred_flank=200 #flank for extending evidence clusters sent to gene predictors

pred_stats=1 #report AED and QI statistics for all predictions as well as models

AED_threshold=1 #Maximum Annotation Edit Distance allowed (bound by 0 and 1)

min_protein=30 #require at least this many amino acids in predicted proteins

alt_splice=1 #Take extra steps to try and find alternative splicing, 1 = yes, 0 = no

always_complete=0 #extra steps to force start and stop codons, 1 = yes, 0 = no

map_forward=0 #map names and attributes forward from old GFF3 genes, 1 = yes, 0 = no

keep_preds=1 #Concordance threshold to add unsupported gene prediction (bound by 0 and 1)

split_hit=10000 #length for the splitting of hits (expected max intron size for evidence alignments)

single_exon=1 #consider single exon EST evidence when generating annotations, 1 = yes, 0 = no

single_length=250 #min length required for single exon ESTs if 'single_exon is enabled'

correct_est_fusion=0 #limits use of ESTs in annotation to avoid fusion genes

tries=2 #number of times to try a contig if there is a failure for some reason

clean_try=0 #remove all data from previous run before retrying, 1 = yes, 0 = no

clean_up=0 #removes theVoid directory with individual analysis files, 1 = yes, 0 = no

TMP= #specify a directory other than the system default temporary directory for temporary files

#####maker_opts.ctl#####

mpiexec -n 70 /opt/maker/bin/maker -fix_nucleotides >> dromedary.pbjelly.pilon.abyss.pilon.chromosomes.fasta.output.txt 2>&1 &

## second get GFF3 file

/opt/maker/bin/gff3_merge -d dromedary.pbjelly.pilon.abyss.pilon.chromosomes.maker.output/dromedary.pbjelly.pilon.abyss.pilon.chromosomes_master_datastore_index.log -o dromedary.pbjelly.pilon.abyss.pilon.all.gff -n &

/opt/maker/bin/gff3_merge -d dromedary.pbjelly.pilon.abyss.pilon.chromosomes.maker.output/dromedary.pbjelly.pilon.abyss.pilon.chromosomes_master_datastore_index.log -o dromedary.pbjelly.pilon.abyss.pilon.all.with.sequences.gff &

## third get the FASTA sequences

### i get all fasta sequences

/opt/maker/bin/fasta_merge -d dromedary.pbjelly.pilon.abyss.pilon.chromosomes.maker.output/dromedary.pbjelly.pilon.abyss.pilon.chromosomes_master_datastore_index.log -o dromedary.pbjelly.pilon.abyss.pilon.all.fasta &

### ii Download Uniprot_Trembl release 2018_04

cd /genetics/elbers/maker

wget 'ftp://ftp.uniprot.org/pub/databases/uniprot/current_release/knowledgebase/complete/uniprot_sprot.fasta.gz' -O uniprot_trembl_release_2018_04.fasta.gz

pigz -kd uniprot_trembl_release_2018_04.fasta.gz

# makedb

/opt/diamond/diamond_0.9.19 makedb --in uniprot_trembl_release_2018_04.fasta.gz --db uniprot_trembl_release_2018_04.fasta --threads 75

### iii run ideel (https://github.com/mw55309/ideel)

cd /genetics/pacbio/maker-run1/ideel/

wget https://raw.githubusercontent.com/jelber2/ideel/master/scripts/hist.R

######hist.R#####

#!/usr/bin/env Rscript

# get command line arguments as an array

args <- commandArgs(trailingOnly = TRUE)

# files

filein <- args[1]

fileout <- args[2]

# data

data <- read.table(filein, sep="\t", header=FALSE)

# breaks

bks <- seq(0,max(data$V1/data$V2)+1,by=0.05)

# main hist

png(fileout, width=800, height=800, type="cairo")

hist(data$V1/data$V2, breaks=bks, col="red", xlim=c(0,2), xlab="Query sequence length / Subject sequence length", ylab="Frequency", main=filein)

dev.off()

# scaled hist

fileout <- gsub(".png",".500.png", fileout)

png(fileout, width=800, height=800, type="cairo")

hist(data$V1/data$V2, ylim=c(0,500), breaks=bks, col="purple", xlim=c(0,2), xlab="Query sequence length / Subject sequence length", ylab="Frequency", main=filein)

dev.off()

#####hist.R#####

chmod u+x hist.R

### iv ideel fitak proteins

#### make proteins file

cd /genetics/elbers/maker

/opt/seqtk/seqtk seq -l0 ncbi-camelid-cow-old-maker-proteins.fa |paste - -|grep "AED:" |tr "\t" "\n" > fitak-old-maker.proteins.fasta

#### mapping

mkdir -p /genetics/pacbio/fitak/ideel

/opt/diamond/diamond_0.9.19 blastp --threads 75 --max-target-seqs 1 --db /genetics/elbers/maker/uniprot_trembl_release_2018_04.fasta --query fitak-old-maker.proteins.fasta \

--outfmt 6 qlen slen --out /genetics/pacbio/fitak/ideel/fitak-old-maker.proteins.fasta.against.uniprot_trembl_release_2018_04.blast > /genetics/pacbio/fitak/ideel/fitak-old-maker.proteins.fasta.against.uniprot_trembl_release_2018_04.blast.log 2>&1 &

## Determine the "best" cut-off value for AED (annotation edit distance)

cd /genetics/pacbio/maker-run1/

wget https://raw.githubusercontent.com/mscampbell/Genome_annotation/master/AED_cdf_generator.pl

perl AED_cdf_generator.pl -b 0.025 dromedary.pbjelly.pilon.abyss.pilon.all.gff > maker-run1.aed.cum.frac.below.txt

# AED 0.50 is "best" cut-off value as 0.784 of transcripts have AED <= 0.50

## fourth get transcript and protein sequences

### i get high quality proteins and transcripts

/opt/seqtk/seqtk seq -l0 dromedary.pbjelly.pilon.abyss.pilon.all.fasta.all.maker.proteins.fasta | pcregrep -M " AED:0.[0-4][0-9].+\n\w+| AED:0.50.+\n\w+" \

> dromedary.pbjelly.pilon.abyss.pilon.all.fasta.all.maker.proteins.0-0.50_AED.fasta

/opt/seqtk/seqtk seq -l0 dromedary.pbjelly.pilon.abyss.pilon.all.fasta.all.maker.transcripts.fasta | pcregrep --buffer-size 3000000000 -M " AED:0.[0-4][0-9].+\n\w+| AED:0.50.+\n\w+" \

> dromedary.pbjelly.pilon.abyss.pilon.all.fasta.all.maker.transcripts.0-0.50_AED.fasta

## ii run ideel

mkdir ideel

/opt/diamond/diamond_0.9.19 blastp --threads 75 --max-target-seqs 1 --db /genetics/elbers/maker/uniprot_trembl_release_2018_04.fasta \

--query dromedary.pbjelly.pilon.abyss.pilon.all.fasta.all.maker.proteins.0-0.50_AED.fasta \

--outfmt 6 qlen slen --out ideel/dromedary.pbjelly.pilon.abyss.pilon.all.fasta.all.maker.proteins.0-0.50_AED.fasta.against.uniprot_trembl_release_2018_04.fasta.blast > diamond.log 2>&1

echo -e 'test <- read.table("ideel/dromedary.pbjelly.pilon.abyss.pilon.all.fasta.all.maker.proteins.0-0.50_AED.fasta.against.uniprot_trembl_release_2018_04.fasta.blast")\ntest2 <- test$V1/test$V2\ntest3 <- test2[test2 >= 0.85]\ntest4 <- test3[test3 <= 1.15]\ntest5 <- test2[test2 < 0.85]\ncat("\n")\npaste("There are",length(test2),"protein hits, and", length(test4), round(length(test4)/length(test2)*100,2), "%","are between 0.85 and 1.15 (querylength/subjectlength).")\ncat("\n")\npaste("There are", length(test5), round(length(test5)/length(test2)*100,2),"%", "protein hits less than 0.85 (querylength/subjectlength).")\ncat("\n")'> stats.R

Rscript stats.R

# "There are 32296 protein hits, and 17267 53.46 % are between 0.85 and 1.15 (querylength/subjectlength)."

# "There are 12716 39.37 % protein hits less than 0.85 (querylength/subjectlength)."

# Fitak old proteins

/genetics/pacbio/maker-run1/ideel/hist.R fitak-old-maker.proteins.fasta.against.uniprot_trembl_release_2018_04.blast fitak-old-maker.proteins.fasta.against.uniprot_trembl_release_2018_04.blast.png

echo -e 'test <- read.table("fitak-old-maker.proteins.fasta.against.uniprot_trembl_release_2018_04.blast")\ntest2 <- test$V1/test$V2\ntest3 <- test2[test2 >= 0.85]\ntest4 <- test3[test3 <= 1.15]\ntest5 <- test2[test2 < 0.85]\ncat("\n")\npaste("There are",length(test2),"protein hits, and", length(test4), round(length(test4)/length(test2)*100,2), "%","are between 0.85 and 1.15 (querylength/subjectlength).")\ncat("\n")\npaste("There are", length(test5), round(length(test5)/length(test2)*100,2),"%", "protein hits less than 0.85 (querylength/subjectlength).")\ncat("\n")'> stats.R

Rscript stats.R

# "There are 21259 protein hits, and 11631 54.71 % are between 0.85 and 1.15 (querylength/subjectlength)."

# "There are 7703 36.23 % protein hits less than 0.85 (querylength/subjectlength)."

## fourth train augustus with maker run1 output

### make augustus input

cd /genetics/pacbio/maker-run1

awk -F"\t" -v OFS="\t" '$1=="##gff-version 3"||$2=="maker"' dromedary.pbjelly.pilon.abyss.pilon.all.gff | \

awk -F"\t" -v OFS="\t" '$1=="##gff-version 3"||$3=="CDS"' > dromedary.pbjelly.pilon.abyss.pilon.all.gff.maker1.input.for.augustus.gff

## fifth run augustus

/opt/maker/exe/augustus/scripts/autoAug.pl \

--genome=/genetics/pacbio/dromedary.pbjelly.pilon.abyss.pilon.chromosomes.fasta \

--species=dromedary-pbjelly-pilon-abyss-pilon-run2 \

--cdna=dromedary.pbjelly.pilon.abyss.pilon.all.maker.transcripts.cdna.for.augustus.training.fasta \

--trainingset=dromedary.pbjelly.pilon.abyss.pilon.all.gff.maker1.input.for.augustus.gff \

-v -v –v --singleCPU \

--noninteractive > etraining.log 2>&1 &

## fifth make a id map for all genes in gff file (i.e., instead of maker-gene-01124356 convert to Cadr-000000001)

/opt/maker/bin/maker_map_ids --prefix Cadr_ --justify 8 dromedary.pbjelly.pilon.abyss.pilon.all.gff > dromedary.pbjelly.pilon.abyss.pilon.all.gff.id.map

## sixth blast high quality maker proteins against uniprot trembl

/opt/diamond/diamond_0.9.19 blastp --threads 75 --max-target-seqs 1 --db /genetics/elbers/maker/uniprot_trembl_release_2018_04.fasta \

--evalue 1e-6 --query dromedary.pbjelly.pilon.abyss.pilon.all.fasta.all.maker.proteins.0-0.50_AED.fasta --outfmt 6 \

--out dromedary.pbjelly.pilon.abyss.pilon.all.fasta.all.maker.proteins.fasta.blast > diamond.maker.log 2>&1

## seventh make copies of files (because scripts below overwrite input files)

cp dromedary.pbjelly.pilon.abyss.pilon.all.gff dromedary.pbjelly.pilon.abyss.pilon.all.renamed.gff

cp dromedary.pbjelly.pilon.abyss.pilon.all.fasta.all.maker.proteins.0-0.50_AED.fasta dromedary.pbjelly.pilon.abyss.pilon.all.fasta.all.maker.proteins.renamed.fasta

cp dromedary.pbjelly.pilon.abyss.pilon.all.fasta.all.maker.transcripts.0-0.50_AED.fasta dromedary.pbjelly.pilon.abyss.pilon.all.fasta.all.maker.transcripts.renamed.fasta

cp dromedary.pbjelly.pilon.abyss.pilon.all.fasta.all.maker.proteins.fasta.blast dromedary.pbjelly.pilon.abyss.pilon.all.fasta.all.maker.proteins.fasta.renamed.blast

## eighth rename the maker supplied gene names to ids made in the fifth step above

/opt/maker/bin/map_gff_ids dromedary.pbjelly.pilon.abyss.pilon.all.gff.id.map dromedary.pbjelly.pilon.abyss.pilon.all.renamed.gff

## ninth rename the maker supplied protein and transcript names to ids made in the fifth step above

/opt/maker/bin/map_fasta_ids dromedary.pbjelly.pilon.abyss.pilon.all.gff.id.map dromedary.pbjelly.pilon.abyss.pilon.all.fasta.all.maker.proteins.renamed.fasta

/opt/maker/bin/map_fasta_ids dromedary.pbjelly.pilon.abyss.pilon.all.gff.id.map dromedary.pbjelly.pilon.abyss.pilon.all.fasta.all.maker.transcripts.renamed.fasta

## tenth rename the maker supplied gene names in the BLAST search from sixth step above to ids made in the fifth step above

/opt/maker/bin/map_data_ids dromedary.pbjelly.pilon.abyss.pilon.all.gff.id.map dromedary.pbjelly.pilon.abyss.pilon.all.fasta.all.maker.proteins.fasta.renamed.blast

## eleventh annotate the genes

/opt/maker/bin/maker_functional_gff /genetics/elbers/maker/uniprot_trembl_release_2018_04.fasta dromedary.pbjelly.pilon.abyss.pilon.all.fasta.all.maker.proteins.fasta.renamed.blast dromedary.pbjelly.pilon.abyss.pilon.all.renamed.gff > dromedary.pbjelly.pilon.abyss.pilon.all.renamed.annotated.gff

## twelfth retain only annotations from "maker", which is a combination of annotations from genemark and augustus

awk -F"\t" -v OFS="\t" '$1=="##gff-version 3"||$2=="maker"' dromedary.pbjelly.pilon.abyss.pilon.all.renamed.annotated.gff > dromedary.pbjelly.pilon.abyss.pilon.all.renamed.annotated.maker.gff

## thirteenth retain only gene annotations that are high quality (AED < 0.50)

perl /opt/maker/src/quality_filter.pl -a 0.51 dromedary.pbjelly.pilon.abyss.pilon.all.renamed.annotated.maker.gff > dromedary.pbjelly.pilon.abyss.pilon.all.renamed.annotated.maker.qualityfilter.gff

## fourteenth make a gff file of just the genes (useful for IGV)

awk -F"\t" -v OFS="\t" '$1=="##gff-version 3"||$3=="gene"' dromedary.pbjelly.pilon.abyss.pilon.all.renamed.annotated.maker.qualityfilter.gff > dromedary.pbjelly.pilon.abyss.pilon.all.renamed.annotated.maker.qualityfilter.genes.only.gff

## fifteenth annotate the proteins and transcripts

/opt/maker/bin/maker_functional_fasta /genetics/elbers/maker/uniprot_trembl_release_2018_04.fasta dromedary.pbjelly.pilon.abyss.pilon.all.fasta.all.maker.proteins.fasta.renamed.blast dromedary.pbjelly.pilon.abyss.pilon.all.fasta.all.maker.proteins.renamed.fasta > dromedary.pbjelly.pilon.abyss.pilon.all.fasta.all.maker.proteins.renamed.annotated.fasta

/opt/maker/bin/maker_functional_fasta /genetics/elbers/maker/uniprot_trembl_release_2018_04.fasta dromedary.pbjelly.pilon.abyss.pilon.all.fasta.all.maker.transcripts.fasta.renamed.blast dromedary.pbjelly.pilon.abyss.pilon.all.fasta.all.maker.proteins.renamed.fasta > dromedary.pbjelly.pilon.abyss.pilon.all.fasta.all.maker.transcripts.renamed.annotated.fasta

#########################

# Step 24 annotate dromedary-pbjelly-racon-pilon assembly using second run of MAKER

#########################

cd /genetics/pacbio/maker-run2/

#####maker_opts.ctl#####

#-----Genome (these are always required)

genome=/genetics/pacbio/dromedary.pbjelly.pilon.abyss.pilon.chromosomes.fasta #genome sequence (fasta file or fasta embeded in GFF3 file)

organism_type=eukaryotic #eukaryotic or prokaryotic. Default is eukaryotic

#-----Re-annotation Using MAKER Derived GFF3

maker_gff= #MAKER derived GFF3 file

est_pass=0 #use ESTs in maker_gff: 1 = yes, 0 = no

altest_pass=0 #use alternate organism ESTs in maker_gff: 1 = yes, 0 = no

protein_pass=0 #use protein alignments in maker_gff: 1 = yes, 0 = no

rm_pass=0 #use repeats in maker_gff: 1 = yes, 0 = no

model_pass=0 #use gene models in maker_gff: 1 = yes, 0 = no

pred_pass=0 #use ab-initio predictions in maker_gff: 1 = yes, 0 = no

other_pass=0 #passthrough anyything else in maker_gff: 1 = yes, 0 = no

#-----EST Evidence (for best results provide a file for at least one)

est=/genetics/pacbio/dromedary.rnaseq.mapped.to.dromedary.pbjelly.pilon.abyss.pilon.chromosomes.fasta #set of ESTs or assembled mRNA-seq in fasta format

altest=/genetics/elbers/ragout/bactrian-rnaseq-reads-GCF_0007678551_Ca_bactrianus_MBC_10_genomic.fna.fa #EST/cDNA sequence file in fasta format from an alternate organism

est_gff= #aligned ESTs or mRNA-seq from an external GFF3 file

altest_gff= #aligned ESTs from a closly relate species in GFF3 format

#-----Protein Homology Evidence (for best results provide a file for at least one)

protein=/genetics/elbers/maker/ncbi-camelid-cow-old-maker-proteins.fa #protein sequence file in fasta format (i.e. from mutiple oransisms)

protein_gff= #aligned protein homology evidence from an external GFF3 file

#-----Repeat Masking (leave values blank to skip repeat masking)

model_org=all #select a model organism for RepBase masking in RepeatMasker

rmlib=/genetics/pacbio/maker-run1/dromedary-pbjelly-pilon-abyss-pilon-families.fa2 #provide an organism specific repeat library in fasta format for RepeatMasker

repeat_protein=/opt/maker/data/te_proteins.fasta #provide a fasta file of transposable element proteins for RepeatRunner

rm_gff= #pre-identified repeat elements from an external GFF3 file

prok_rm=0 #forces MAKER to repeatmask prokaryotes (no reason to change this), 1 = yes, 0 = no

softmask=1 #use soft-masking rather than hard-masking in BLAST (i.e. seg and dust filtering)

#-----Gene Prediction

snaphmm= #SNAP HMM file

gmhmm=/genetics/pacbio/maker-run1/dromedary-pbjelly-pilon-abyss-pilon-gmhmm.mod #GeneMark HMM file

augustus_species=dromedary-pbjelly-pilon-abyss-pilon-run2 #Augustus gene prediction species model

fgenesh_par_file= #FGENESH parameter file

pred_gff= #ab-initio predictions from an external GFF3 file

model_gff= #annotated gene models from an external GFF3 file (annotation pass-through)

est2genome=0 #infer gene predictions directly from ESTs, 1 = yes, 0 = no

protein2genome=0 #infer predictions from protein homology, 1 = yes, 0 = no

trna=0 #find tRNAs with tRNAscan, 1 = yes, 0 = no

snoscan_rrna= #rRNA file to have Snoscan find snoRNAs

unmask=0 #also run ab-initio prediction programs on unmasked sequence, 1 = yes, 0 = no

#-----Other Annotation Feature Types (features MAKER doesn't recognize)

other_gff= #extra features to pass-through to final MAKER generated GFF3 file

#-----External Application Behavior Options

alt_peptide=C #amino acid used to replace non-standard amino acids in BLAST databases

cpus=1 #max number of cpus to use in BLAST and RepeatMasker (not for MPI, leave 1 when using MPI)

#-----MAKER Behavior Options

max_dna_len=100000 #length for dividing up contigs into chunks (increases/decreases memory usage)

min_contig=10000 #skip genome contigs below this length (under 10kb are often useless)

pred_flank=200 #flank for extending evidence clusters sent to gene predictors

pred_stats=1 #report AED and QI statistics for all predictions as well as models

AED_threshold=1 #Maximum Annotation Edit Distance allowed (bound by 0 and 1)

min_protein=30 #require at least this many amino acids in predicted proteins

alt_splice=1 #Take extra steps to try and find alternative splicing, 1 = yes, 0 = no

always_complete=0 #extra steps to force start and stop codons, 1 = yes, 0 = no

map_forward=0 #map names and attributes forward from old GFF3 genes, 1 = yes, 0 = no

keep_preds=1 #Concordance threshold to add unsupported gene prediction (bound by 0 and 1)

split_hit=10000 #length for the splitting of hits (expected max intron size for evidence alignments)

single_exon=1 #consider single exon EST evidence when generating annotations, 1 = yes, 0 = no

single_length=250 #min length required for single exon ESTs if 'single_exon is enabled'

correct_est_fusion=0 #limits use of ESTs in annotation to avoid fusion genes

tries=2 #number of times to try a contig if there is a failure for some reason

clean_try=0 #remove all data from previous run before retrying, 1 = yes, 0 = no

clean_up=0 #removes theVoid directory with individual analysis files, 1 = yes, 0 = no

TMP= #specify a directory other than the system default temporary directory for temporary files

#####maker_opts.ctl#####

#####Note#####this is CamDro2

/opt/seqtk/seqtk randbase /genetics/pacbio/dromedary.pbjelly.pilon.abyss.pilon.chromosomes.fasta | /opt/seqtk/seqtk seq -U > dromedary.pbjelly.pilon.abyss.pilon.chromosomes.fasta

#####Note#####

mpiexec -n 75 /opt/maker/bin/maker -fix_nucleotides >> dromedary.pbjelly.pilon.abyss.pilon.chromosomes.fasta.output.txt 2>&1 &

## second get the GFF3 sequences

cd /genetics/pacbio/maker-run2/

/opt/maker/bin/gff3_merge -d dromedary.pbjelly.pilon.abyss.pilon.chromosomes.maker.output/dromedary.pbjelly.pilon.abyss.pilon.chromosomes_master_datastore_index.log -o dromedary.pbjelly.pilon.abyss.pilon.all2.gff -n

/opt/maker/bin/gff3_merge -d dromedary.pbjelly.pilon.abyss.pilon.chromosomes.maker.output/dromedary.pbjelly.pilon.abyss.pilon.chromosomes_master_datastore_index.log -o dromedary.pbjelly.pilon.abyss.pilon.all2.with.sequences.gff

## third get the FASTA sequences

/opt/maker/bin/fasta_merge -d dromedary.pbjelly.pilon.abyss.pilon.chromosomes.maker.output/dromedary.pbjelly.pilon.abyss.pilon.chromosomes_master_datastore_index.log -o dromedary.pbjelly.pilon.abyss.pilon.all2.fasta

## Determine the "best" cut-off value for AED (annotation edit distance)

wget https://raw.githubusercontent.com/mscampbell/Genome_annotation/master/AED_cdf_generator.pl

perl AED_cdf_generator.pl -b 0.025 dromedary.pbjelly.pilon.abyss.pilon.all2.gff > maker-run2.aed.cum.frac.below.txt

# AED 0.50 is "best" cut-off value as 0.392 of transcripts have AED <= 0.50

## fourth get transcript and protein sequences

### i get high quality proteins and transcripts

/opt/seqtk/seqtk seq -l0 dromedary.pbjelly.pilon.abyss.pilon.all2.fasta.all.maker.proteins.fasta | pcregrep -M " AED:0.[0-4][0-9].+\n\w+| AED:0.50.+\n\w+" \

> dromedary.pbjelly.pilon.abyss.pilon.all2.fasta.all.maker.proteins.0-0.50_AED.fasta

/opt/seqtk/seqtk seq -l0 dromedary.pbjelly.pilon.abyss.pilon.all2.fasta.all.maker.transcripts.fasta | pcregrep --buffer-size 3000000000 -M " AED:0.[0-4][0-9].+\n\w+| AED:0.50.+\n\w+" \

> dromedary.pbjelly.pilon.abyss.pilon.all2.fasta.all.maker.transcripts.0-0.50_AED.fasta

## ii run ideel

mkdir ideel

/opt/diamond/diamond_0.9.19 blastp --threads 75 --max-target-seqs 1 --db /genetics/elbers/maker/uniprot_trembl_release_2018_04.fasta \

--query dromedary.pbjelly.pilon.abyss.pilon.all2.fasta.all.maker.proteins.0-0.50_AED.fasta \

--outfmt 6 qlen slen --out ideel/dromedary.pbjelly.pilon.abyss.pilon.all2.fasta.all.maker.proteins.0-0.50_AED.fasta.against.uniprot_trembl_release_2018_04.fasta.blast > diamond.log 2>&1

echo -e 'test <- read.table("ideel/dromedary.pbjelly.pilon.abyss.pilon.all2.fasta.all.maker.proteins.0-0.50_AED.fasta.against.uniprot_trembl_release_2018_04.fasta.blast")\ntest2 <- test$V1/test$V2\ntest3 <- test2[test2 >= 0.85]\ntest4 <- test3[test3 <= 1.15]\ntest5 <- test2[test2 < 0.85]\ncat("\n")\npaste("There are",length(test2),"protein hits, and", length(test4), round(length(test4)/length(test2)*100,2), "%","are between 0.85 and 1.15 (querylength/subjectlength).")\ncat("\n")\npaste("There are", length(test5), round(length(test5)/length(test2)*100,2),"%", "protein hits less than 0.85 (querylength/subjectlength).")\ncat("\n")'> stats.R

Rscript stats.R

# "There are 32415 protein hits, and 11478 35.41 % are between 0.85 and 1.15 (querylength/subjectlength)."

# "There are 11806 36.42 % protein hits less than 0.85 (querylength/subjectlength)."

## fifth make a id map for all genes in gff file (i.e., instead of maker-gene-01124356 convert to Cadr-000000001)

/opt/maker/bin/maker_map_ids --prefix Cadr_ --justify 8 dromedary.pbjelly.pilon.abyss.pilon.all2.gff > dromedary.pbjelly.pilon.abyss.pilon.all2.gff.id.map

## sixth blast high quality maker proteins against uniprot trembl

/opt/diamond/diamond_0.9.19 blastp --threads 75 --max-target-seqs 1 --db /genetics/elbers/maker/uniprot_trembl_release_2018_04.fasta \

--evalue 1e-6 --query dromedary.pbjelly.pilon.abyss.pilon.all2.fasta.all.maker.proteins.0-0.50_AED.fasta --outfmt 6 \

--out dromedary.pbjelly.pilon.abyss.pilon.all2.fasta.all.maker.proteins.fasta.blast > diamond.maker.log 2>&1

## seventh make copies of files (because scripts below overwrite input files)

cp dromedary.pbjelly.pilon.abyss.pilon.all2.gff dromedary.pbjelly.pilon.abyss.pilon.all2.renamed.gff

cp dromedary.pbjelly.pilon.abyss.pilon.all2.fasta.all.maker.proteins.0-0.50_AED.fasta dromedary.pbjelly.pilon.abyss.pilon.all2.fasta.all.maker.proteins.renamed.fasta

cp dromedary.pbjelly.pilon.abyss.pilon.all2.fasta.all.maker.transcripts.0-0.50_AED.fasta dromedary.pbjelly.pilon.abyss.pilon.all2.fasta.all.maker.transcripts.renamed.fasta

cp dromedary.pbjelly.pilon.abyss.pilon.all2.fasta.all.maker.proteins.fasta.blast dromedary.pbjelly.pilon.abyss.pilon.all2.fasta.all.maker.proteins.fasta.renamed.blast

## eighth rename the maker supplied gene names to ids made in the fifth step above

/opt/maker/bin/map_gff_ids dromedary.pbjelly.pilon.abyss.pilon.all2.gff.id.map dromedary.pbjelly.pilon.abyss.pilon.all2.renamed.gff

## ninth rename the maker supplied protein and transcript names to ids made in the fifth step above

/opt/maker/bin/map_fasta_ids dromedary.pbjelly.pilon.abyss.pilon.all2.gff.id.map dromedary.pbjelly.pilon.abyss.pilon.all2.fasta.all.maker.proteins.renamed.fasta

/opt/maker/bin/map_fasta_ids dromedary.pbjelly.pilon.abyss.pilon.all2.gff.id.map dromedary.pbjelly.pilon.abyss.pilon.all2.fasta.all.maker.transcripts.renamed.fasta

## tenth rename the maker supplied gene names in the BLAST search from sixth step above to ids made in the fifth step above

/opt/maker/bin/map_data_ids dromedary.pbjelly.pilon.abyss.pilon.all2.gff.id.map dromedary.pbjelly.pilon.abyss.pilon.all2.fasta.all.maker.proteins.fasta.renamed.blast

## eleventh annotate the genes

/opt/maker/bin/maker_functional_gff /genetics/elbers/maker/uniprot_trembl_release_2018_04.fasta dromedary.pbjelly.pilon.abyss.pilon.all2.fasta.all.maker.proteins.fasta.renamed.blast dromedary.pbjelly.pilon.abyss.pilon.all2.renamed.gff > dromedary.pbjelly.pilon.abyss.pilon.all2.renamed.annotated.gff

## twelfth retain only annotations from "maker", which is a combination of annotations from genemark and augustus

awk -F"\t" -v OFS="\t" '$1=="##gff-version 3"||$2=="maker"' dromedary.pbjelly.pilon.abyss.pilon.all2.renamed.annotated.gff > dromedary.pbjelly.pilon.abyss.pilon.all2.renamed.annotated.maker.gff

## thirteenth retain only gene annotations that are high quality (AED <= 0.50)

perl /opt/maker/src/quality_filter.pl -a 0.51 dromedary.pbjelly.pilon.abyss.pilon.all2.renamed.annotated.maker.gff > dromedary.pbjelly.pilon.abyss.pilon.all2.renamed.annotated.maker.qualityfilter.gff

## fourteenth make a gff file of just the genes (useful for IGV)

awk -F"\t" -v OFS="\t" '$1=="##gff-version 3"||$3=="gene"' dromedary.pbjelly.pilon.abyss.pilon.all2.renamed.annotated.maker.qualityfilter.gff > dromedary.pbjelly.pilon.abyss.pilon.all2.renamed.annotated.maker.qualityfilter.genes.only.gff

## fifteenth annotate the proteins and transcripts

/opt/maker/bin/maker_functional_fasta /genetics/elbers/maker/uniprot_trembl_release_2018_04.fasta dromedary.pbjelly.pilon.abyss.pilon.all2.fasta.all.maker.proteins.fasta.renamed.blast dromedary.pbjelly.pilon.abyss.pilon.all2.fasta.all.maker.proteins.renamed.fasta > dromedary.pbjelly.pilon.abyss.pilon.all2.fasta.all.maker.proteins.renamed.annotated.fasta

/opt/maker/bin/maker_functional_fasta /genetics/elbers/maker/uniprot_trembl_release_2018_04.fasta dromedary.pbjelly.pilon.abyss.pilon.all2.fasta.all.maker.proteins.fasta.renamed.blast dromedary.pbjelly.pilon.abyss.pilon.all2.fasta.all.maker.transcripts.renamed.fasta > dromedary.pbjelly.pilon.abyss.pilon.all2.fasta.all.maker.transcripts.renamed.annotated.fasta

#########################

# Step 25 Table S6 - Average sensitivity and specificity (in parentheses) for the Augustus ab initio models used during the first and second MAKER runs tested against 75 sets of 250 randomly chosen transcripts with annotation edit distance ≤ 0.25

#########################

## first Generate training.gb using autoAug (you don't need to run the whole pipeline - i.e., don't need to run optimize augustus, just using it to generate training.gb file)

cd /genetics/pacbio/maker-run1

/opt/seqtk/seqtk seq -l0 dromedary.pbjelly.pilon.abyss.pilon.all.fasta.all.maker.transcripts.fasta |pcregrep --buffer-size 3000000000 -M " AED:0.[0-1][0-9].+\n\w+| AED:0.[2][0-5].+\n\w+" > dromedary.pbjelly.pilon.abyss.pilon.all.fasta.all.maker.transcripts.0-0.25_AED.fasta

perl -pe "s/(^>.+)\stranscript.+\n/\1\n/" dromedary.pbjelly.pilon.abyss.pilon.all.fasta.all.maker.transcripts.0-0.25_AED.fasta > dromedary.pbjelly.pilon.abyss.pilon.all.maker.transcripts.cdna.for.augustus.training.0-0.25_AED.fasta

## make augustus input

perl /opt/maker/src/quality_filter.pl -a 0.25 dromedary.pbjelly.pilon.abyss.pilon.all.renamed.annotated.maker.gff |awk -F"\t" -v OFS="\t" '$1=="##gff-version 3"||$3=="CDS"' > dromedary.pbjelly.pilon.abyss.pilon.all.maker.0-0.25_AED.input.for.autoAug.gff

# autoAug-pblat uses pblat (https://github.com/icebert/pblat with 75 cores) instead of blat in line 671 of autoAug.pl

/genetics/elbers/augustus/scripts/autoAug-pblat.pl \

--genome=/genetics/pacbio/dromedary.pbjelly.pilon.abyss.pilon.chromosomes.fasta \

--species=dromedary-pbjelly-pilon-abyss-pilon-run2-AED-25 \

--cdna=dromedary.pbjelly.pilon.abyss.pilon.all.maker.transcripts.cdna.for.augustus.training.0-0.25_AED.fasta \

--trainingset=dromedary.pbjelly.pilon.abyss.pilon.all.maker.0-0.25_AED.input.for.autoAug.gff \

-v -v –v --singleCPU \

--noninteractive > etraining.log 2>&1 &

## second Copy training.gb into another folder

cd /genetics/pacbio/maker-run1/train-augustus/test

cp /genetics/pacbio/maker-run1/autoAug/autoAugTrain/training/training.gb .

## third Generate 75 sets of 250 random transcripts with AED <= 0.25

# modified /opt/maker/exe/augustus/scripts/randomSplit.pl "srand 4" "srand(time)" (i.e., line 18)

# new script is /genetics/elbers/augustus/scripts/randomSplit.pl

for i in $(seq 1 75); do

/genetics/elbers/augustus/scripts/randomSplit.pl training.gb 250

mv training.gb.test training.gb.test${i}

rm training.gb.train

done &

parallel --no-notice '/opt/maker/exe/augustus/bin/augustus --species=BUSCO_dromedary-pbjelly-pilon-abyss-pilon training.gb.test{} | tee BUSCO_dromedary-pbjelly-pilon-abyss-pilon-{}.out' ::: {1..75}

parallel --no-notice '/opt/maker/exe/augustus/bin/augustus --species=dromedary-pbjelly-pilon-abyss-pilon-run2 training.gb.test{} | tee dromedary-pbjelly-pilon-abyss-pilon-run2-{}.out' ::: {1..75}

## fourth Assess the average level of sensitivity and specificity at the gene-,exon-, and nucleotide-levels

# average BUSCO gene-level sensitivity

grep "^gene level" BUSCO*.out |cut -d "|" -f 7 |awk '{sum+=$1}END{print sum/75}'

# 0.251787

# average BUSCO gene-level specificity

grep "^gene level" BUSCO*.out |cut -d "|" -f 8 | awk '{sum+=$1}END{print sum/75}'

# 0.235707

# average BUSCO exon-level sensitivity

grep "^exon level" BUSCO*.out |cut -d "|" -f 7 |awk '{sum+=$1}END{print sum/75}'

# 0.726853

# average BUSCO exon-level specificity

grep "^exon level" BUSCO*.out |cut -d "|" -f 8 |awk '{sum+=$1}END{print sum/75}'

# 0.742147

# average BUSCO nucleotide-level sensitivity

grep "^nucleotide level" BUSCO*.out |cut -d "|" -f 2 |awk '{sum+=$1}END{print sum/75}'

# 0.864827

# average BUSCO nucleotide-level specificity

grep "^nucleotide level" BUSCO*.out |cut -d "|" -f 3 |awk '{sum+=$1}END{print sum/75}'

# 0.897267

# average autoAug.pl gene-level sensitivity

grep "^gene level" drom*.out |cut -d "|" -f 7 |awk '{sum+=$1}END{print sum/75}'

0.0818667

# average autoAug.pl gene-level specificity

grep "^gene level" drom*.out |cut -d "|" -f 8 | awk '{sum+=$1}END{print sum/75}'

0.046328

# average autoAug.pl exon-level sensitivity

grep "^exon level" drom*.out |cut -d "|" -f 7 |awk '{sum+=$1}END{print sum/75}'

0.513027

# average autoAug.pl exon-level specificity

grep "^exon level" drom*.out |cut -d "|" -f 8 |awk '{sum+=$1}END{print sum/75}'

0.3886

# average autoAug.pl nucleotide-level sensitivity

grep "^nucleotide level" drom*.out |cut -d "|" -f 2 |awk '{sum+=$1}END{print sum/75}'

0.81088

# average autoAug.pl nucleotide-level specificity

grep "^nucleotide level" drom*.out |cut -d "|" -f 3 |awk '{sum+=$1}END{print sum/75}'

0.54264

## fifth

# the BUSCO ab initio Augustus model has higher sensitivity and specificity than the autoAug.pl model (used

# the second round of MAKER, hence the worse gene models

#########################

# Step 26 Figure 4 - Frequency polygon of query sequence length (predicted proteins) divided by subject (UniProt/Trembl) sequence length

#########################

####Rscript for plotting#####

library(ggplot2)

setwd("C:/Users/elbersj/Dropbox/camel_postdoc/manuscripts/dromedary-assembly/protein-truncatedness/")

df1<-read.table("fitak-old-maker.proteins.fasta.against.uniprot_trembl_release_2018_04.blast")

df2<-read.table("dromedary.pbjelly.pilon.abyss.pilon.all.fasta.all.maker.proteins.0-0.50_AED.fasta.against.uniprot_trembl_release_2018_04.fasta.blast")

df3<-read.table("dromedary.pbjelly.pilon.abyss.pilon.all2.fasta.all.maker.proteins.0-0.50_AED.fasta.against.uniprot_trembl_release_2018_04.fasta.blast")

test1 <-(df1$V1/df1$V2)

test2 <-(df2$V1/df2$V2)

test3 <-(df3$V1/df3$V2)

test1 <- test1[test1 < 2]

test2 <- test2[test2 < 2]

test3 <- test3[test3 < 2]

test4 <- c(test1,test2,test3)

set.seed(1)

df <- data.frame("scores"=test4,

"exp"=c(rep("CamDro1",length(test1)),rep("CamDro2 MAKER run 1",length(test2)),rep("CamDro2 MAKER run 2",length(test3))))

plot2 <- ggplot(df, aes(scores, stat(density), color=exp)) +

geom_freqpoly(binwidth=0.05, size=0.9) +

theme_bw() +

theme(panel.grid.major.x = element_blank(),

panel.grid.minor.x = element_blank(),

panel.grid.major.y = element_blank(),

panel.grid.minor.y = element_blank(),

panel.border = element_blank(),

legend.text=element_text(size=12),

axis.line = element_line(color="black"),

legend.position = c(0.2,0.85),

legend.title = element_blank(),

axis.text = element_text(size=12),

axis.title = element_text(size=14)) +

ylab("Density") +

xlab("Query sequence length / Subject sequence length")

plot2

ggsave(filename = "protrein-truncatedness.png",device = "png", height = 6 , width = 6, dpi = 600,plot = plot2)

#########################

# Step 27 Figure 5 - Cumulative proportion of transcripts with specific or lower annotation edit distance (AED) for each MAKER run.

#########################

#####Rscript for plotting

library(ggplot2)

setwd("C:/Users/elbersj/Dropbox/camel_postdoc/manuscripts/dromedary-assembly/figures-scripts-instructions-for-CamDro2/")

df <- read.table("C:/Users/elbersj/Dropbox/camel_postdoc/manuscripts/dromedary-assembly/aed-cumulative-fraction-lower.txt",header=T,sep="\t")

plot2 <- ggplot(df, aes(AED, frac)) +

geom_line(aes(linetype=run), size=1.2) +

geom_vline(xintercept=0.5, size=0.8, color="gray") +

theme_bw() +

theme(panel.grid.major.x = element_blank(),

panel.grid.minor.x = element_blank(),

panel.grid.major.y = element_blank(),

panel.grid.minor.y = element_blank(),

panel.border = element_blank(),

axis.line = element_line(color="black"),

legend.position = c(0.2,0.85),

legend.title = element_blank(),

axis.text = element_text(size=10)) +

ylab("Cumulative Proportion of Transcripts with Specified AED Value") +

xlab("Annotation Edit Distance (AED)")

ggsave(filename = "aed-plot.png",device = "png", height = 6 , width = 6, dpi = 600,plot = plot2)

#####aed-cumulative-fraction-lower.txt

AED frac run

0.000 0.008 MAKER run 1

0.025 0.028 MAKER run 1

0.050 0.058 MAKER run 1

0.075 0.081 MAKER run 1

0.100 0.114 MAKER run 1

0.125 0.138 MAKER run 1

0.150 0.176 MAKER run 1

0.175 0.202 MAKER run 1

0.200 0.244 MAKER run 1

0.225 0.275 MAKER run 1

0.250 0.322 MAKER run 1

0.275 0.355 MAKER run 1

0.300 0.405 MAKER run 1

0.325 0.441 MAKER run 1

0.350 0.499 MAKER run 1

0.375 0.539 MAKER run 1

0.400 0.599 MAKER run 1

0.425 0.640 MAKER run 1

0.450 0.702 MAKER run 1

0.475 0.739 MAKER run 1

0.500 0.784 MAKER run 1

0.525 0.796 MAKER run 1

0.550 0.810 MAKER run 1

0.575 0.817 MAKER run 1

0.600 0.826 MAKER run 1

0.625 0.831 MAKER run 1

0.650 0.840 MAKER run 1

0.675 0.845 MAKER run 1

0.700 0.852 MAKER run 1

0.725 0.856 MAKER run 1

0.750 0.863 MAKER run 1

0.775 0.868 MAKER run 1

0.800 0.875 MAKER run 1

0.825 0.879 MAKER run 1

0.850 0.886 MAKER run 1

0.875 0.890 MAKER run 1

0.900 0.897 MAKER run 1

0.925 0.900 MAKER run 1

0.950 0.906 MAKER run 1

0.975 0.909 MAKER run 1

1.000 1.000 MAKER run 1

0.000 0.003 MAKER run 2

0.025 0.009 MAKER run 2

0.050 0.020 MAKER run 2

0.075 0.028 MAKER run 2

0.100 0.041 MAKER run 2

0.125 0.051 MAKER run 2

0.150 0.066 MAKER run 2

0.175 0.077 MAKER run 2

0.200 0.095 MAKER run 2

0.225 0.108 MAKER run 2

0.250 0.129 MAKER run 2

0.275 0.144 MAKER run 2

0.300 0.168 MAKER run 2

0.325 0.185 MAKER run 2

0.350 0.213 MAKER run 2

0.375 0.233 MAKER run 2

0.400 0.265 MAKER run 2

0.425 0.287 MAKER run 2

0.450 0.324 MAKER run 2

0.475 0.352 MAKER run 2

0.500 0.392 MAKER run 2

0.525 0.405 MAKER run 2

0.550 0.422 MAKER run 2

0.575 0.432 MAKER run 2

0.600 0.447 MAKER run 2

0.625 0.457 MAKER run 2

0.650 0.470 MAKER run 2

0.675 0.479 MAKER run 2

0.700 0.492 MAKER run 2

0.725 0.500 MAKER run 2

0.750 0.514 MAKER run 2

0.775 0.523 MAKER run 2

0.800 0.536 MAKER run 2

0.825 0.546 MAKER run 2

0.850 0.559 MAKER run 2

0.875 0.567 MAKER run 2

0.900 0.580 MAKER run 2

0.925 0.588 MAKER run 2

0.950 0.597 MAKER run 2

0.975 0.602 MAKER run 2

1.000 1.000 MAKER run 2

#########################

# Step 28 Adding chromosome 36 to annotation and calculating the number of RH probes that map to each super scaffold

#########################

cd /genetics/pacbio

wget http://genome-test.cse.ucsc.edu/~kent/exe/linux/isPcr33.zip

unzip isPcr33.zip

#####beginning of query2-36#####

36q12-q13 CCAGATTGCCAGCTAGAGGT ATTGGGGCATTCAATACAGC

36q12-q13 GATGCGCCATCTTAGTTCCT GGAGACTGTCATTCATGCTGTT

36q12-q13 AGATGGGAAGCACAGAGCAC CCTTAGGGCGGTTAGGAAAG

36q12-q13 AATGAGGTAAACACTGGTAATTGTG CGCCTTAATTAGGCAGCAGA

36q12-q13 ACCCTGGGGAGCCATTAG CTCATTTCCGGAAGGACAGA

36q12-q13 CCCAACTGCTTCGCAGAGA CTACATCTGCCTGCATTCGT

36q12-q13 CCTGGTGTTCCAGAACTTCG GTACTTCAGCGGCAGCTTTC

#####end of query2-36#####

./isPcr dromedary.pbjelly.pilon.abyss.pilon.chromosomes.fasta query2-36 output2-36

cp output2-36 rhmarkers/chr36rhmarkers.txt

# Assign with BLASTn

## second perform blast, keeping only the first best hit with evalue of at least 1e-30

seq -w 1 36 > rhmarkers/samples

echo "X" >> rhmarkers/samples

/usr/bin/makeblastdb -dbtype nucl -in dromedary.pbjelly.pilon.abyss.pilon.chromosomes.fasta

while read i;do

## third blast the markers for each chromosome

/usr/bin/blastn -num_threads 75 -db dromedary.pbjelly.pilon.abyss.pilon.chromosomes.fasta -query rhmarkers/chr${i}rhmarkers.txt -outfmt 6 -evalue 1e-30 -max_hsps 1 > rhmarkers/chr${i}rhmarkers.txt.blast

## fourth count the BLAST hits for contigs/scaffolds

echo ${i} >> contigs-to-chromosomes.txt

cut -f 1-2 rhmarkers/chr${i}rhmarkers.txt.blast |awk '!seen[$1]++'|cut -f 2 |sort |uniq -c|sort -n |perl -pe "s/( )+/\t/g" |perl -pe "s/^\t//g" > chr-${i}.blast

cut -f 1-2 rhmarkers/chr${i}rhmarkers.txt.blast |awk '!seen[$1]++'|cut -f 2 |sort |uniq -c|sort -n |tail -n 1|perl -pe "s/( )+/\t/g" |perl -pe "s/^\t//g" |cut -f 2 >> contigs-to-chromosomes.txt

done < rhmarkers/samples

while read i;do

echo ${i} >> blast.results

tail -n 1 chr-${i}.blast|cut -f 1 >> blast.results

cat chr-${i}.blast |awk '{sum+=$1}END{print sum}' >> blast.results

grep -c ">" rhmarkers/chr${i}rhmarkers.txt >> blast.results

done < rhmarkers/samples

cat blast.results |paste - - - - > blast.results2

## blast.results2 is Table S4 (without the percentages)

## fifth modify numbers less than 9

perl -pi -e "s/^0//g" contigs-to-chromosomes.txt

## sixth make a copy of pilon assembly and rename it

/opt/seqtk/seqtk seq -l80 dromedary.pbjelly.pilon.abyss.pilon.chromosomes.fasta > tmp2 && mv tmp2 dromedary.pbjelly.pilon.abyss.pilon.chromosomes.fasta

## seventh rename the contigs to chromosome names (takes about 1 hour)

cat contigs-to-chromosomes.txt | while read -r ONE;do

read -r TWO

perl -pi -e "s/>${TWO}\n/>${ONE}\n/" dromedary.pbjelly.pilon.abyss.pilon.chromosomes.fasta

done

## eigth sort the chromosomes and contigs by number (ex: 1,2,3,4,X,Contig200,Contig201),

## then output 60 bases per line, then make all bases uppercase (no soft-masking)

cat dromedary.pbjelly.pilon.abyss.pilon.chromosomes.fasta | /opt/seqtk/seqtk seq -l0 | \

paste - - |grep "Contig" > contigs

cat dromedary.pbjelly.pilon.abyss.pilon.chromosomes.fasta | /opt/seqtk/seqtk seq -l0 | \

paste - - |grep -v "Contig" |grep -v "X" > chromosomes

cat dromedary.pbjelly.pilon.abyss.pilon.chromosomes.fasta | /opt/seqtk/seqtk seq -l0 | \

paste - - |grep "X" > Xchromosome

cat chromosomes |sort -k 1.2 -n > tmp2 && mv tmp2 chromosomes

cat contigs |sort -k 1.8 -n > tmp2 && mv tmp2 contigs

cat chromosomes Xchromosome contigs | tr "\t" "\n" |/opt/seqtk/seqtk seq -l60 -U > tmp2

mv tmp2 dromedary.pbjelly.pilon.abyss.pilon.chromosomes.fasta

cat chromosomes Xchromosome | tr "\t" "\n" |/opt/seqtk/seqtk seq -l60 -U > dromedary.pbjelly.pilon.abyss.pilon.chromosomes.chromosomes.only.fasta

cat contigs | tr "\t" "\n" |/opt/seqtk/seqtk seq -l60 -U > dromedary.pbjelly.pilon.abyss.pilon.chromosomes.unscaffolded.contigs.only.fasta

### how many bases are in chromosomes

samtools faidx dromedary.pbjelly.pilon.abyss.pilon.chromosomes.chromosomes.only.fasta

awk '{sum+=$2} END {print sum}' dromedary.pbjelly.pilon.abyss.pilon.chromosomes.chromosomes.only.fasta.fai

#2052758708

### how many bases are in scaffolds not assigned to chromosomes

samtools faidx dromedary.pbjelly.pilon.abyss.pilon.chromosomes.unscaffolded.contigs.only.fasta

awk '{sum+=$2} END {print sum}' dromedary.pbjelly.pilon.abyss.pilon.chromosomes.unscaffolded.contigs.only.fasta.fai

#101628251

### what proportion of bases are not assigned to chromosomes

#101628251/(101628251+2052758708) = 0.04717

#########################

# Step 29 Updating MAKER GFF files for chromosome 36 and sorting them

#########################

cd /genetics/elbers

git clone https://github.com/billzt/gff3sort.git

cd /genetics/pacbio/maker-run1/

awk '{if ($1=="Contig534") print 36,$2,$3,$4,$5,$6,$7,$8,$9; else print $1,$2,$3,$4,$5,$6,$7,$8,$9;}' OFS='\t' FS='\t' dromedary.pbjelly.pilon.abyss.pilon.all.renamed.annotated.maker.qualityfilter.genes.only.gff > tmp2 && mv tmp2 dromedary.pbjelly.pilon.abyss.pilon.all.renamed.annotated.maker.qualityfilter.genes.only.gff

awk '{if ($1=="Contig534") print 36,$2,$3,$4,$5,$6,$7,$8,$9; else print $1,$2,$3,$4,$5,$6,$7,$8,$9;}' OFS='\t' FS='\t' dromedary.pbjelly.pilon.abyss.pilon.all.renamed.annotated.maker.qualityfilter.gff > tmp2 && mv tmp2 dromedary.pbjelly.pilon.abyss.pilon.all.renamed.annotated.maker.qualityfilter.gff

perl /genetics/elbers/gff3sort/gff3sort.pl --precise --chr_order natural dromedary.pbjelly.pilon.abyss.pilon.all.renamed.annotated.maker.qualityfilter.genes.only.gff > tmp2 && mv tmp2 dromedary.pbjelly.pilon.abyss.pilon.all.renamed.annotated.maker.qualityfilter.genes.only.gff

perl /genetics/elbers/gff3sort/gff3sort.pl --precise --chr_order natural dromedary.pbjelly.pilon.abyss.pilon.all.renamed.annotated.maker.qualityfilter.gff > tmp2 && mv tmp2 dromedary.pbjelly.pilon.abyss.pilon.all.renamed.annotated.maker.qualityfilter.gff

#########################

# Step 30 Submit genome annotations to NCBI

#########################

cd /genetics/pacbio/maker-run1

## Note, you can create template.sbt by going here:

## https://submit.ncbi.nlm.nih.gov/genbank/template/submission/

#####beginning of template.sbt#####

Submit-block ::= {

contact {

contact {

name name {

last "Elbers",

first "Jean",

middle "",

initials "",

suffix "",

title ""

},

affil std {

affil "Vetmeduni Vienna",

div "Research Institute of Wildlife Ecology",

city "Vienna",

country "Austria",

street "Savoyenstrasse 1",

email "jean.elbers@gmail.com",

postal-code "1160"

}

}

},

cit {

authors {

names std {

{

name name {

last "Elbers",

first "Jean",

middle "",

initials "P.",

suffix "",

title ""

}

},

{

name name {

last "Rogers",

first "Mark",

middle "",

initials "F.",

suffix "",

title ""

}

},

{

name name {

last "Perelman",

first "Polina",

middle "",

initials "L.",

suffix "",

title ""

}

},

{

name name {

last "Proskuryakova",

first "Anastasia",

middle "",

initials "A.",

suffix "",

title ""

}

},

{

name name {

last "Serdyukova",

first "Natalia",

middle "",

initials "A.",

suffix "",

title ""

}

},

{

name name {

last "Johnson",

first "Warren",

middle "",

initials "E.",

suffix "",

title ""

}

},

{

name name {

last "Horin",

first "Petr",

middle "",

initials "",

suffix "",

title ""

}

},

{

name name {

last "Corander",

first "Jukka",

middle "",

initials "",

suffix "",

title ""

}

},

{

name name {

last "Murphy",

first "David",

middle "",

initials "",

suffix "",

title ""

}

},

{

name name {

last "Burger",

first "Pamela",

middle "",

initials "A.",

suffix "",

title ""

}

}

},

affil std {

affil "Vetmeduni Vienna",

div "Research Institute of Wildlife Ecology",

city "Vienna",

country "Austria",

street "Savoyenstrasse 1",

postal-code "1160"

}

}

},

subtype new

}

Seqdesc ::= pub {

pub {

gen {

cit "unpublished",

authors {

names std {

{

name name {

last "Elbers",

first "Jean",

middle "",

initials "P.",

suffix "",

title ""

}

},

{

name name {

last "Rogers",

first "Mark",

middle "",

initials "F.",

suffix "",

title ""

}

},

{

name name {

last "Perelman",

first "Polina",

middle "",

initials "L.",

suffix "",

title ""

}

},

{

name name {

last "Proskuryakova",

first "Anastasia",

middle "",

initials "A.",

suffix "",

title ""

}

},

{

name name {

last "Serdyukova",

first "Natalia",

middle "",

initials "A.",

suffix "",

title ""

}

},

{

name name {

last "Johnson",

first "Warren",

middle "",

initials "E.",

suffix "",

title ""

}

},

{

name name {

last "Horin",

first "Petr",

middle "",

initials "",

suffix "",

title ""

}

},

{

name name {

last "Corander",

first "Jukka",

middle "",

initials "",

suffix "",

title ""

}

},

{

name name {

last "Murphy",

first "David",

middle "",

initials "",

suffix "",

title ""

}

},

{

name name {

last "Burger",

first "Pamela",

middle "",

initials "A.",

suffix "",

title ""

}

}

}

},

title "Improving Illumina assemblies with Hi-C and long reads: an

example with the North African dromedary"

}

}

}

Seqdesc ::= user {

type str "DBLink",

data {

{

label str "BioProject",

num 1,

data strs {

"PRJNA269274"

}

},

{

label str "BioSample",

num 1,

data strs {

"SAMN03252735"

}

}

}

}

Seqdesc ::= user {

type str "Submission",

data {

{

label str "AdditionalComment",

data str "ALT EMAIL:jean.elbers@gmail.com"

}

}

}

Seqdesc ::= user {

type str "Submission",

data {

{

label str "AdditionalComment",

data str "Submission Title:None"

}

}

}

#####end of template.sbt#####

# downloaded GAG on Dec 6 2018

cd /genetics/elbers

wget https://github.com/genomeannotation/GAG/tarball/master

tar xzf master

wget ftp://ftp.ncbi.nih.gov/toolbox/ncbi_tools/converters/by_program/tbl2asn/linux64.tbl2asn.gz

gunzip linux64.tbl2asn.gz

chmod u+x linux64.tbl2asn

cd /genetics/pacbio/maker-run1/

cat dromedary.pbjelly.pilon.abyss.pilon.all.renamed.annotated.maker.qualityfilter.gff|\

perl -pe "s/Name=\w+_\w+;//g" |\

perl -pe "s/Name=\w+_\w+-R\w;//g" |\

perl -pe "s/Note=Similar to /Name=/g"|\

perl -pe "s/Note=Protein of unknown function/Name=hypothetical protein/g"|\

perl -pe "s/(Name=\w+):.+/\1;/xg" | \

perl -pe "s/(Name=.+)\(.+/\1;/xg" |grep -Pv "\w+_prime_UTR" > CamDro2_for_gag.gff

python2 /genetics/elbers/genomeannotation-GAG-997e384/gag.py \

--fasta ../dromedary.pbjelly.pilon.abyss.pilon.chromosomes.fasta \

--gff CamDro2_for_gag.gff \

--fix_start_stop \

--fix_terminal_ns \

--out gag_output > gag.1.log 2>&1

cd gag_output

wget http://kirill-kryukov.com/study/tools/fasta-splitter/files/fasta-splitter-0.2.6.zip

unzip fasta-splitter-0.2.6.zip

perl fasta-splitter.pl --n-parts 2 genome.fasta --out-dir ./

mkdir tbl2asn1

mkdir tbl2asn2

mv genome.part-1.fasta tbl2asn1/genome.part-1.fsa

mv genome.part-2.fasta tbl2asn2/genome.part-2.fsa

cp genome.tbl tbl2asn1/genome.part-1.tbl

cp genome.tbl tbl2asn2/genome.part-2.tbl

cd tbl2asn1

/genetics/elbers/linux64.tbl2asn -t ../../template.sbt -i ./genome.part-1.fsa -M b -Z discrep -a r10u1000 -j "[organism=Camelus dromedarius]" -V v > tbl2asn-1.log 2>&1

cd ../tbl2asn2

/genetics/elbers/linux64.tbl2asn -t ../../template.sbt -i ./genome.part-2.fsa -M b -Z discrep -a r10u1000 -j "[organism=Camelus dromedarius]" -V v > tbl2asn-2.log 2>&1

mv tbl2asn1/genome.part-1.sqn ../dromedary.pbjelly.pilon.abyss.pilon.chromosomes.part-1.sqn

mv tbl2asn2/genome.part-2.sqn ../dromedary.pbjelly.pilon.abyss.pilon.chromosomes.part-2.sqn

cd ..

pigz dromedary.pbjelly.pilon.abyss.pilon.chromosomes.part-?.sqn

# Submitted to NCBI

## dromedary.pbjelly.pilon.abyss.pilon.chromosomes.part-1.sqn.gz

## dromedary.pbjelly.pilon.abyss.pilon.chromosomes.part-2.sqn.gz

#########################

# Step 31 Annotate CamDro1 with same input and settings as CamDro2

#########################

cd /genetics/elbers/CamDro1/maker

#####maker_opts.ctl#####

#-----Genome (these are always required)

genome=CamDro1.fa #genome sequence (fasta file or fasta embeded in GFF3 file)

organism_type=eukaryotic #eukaryotic or prokaryotic. Default is eukaryotic

#-----Re-annotation Using MAKER Derived GFF3

maker_gff= #MAKER derived GFF3 file

est_pass=0 #use ESTs in maker_gff: 1 = yes, 0 = no

altest_pass=0 #use alternate organism ESTs in maker_gff: 1 = yes, 0 = no

protein_pass=0 #use protein alignments in maker_gff: 1 = yes, 0 = no

rm_pass=0 #use repeats in maker_gff: 1 = yes, 0 = no

model_pass=0 #use gene models in maker_gff: 1 = yes, 0 = no

pred_pass=0 #use ab-initio predictions in maker_gff: 1 = yes, 0 = no

other_pass=0 #passthrough anyything else in maker_gff: 1 = yes, 0 = no

#-----EST Evidence (for best results provide a file for at least one)

est=dromedary.rnaseq.mapped.to.dromedary.pbjelly.pilon.abyss.pilon.chromosomes.fasta #set of ESTs or assembled mRNA-seq in fasta format

altest=bactrian-rnaseq-reads-GCF_0007678551_Ca_bactrianus_MBC_10_genomic.fna.fa #EST/cDNA sequence file in fasta format from an alternate organism

est_gff= #aligned ESTs or mRNA-seq from an external GFF3 file

altest_gff= #aligned ESTs from a closly relate species in GFF3 format

#-----Protein Homology Evidence (for best results provide a file for at least one)

protein=ncbi-camelid-cow-old-maker-proteins.fa #protein sequence file in fasta format (i.e. from mutiple oransisms)

protein_gff= #aligned protein homology evidence from an external GFF3 file

#-----Repeat Masking (leave values blank to skip repeat masking)

model_org=all #select a model organism for RepBase masking in RepeatMasker

rmlib=dromedary-pbjelly-pilon-abyss-pilon-families.fa2 #provide an organism specific repeat library in fasta format for RepeatMasker

repeat_protein=/opt/maker/data/te_proteins.fasta #provide a fasta file of transposable element proteins for RepeatRunner

rm_gff= #pre-identified repeat elements from an external GFF3 file

prok_rm=0 #forces MAKER to repeatmask prokaryotes (no reason to change this), 1 = yes, 0 = no

softmask=1 #use soft-masking rather than hard-masking in BLAST (i.e. seg and dust filtering)

#-----Gene Prediction

snaphmm= #SNAP HMM file

gmhmm=dromedary-pbjelly-pilon-abyss-pilon-gmhmm.mod #GeneMark HMM file

augustus_species=BUSCO_dromedary-pbjelly-pilon-abyss-pilon #Augustus gene prediction species model

fgenesh_par_file= #FGENESH parameter file

pred_gff= #ab-initio predictions from an external GFF3 file

model_gff= #annotated gene models from an external GFF3 file (annotation pass-through)

est2genome=0 #infer gene predictions directly from ESTs, 1 = yes, 0 = no

protein2genome=0 #infer predictions from protein homology, 1 = yes, 0 = no

trna=0 #find tRNAs with tRNAscan, 1 = yes, 0 = no

snoscan_rrna= #rRNA file to have Snoscan find snoRNAs

unmask=0 #also run ab-initio prediction programs on unmasked sequence, 1 = yes, 0 = no

#-----Other Annotation Feature Types (features MAKER doesn't recognize)

other_gff= #extra features to pass-through to final MAKER generated GFF3 file

#-----External Application Behavior Options

alt_peptide=C #amino acid used to replace non-standard amino acids in BLAST databases

cpus=1 #max number of cpus to use in BLAST and RepeatMasker (not for MPI, leave 1 when using MPI)

#-----MAKER Behavior Options

max_dna_len=100000 #length for dividing up contigs into chunks (increases/decreases memory usage)

min_contig=10000 #skip genome contigs below this length (under 10kb are often useless)

pred_flank=200 #flank for extending evidence clusters sent to gene predictors

pred_stats=1 #report AED and QI statistics for all predictions as well as models

AED_threshold=1 #Maximum Annotation Edit Distance allowed (bound by 0 and 1)

min_protein=30 #require at least this many amino acids in predicted proteins

alt_splice=1 #Take extra steps to try and find alternative splicing, 1 = yes, 0 = no

always_complete=0 #extra steps to force start and stop codons, 1 = yes, 0 = no

map_forward=0 #map names and attributes forward from old GFF3 genes, 1 = yes, 0 = no

keep_preds=1 #Concordance threshold to add unsupported gene prediction (bound by 0 and 1)

split_hit=10000 #length for the splitting of hits (expected max intron size for evidence alignments)

single_exon=1 #consider single exon EST evidence when generating annotations, 1 = yes, 0 = no

single_length=250 #min length required for single exon ESTs if 'single_exon is enabled'

correct_est_fusion=0 #limits use of ESTs in annotation to avoid fusion genes

tries=2 #number of times to try a contig if there is a failure for some reason

clean_try=0 #remove all data from previous run before retrying, 1 = yes, 0 = no

clean_up=0 #removes theVoid directory with individual analysis files, 1 = yes, 0 = no

TMP= #specify a directory other than the system default temporary directory for temporary files

#####maker_opts.ctl#####

cd /genetics/elbers/CamDro1/maker

# Set Augustus to version 3.3

perl -pi -e "s/\/genetics\/elbers\/augustus\/bin/\/opt\/maker\/exe\/augustus\/bin/g" ~/.bashrc

perl -pi -e "s/\/genetics\/elbers\/augustus\/scripts/\/opt\/maker\/exe\/augustus\/scripts/g" ~/.bashrc

source ~/.bashrc

cpanm --local-lib=~/perl5 local::lib && eval $(perl -I ~/perl5/lib/perl5/ -Mlocal::lib)

/genetics/elbers/maker-2.31.10/exe/mpich2/bin/mpiexec -n 75 /opt/maker/bin/maker -fix_nucleotides >> CamDro1.fa.output.txt 2>&1

## second get the GFF3 sequences

cd /genetics/elbers/CamDro1/maker

/opt/maker/bin/gff3_merge -d CamDro1.maker.output/CamDro1_master_datastore_index.log -o CamDro1.all.gff -n

/opt/maker/bin/gff3_merge -d CamDro1.maker.output/CamDro1_master_datastore_index.log -o CamDro1.all.with.sequences.gff

## third get the FASTA sequences

/opt/maker/bin/fasta_merge -d CamDro1.maker.output/CamDro1_master_datastore_index.log -o CamDro1.all.fasta

## Determine the "best" cut-off value for AED (annotation edit distance)

wget https://raw.githubusercontent.com/mscampbell/Genome_annotation/master/AED_cdf_generator.pl

perl AED_cdf_generator.pl -b 0.025 CamDro1.all.gff > maker-run1.aed.cum.frac.below.txt

# AED 0.50 is "best" cut-off value as 0.392 of transcripts have AED <= 0.50

## fourth get transcript and protein sequences

### i get high quality proteins and transcripts

/opt/seqtk/seqtk seq -l0 CamDro1.all.fasta.all.maker.proteins.fasta | pcregrep --buffer-size 3000000000 -M " AED:0.[0-4][0-9].+\n\w+| AED:0.50.+\n\w+" \

> CamDro1.all.fasta.all.maker.proteins.0-0.50_AED.fasta

/opt/seqtk/seqtk seq -l0 CamDro1.all.fasta.all.maker.transcripts.fasta | pcregrep --buffer-size 3000000000 -M " AED:0.[0-4][0-9].+\n\w+| AED:0.50.+\n\w+" \

> CamDro1.all.fasta.all.maker.transcripts.0-0.50_AED.fasta

## ii run ideel

mkdir ideel

/opt/diamond/diamond_0.9.19 blastp --threads 75 --max-target-seqs 1 --db /genetics/elbers/maker/uniprot_trembl_release_2018_04.fasta \

--query CamDro1.all.fasta.all.maker.proteins.0-0.50_AED.fasta \

--outfmt 6 qlen slen --out ideel/CamDro1.all.fasta.all.maker.proteins.0-0.50_AED.fasta.against.uniprot_trembl_release_2018_04.fasta.blast > diamond.log 2>&1

echo -e 'test <- read.table("ideel/CamDro1.all.fasta.all.maker.proteins.0-0.50_AED.fasta.against.uniprot_trembl_release_2018_04.fasta.blast")\ntest2 <- test$V1/test$V2\ntest3 <- test2[test2 >= 0.85]\ntest4 <- test3[test3 <= 1.15]\ntest5 <- test2[test2 < 0.85]\ncat("\n")\npaste("There are",length(test2),"protein hits, and", length(test4), round(length(test4)/length(test2)*100,2), "%","are between 0.85 and 1.15 (querylength/subjectlength).")\ncat("\n")\npaste("There are", length(test5), round(length(test5)/length(test2)*100,2),"%", "protein hits less than 0.85 (querylength/subjectlength).")\ncat("\n")'> stats.R

Rscript stats.R > stats.txt 2>&1

## fifth make a id map for all genes in gff file (i.e., instead of maker-gene-01124356 convert to Cadr-000000001)

/opt/maker/bin/maker_map_ids --prefix Cadr_ --justify 8 CamDro1.all.gff > CamDro1.all.gff.id.map

## sixth blast high quality maker proteins against uniprot trembl

/opt/diamond/diamond_0.9.19 blastp --threads 75 --max-target-seqs 1 --db /genetics/elbers/maker/uniprot_trembl_release_2018_04.fasta \

--evalue 1e-6 --query CamDro1.all.fasta.all.maker.proteins.0-0.50_AED.fasta --outfmt 6 \

--out CamDro1.all.fasta.all.maker.proteins.fasta.blast > diamond.maker.log 2>&1

## seventh make copies of files (because scripts below overwrite input files)

cp CamDro1.all.gff CamDro1.all.renamed.gff

cp CamDro1.all.fasta.all.maker.proteins.0-0.50_AED.fasta CamDro1.all.fasta.all.maker.proteins.renamed.fasta

cp CamDro1.all.fasta.all.maker.transcripts.0-0.50_AED.fasta CamDro1.all.fasta.all.maker.transcripts.renamed.fasta

cp CamDro1.all.fasta.all.maker.proteins.fasta.blast CamDro1.all.fasta.all.maker.proteins.fasta.renamed.blast

## eighth rename the maker supplied gene names to ids made in the fifth step above

/opt/maker/bin/map_gff_ids CamDro1.all.gff.id.map CamDro1.all.renamed.gff

## ninth rename the maker supplied protein and transcript names to ids made in the fifth step above

/opt/maker/bin/map_fasta_ids CamDro1.all.gff.id.map CamDro1.all.fasta.all.maker.proteins.renamed.fasta

/opt/maker/bin/map_fasta_ids CamDro1.all.gff.id.map CamDro1.all.fasta.all.maker.transcripts.renamed.fasta

## tenth rename the maker supplied gene names in the BLAST search from sixth step above to ids made in the fifth step above

/opt/maker/bin/map_data_ids CamDro1.all.gff.id.map CamDro1.all.fasta.all.maker.proteins.fasta.renamed.blast

## eleventh annotate the genes

/opt/maker/bin/maker_functional_gff /genetics/elbers/maker/uniprot_trembl_release_2018_04.fasta CamDro1.all.fasta.all.maker.proteins.fasta.renamed.blast CamDro1.all.renamed.gff > CamDro1.all.renamed.annotated.gff

## twelfth retain only annotations from "maker", which is a combination of annotations from genemark and augustus

awk -F"\t" -v OFS="\t" '$1=="##gff-version 3"||$2=="maker"' CamDro1.all.renamed.annotated.gff > CamDro1.all.renamed.annotated.maker.gff

## thirteenth retain only gene annotations that are high quality (AED <= 0.50)

perl /opt/maker/src/quality_filter.pl -a 0.51 CamDro1.all.renamed.annotated.maker.gff > CamDro1.all.renamed.annotated.maker.qualityfilter.gff

## fourteenth make a gff file of just the genes (useful for IGV)

awk -F"\t" -v OFS="\t" '$1=="##gff-version 3"||$3=="gene"' CamDro1.all.renamed.annotated.maker.qualityfilter.gff > CamDro1.all.renamed.annotated.maker.qualityfilter.genes.only.gff

## fifteenth run GAG to genome stats

python2 /genetics/elbers/genomeannotation-GAG-997e384/gag.py \

--fasta CamDro1.fa \

--gff CamDro1.all.renamed.annotated.maker.qualityfilter.gff \

--out gag_output > gag.1.log 2>&1

## sixteenth annotate the proteins and transcripts

/opt/maker/bin/maker_functional_fasta /genetics/elbers/maker/uniprot_trembl_release_2018_04.fasta CamDro1.all.fasta.all.maker.proteins.fasta.renamed.blast CamDro1.all.fasta.all.maker.proteins.renamed.fasta > CamDro1.all.fasta.all.maker.proteins.renamed.annotated.fasta &

/opt/maker/bin/maker_functional_fasta /genetics/elbers/maker/uniprot_trembl_release_2018_04.fasta CamDro1.all.fasta.all.maker.proteins.fasta.renamed.blast CamDro1.all.fasta.all.maker.transcripts.renamed.fasta > CamDro1.all.fasta.all.maker.transcripts.renamed.annotated.fasta

##########ignore##########

samtools faidx /genetics/elbers/CamDro1/maker/CamDro1.fa

cut -f 1-2 /genetics/elbers/CamDro1/maker/CamDro1.fa.fai > test1

sort -nk2,2 test1 |awk '$2> 9999' > /genetics/elbers/CamDro1/maker/test2

sort -u /genetics/elbers/CamDro1/maker/CamDro1.maker.output/CamDro1_master_datastore_index.log |grep "FINISHED"|cut -f 1 > /genetics/elbers/CamDro1/maker/finished

perl -pe "s/(.+)/^\1\t/g" /genetics/elbers/CamDro1/maker/finished > /genetics/elbers/CamDro1/maker/finished2

LANG=C

grep -f /genetics/elbers/CamDro1/maker/finished2 /genetics/elbers/CamDro1/maker/test2 > /genetics/elbers/CamDro1/maker/test3

LANG=en_GB.UTF-8

basesdone="$(awk '{sum+=$2}END{print sum}' /genetics/elbers/CamDro1/maker/test3)"

basestotal="$(awk '{sum+=$2}END{print sum}' /genetics/elbers/CamDro1/maker/test2)"

echo

sort -u /genetics/elbers/CamDro1/maker/CamDro1.maker.output/CamDro1_master_datastore_index.log |cut -f 3 |sort |uniq -c

echo

bc <<< "scale=6; $basesdone/$basestotal*100"

grep "Error\|error\|ERROR\|Fail\|FAIL\|fail" /genetics/elbers/CamDro1/maker/CamDro1.fa.output.txt |less -S

##########ignore##########

#########################

# Step 32 Make dot plots with D-GENIES to compare CamDro2 to CamDro1 and compare CamDro2 to dnazoo.org's chromosomal Alpaca assembly

#########################

mkdir -p /genetics/pacbio/dot-plots

#

cd /genetics/pacbio/dot-plots

mkdir -p alpaca

wget https://www.dropbox.com/s/6gqx0iby3vbyo5g/Vicugna_pacos-2.0.1_HiC.fasta.gz

pigz -d Vicugna_pacos-2.0.1_HiC.fasta.gz

perl -pe "s/\t/\n/g" ../chromosomes > chromosomes.fa

# rev-compl #

1

3

4

6

7

8

9

10

12

13

14

26

35

# rev-compl#

# normal #

2

5

11

15

16

17

18

19

20

21

22

23

24

25

27

28

29

30

31

32

33

34

36

# normal #

while read i;do

samtools faidx chromosomes.fa ${i} |/opt/seqtk/seqtk seq -r > alpaca/${i}.fa

done < rev-compl

while read i;do

samtools faidx chromosomes.fa ${i} > alpaca/${i}.fa

done < normal

perl -pe "s/\t/\n/g" ../Xchromosome |/opt/seqtk/seqtk seq -r > alpaca/X.fasta

perl -pe "s/\t/\n/g" ../contigs > alpaca/contigs.fa

cd alpaca

cat 1.fa 2.fa 3.fa 4.fa 5.fa 6.fa 7.fa 8.fa 9.fa 10.fa 11.fa 12.fa 13.fa 14.fa 15.fa 16.fa 17.fa 18.fa 19.fa 20.fa 21.fa 22.fa 23.fa 24.fa 25.fa 26.fa 27.fa 28.fa 29.fa 30.fa 31.fa 32.fa 33.fa 34.fa 35.fa 36.fa X.fasta contigs.fa > ../CamDro2-rev-compl.fa

cd ..

wget https://raw.githubusercontent.com/genotoul-bioinfo/dgenies/v1.2.0/src/dgenies/bin/index.py

python index.py -i ../dromedary.pbjelly.pilon.abyss.pilon.chromosomes.fasta -n CamDro2 -o CamDro2.idx

python index.py -i Vicugna_pacos-2.0.1_HiC.fasta -n Alpaca -o Alpaca.idx

python index.py -i ../GCA_000803125.1_Cdrom64K_genomic.fna -n CamDro1 -o CamDro1.idx

perl -pe "s/CamDro2/CamDro2-chr-1-3-4-6-7-8-9-10-12-13-14-26-35-X-rev-compl/g" CamDro2.idx > CamDro2-rev-compl.idx

/genetics/elbers/minimap2-2.15_x64-linux/minimap2 -x asm5 CamDro2-rev-compl.fa Vicugna_pacos-2.0.1_HiC.fasta -t 75 > CamDro2-rev-compl-vs-alpaca.paf

# For D-GENIES to generate dot plot between CamDro2 and Alpaca

## Alignment file=CamDro2-rev-compl-vs-alpaca.paf

## Target file=CamDro2-rev-compl.idx

## Query file=Alpaca.idx

## In plotting results, used contig sorting function and filtering out matches with 0.001% dot plot width and identity ≤ 0.5

## Figure S5

## Figures S6-S9 are zoomed in views

/genetics/elbers/minimap2-2.15_x64-linux/minimap2 -x asm5 ../dromedary.pbjelly.pilon.abyss.pilon.chromosomes.fasta ../GCA_000803125.1_Cdrom64K_genomic.fna -t 75 > CamDro2-vs-CamDro1.paf

# For D-GENIES

## Alignment file=CamDro2-vs-CamDro1.paf

## Target file=CamDro2.idx

## Query file=CamDro1.idx

## In plotting results, used contig sorting function and filtering out matches with 0.001% dot plot width and identity ≤ 0.75

## Figure 3

#########################

# Step 33 Assign chromosomes to Alpaca assembly

#########################

cd /genetics/pacbio/dot-plots

# Assign chromosomes to Alpaca

/usr/bin/makeblastdb -dbtype nucl -in Vicugna_pacos-2.0.1_HiC.fasta

while read i;do

## blast the markers for each chromosome

/usr/bin/blastn -num_threads 75 -db Vicugna_pacos-2.0.1_HiC.fasta -query ../rhmarkers/chr${i}rhmarkers.txt -outfmt 6 -evalue 1e-30 -max_hsps 1 > alpaca/chr${i}rhmarkers.txt.blast

## count the BLAST hits for contigs/scaffolds

echo ${i} >> alpaca-contigs-to-chromosomes.txt

cut -f 1-2 alpaca/chr${i}rhmarkers.txt.blast |awk '!seen[$1]++'|cut -f 2 |sort |uniq -c|sort -n |tail -n 1|perl -pe "s/( )+/\t/g" |perl -pe "s/^\t//g" |cut -f 2 >> alpaca-contigs-to-chromosomes.txt

done < ../rhmarkers/samples

## modify numbers less than 9

perl -pi -e "s/^0//g" alpaca-contigs-to-chromosomes.txt

cat alpaca-contigs-to-chromosomes.txt|paste - - > alpaca-contigs-to-chromosomes2.txt

## Table S5 is alpaca-contigs-to-chromosomes2.txt

#########################

# Step 34 Get cumulative length distributions - Figure 2

#########################

cd /genetics/pacbio/dot-plots

/opt/bbmap/bbstats.sh in=../dromedary.pbjelly.pilon.abyss.pilon.chromosomes.fasta shist=CamDro2.hist

/opt/bbmap/bbstats.sh in=../GCA_000803125.1_Cdrom64K_genomic.fna shist=CamDro1.hist

/opt/bbmap/bbstats.sh in=../GCA_000767585.1_PRJNA234474_Ca_dromedarius_V1.0_genomic.fna.gz shist=Arabian-dromedary-assembly.hist

# Rscript to plot cumulative scaffold length #

library(ggplot2)

setwd("C:/Users/elbersj/Dropbox/camel_postdoc/manuscripts/dromedary-assembly/assembly-figures/")

df1<-read.table("CamDro1.hist")

df2<-read.table("CamDro2.hist")

df3<-read.table("Arabian-dromedary-assembly.hist")

scaffold.number <- c(df1$V1,df2$V1,df3$V1)

cumulative.length <- c(df1$V2,df2$V2,df3$V2)

set.seed(1)

df <- data.frame("scaffolds"=scaffold.number,

"lengths"=cumulative.length,

"assembly"=c(rep("CamDro1",length(df1$V1)),rep("CamDro2",length(df2$V1)),rep("Arabian dromedary",length(df3$V1))))

CamDro1 <- 2055063633

CamDro2 <- 2154386959

Arabian <- 2004047047

L50 <- data.frame("x"=c(11,132,393),

"y"=c(0.5*CamDro2,0.5*Arabian,0.5*CamDro1),

"assembly"=c("CamDro2","Arabian dromedary","CamDro1"))

L90 <- data.frame("x"=c(31,594,1592),

"y"=c(0.9*CamDro2,0.9*Arabian,0.9*CamDro1),

"assembly"=c("CamDro2","Arabian dromedary","CamDro1"))

plot2 <- ggplot(df, aes(scaffolds, lengths)) +

geom_line(aes(color=assembly), size=1.4) +

geom_point(data = L50, aes(x=x, y=y, color=assembly),size=4,shape=16) +

geom_point(data = L90, aes(x=x, y=y, color=assembly),size=4,shape=17) +

theme_bw() +

theme(panel.grid.major.x = element_blank(),

panel.grid.minor.x = element_blank(),

panel.grid.major.y = element_blank(),

panel.grid.minor.y = element_blank(),

panel.border = element_blank(),

axis.line = element_line(color="black"),

legend.position = c(0.15,0.85),

legend.title = element_blank(),

legend.text = element_text(size=12),

axis.text = element_text(size=12),

axis.title = element_text(size=14)) +

scale_x_continuous(trans='log10', breaks=c(1,3,10,30,100,300,1000,3000,10000,30000)) +

ylab("Cumulative length (bases)") +

xlab("Scaffold number (log10 scale)")

plot2

ggsave(filename = "dromedary-assembly-cumulative-length-distributions.png",device = "png", height = 6 , width = 7.5, dpi = 600,plot = plot2)

# Rscript to plot cumulative scaffold length #

# dromedary-assembly-cumulative-length-distributions.png is Figure 2

#########################

# Step 35 FigureS2 - Make spectra copy number plots for k-mer distributions of CamDro1 and CamDro2

#########################

library("ggforce")

library("gridExtra")

test1 <- read.table("C:/Users/elbersj/Dropbox/camel_postdoc/manuscripts/dromedary-assembly/k-mer/pe_vs_assembly_dromedary.pbjelly.pilon.abyss.pilon.chromosomes-main.mx")

test1 <- t(test1)

test1 <- test1[1:3,]

test1 = test1[1:3,1:60]

df <- data.frame("x"=c(1:60,1:60,1:60),

"y"=c(test1[1,1:60],test1[2,1:60],test1[3,1:60]),

"dup"=c(rep(0,60),rep(1,60),rep(2,60)))

df$dup <- as.factor(df$dup)

df$dup <- relevel(df$dup, "2")

df$dup <- relevel(df$dup, "1")

df$dup <- relevel(df$dup, "2")

p <- ggplot(df, aes(x = x, y = y,fill=dup)) +

scale_fill_manual(values=c("#ad7fa8", "#ef2929", "#000000")) +

geom_bar(stat='identity') +

facet_zoom(xlim = c(0, 15), ylim = c(0, 7500000), horizontal = FALSE) +

theme_bw() +

theme(panel.grid.major.x = element_blank(),

panel.grid.minor.x = element_blank(),

panel.grid.major.y = element_blank(),

panel.grid.minor.y = element_blank(),

panel.border = element_blank(),

axis.line = element_line(color="black"),

legend.position = "none",

legend.title = element_blank(),

legend.text = element_blank(),

axis.text = element_text(size=11),

axis.title = element_text(size=11))+

ylab("Number of distinct k-mers") +

xlab("k-mer multiplicity") +

annotate("text", x = 5, y =80000000, label = "B",size=6)

p

test2 <- read.table("C:/Users/elbersj/Dropbox/camel_postdoc/manuscripts/dromedary-assembly/k-mer/pe_vs_assembly_GCA_000803125.1_Cdrom64K_genomic-main.mx")

test2 <- t(test2)

test2 <- test2[1:3,]

test2 = test2[1:3,1:60]

df2 <- data.frame("x"=c(1:60,1:60,1:60),

"y"=c(test2[1,1:60],test2[2,1:60],test2[3,1:60]),

"dup"=c(rep(0,60),rep(1,60),rep(2,60)))

df2$dup <- as.factor(df2$dup)

df2$dup <- relevel(df2$dup, "2")

df2$dup <- relevel(df2$dup, "1")

df2$dup <- relevel(df2$dup, "2")

q <- ggplot(df2, aes(x = x, y = y,fill=dup)) +

scale_fill_manual(values=c("#ad7fa8", "#ef2929", "#000000")) +

geom_bar(stat='identity') +

facet_zoom(xlim = c(0, 15), ylim = c(0, 7500000), horizontal = FALSE) +

theme_bw() +

theme(panel.grid.major.x = element_blank(),

panel.grid.minor.x = element_blank(),

panel.grid.major.y = element_blank(),

panel.grid.minor.y = element_blank(),

panel.border = element_blank(),

axis.line = element_line(color="black"),

legend.position = "none",

legend.title = element_blank(),

legend.text = element_blank(),

axis.text = element_text(size=11),

axis.title = element_text(size=11))+

ylab("Number of distinct k-mers") +

xlab("k-mer multiplicity") +

annotate("text", x = 5, y =78000000, label = "A",size=6)

q

ggsave(filename = "FigureS2.png",device = "png", height = 6 , width = 6, dpi = 600,arrangeGrob(q, p,nrow=1))

**Supplemental Discussion**

The second MAKER run produced worse annotations (i.e., higher AEDs, more truncated proteins, and more proteins with unknown function) than the first MAKER run as made clear by the fact that BUSCO produced a superior *ab initio* gene model for Augustus than compared to Augustus’s autoAug.pl script (please see cDNA transcripts used to train Augustus in the file dromedary.pbjelly.pilon.abyss.pilon.all.maker.transcripts.cdna.for.augustus.training.zip in the Dryad repo doi 10.5061/dryad.6rp36b6) run on output of the first MAKER run. We tested this prediction by running Augustus on 75 sets of 250 random transcripts with AED ≤ 0.25 predicted by the first run of MAKER and found the BUSCO *ab initio* model had higher sensitivity and specificity at all levels than the autoAug.pl *ab initio* model. We also generated numerous additional *ab initio* models and evaluated their sensitivity and specificity (data not shown), but were unable to improve upon the BUSCO-generated *ab initio* Augustus model.

We recommend MAKER users evaluate successive MAKER runs, especially considering that Augustus *ab initio* models can actually become worse. Through a literature search of genome annotations using MAKER, we were only able to find a single paper where the authors compared AED between the consecutive runs. Martinez Barrio *et al*., [(2016)](https://paperpile.com/c/Heq3cT/QcYq/?noauthor=1) compared AEDs between two MAKER runs: one with only protein and transcript evidence and the other like the first but adding an *ab initio* model generated by training Augustus with the output of the first MAKER run. They observed lower annotation evidence distances when adding the *ab initio* Augustus model, which is similar to the strategy used in the present paper, but unlike Martinez Barrio *et al*., [(2016)](https://paperpile.com/c/Heq3cT/QcYq/?noauthor=1), we had already included an *ab initio* model in the first MAKER run generated by training Augustus with BUSCO. We found one other example in the literature that compared AEDs between different MAKER runs. Galachyants *et al*., [(2015)](https://paperpile.com/c/Heq3cT/ebV1/?noauthor=1) assessed the effect of different types of evidence on AEDs but did not assess differences between consecutive MAKER runs. There are many examples where authors have run MAKER iteratively [(Ullate-Agote *et al.*](https://paperpile.com/c/Heq3cT/rPX0+vjFk+1LpG+MQAb+3i6A+twZ5)*,* [2014; Bertioli *et al.*](https://paperpile.com/c/Heq3cT/rPX0+vjFk+1LpG+MQAb+3i6A+twZ5)*,* [2016; Braasch *et al.*](https://paperpile.com/c/Heq3cT/rPX0+vjFk+1LpG+MQAb+3i6A+twZ5)*,* [2016; Lee *et al.*](https://paperpile.com/c/Heq3cT/rPX0+vjFk+1LpG+MQAb+3i6A+twZ5)*,* [2016; Tørresen *et al.*](https://paperpile.com/c/Heq3cT/rPX0+vjFk+1LpG+MQAb+3i6A+twZ5)*,* [2017; Antonides *et al.*](https://paperpile.com/c/Heq3cT/rPX0+vjFk+1LpG+MQAb+3i6A+twZ5)*,* [2017)](https://paperpile.com/c/Heq3cT/rPX0+vjFk+1LpG+MQAb+3i6A+twZ5) as recommended by the developers of the program [(Campbell *et al.*](https://paperpile.com/c/Heq3cT/7ch3)*,* [2014a)](https://paperpile.com/c/Heq3cT/7ch3), but we suggest that each MAKER run should be evaluated to see if annotations actually improve.

**References**

[Altschul, S. (1990). Basic Local Alignment Search Tool. *Journal of Molecular Biology*, *215*, 403–410.](http://paperpile.com/b/Heq3cT/fjTlp)

Alim, F. Z. D., Romanova, E.V., Tay, Y-L., Rahman, AYbA., Chan, K-G., Hong K-W., Rogers, M., … Hindmarch, C. C. T. (2018). Seasonal adaptations of the hypothalamo-neurohypophyseal system of the dromedary camel. *PloS Biology* (Submitted)

Antonides, J., Ricklefs, R., & DeWoody, J. A. (2017). The genome sequence and insights into the immunogenetics of the bananaquit (Passeriformes: *Coereba flaveola*). *Immunogenetics*, 69, 175–186.

Avila, F., Baily, M. P., Perelman, P., Das, P. J., Pontius, J., Chowdhary, R., … Raudsepp, T. (2014). A comprehensive whole-genome integrated cytogenetic map for the alpaca (*Lama pacos*). Cytogenetic and Genome Research, 144, 196–207

Bertioli, D. J., Cannon, S. B., Froenicke, L., Huang, G., Farmer, A. D., Cannon, E. K. S., … Ozias-Akins, P. (2016). The genome sequences of *Arachis duranensis* and *Arachis ipaensis*, the diploid ancestors of cultivated peanut. *Nature Genetics*, 48, 438–446.

Braasch, I., Gehrke, A. R., Smith, J. J., Kawasaki, K., Manousaki, T., Pasquier, J., … Postlethwait, J. H. (2016). The spotted gar genome illuminates vertebrate evolution and facilitates human-teleost comparisons. *Nature Genetics*, 48, 427–437.

Cabanettes F., Klopp, C. (2018). D-GENIES: dot plot large genomes in an interactive, efficient and simple way. *PeerJ,* 6, e4958.

Campbell, M. S., Holt, C., Moore, B., & Yandell, M. (2014a). Genome annotation and curation using MAKER and MAKER-P. *Current Protocols in Bioinformatics*, 48, 4.11.1–39.

[Fitak, R. R., Mohandesan, E., Corander, J., & Burger, P. A. (2016). The *de novo* genome assembly and annotation of a female domestic dromedary of North African origin. *Molecular Ecology Resources*, *16*, 314–324.](http://paperpile.com/b/Heq3cT/NAIKs)

Galachyants, Y. P., Zakharova, Y. R., Petrova, D. P., Morozov, A. A., Sidorov, I. A., Marchenkov, A. M., … Grachev, M. A. (2015). Sequencing of the complete genome of an araphid pennate diatom *Synedra acus* subsp. *radians* from Lake Baikal. Doklady. *Biochemistry and Biophysics*, 461, 84–88.

Geib, S. M., Hall, B., Derego, T., Bremer, F. T., Cannoles, K., Sim, S. B. (2018). Genome Annotation Generator: a simple tool for generating and correcting WGS annotation tables for NCBI submission. *GigaScience*, 7, giy018.

Lee, H., Golicz, A. A., Bayer, P. E., Jiao, Y., Tang, H., Paterson, A. H., … Edwards, D. (2016). The genome of a southern hemisphere seagrass species (*Zostera muelleri*). *Plant Physiology*, 172, 272–283.

Li, H. (2018). Minimap2: pairwise alignment for nucleotide sequences. *Bioinformatics,* 34, 3094–3100.

Martinez Barrio, A., Lamichhaney, S., Fan, G., Rafati, N., Pettersson, M., Zhang, H., … Andersson, L. (2016). The genetic basis for ecological adaptation of the Atlantic herring revealed by genome sequencing. *eLife*, 5. doi:10.7554/eLife.12081

[Simão, F. A., Waterhouse, R. M., Ioannidis, P., Kriventseva, E. V., & Zdobnov, E. M. (2015). BUSCO: assessing genome assembly and annotation completeness with single-copy orthologs. *Bioinformatics*, *31*, 3210–3212.](http://paperpile.com/b/Heq3cT/e3FND)

[Stanke, M., Keller, O., Gunduz, I., Hayes, A., Waack, S., & Morgenstern, B. (2006). AUGUSTUS: *ab initio* prediction of alternative transcripts. *Nucleic Acids Research*, *34*, W435–9.](http://paperpile.com/b/Heq3cT/IHv53)

Tørresen, O. K., Star, B., Jentoft, S., Reinar, W. B., Grove, H., Miller, J. R., … Nederbragt, A. J. (2017). An improved genome assembly uncovers prolific tandem repeats in Atlantic cod. *BMC Genomics*, 18, 95.

Ullate-Agote, A., Milinkovitch, M. C., & Tzika, A. C. (2014). The genome sequence of the corn snake (*Pantherophis guttatus*), a valuable resource for EvoDevo studies in squamates. *The International Journal of Developmental Biology*, 58, 881–888.

[Zdobnov, E. M., Tegenfeldt, F., Kuznetsov, D., Waterhouse, R. M., Simão, F. A., Ioannidis, P., … Kriventseva, E. V. (2017). OrthoDB v9.1: cataloging evolutionary and functional annotations for animal, fungal, plant, archaeal, bacterial and viral orthologs. *Nucleic Acids Research*, *45*, D744–D749.](http://paperpile.com/b/Heq3cT/cCJ8O)
